# Ultrafast Excited-State Dynamics of Carotenoids and the Role of the S_*X*_ State

**DOI:** 10.1021/acs.jpclett.2c01555

**Published:** 2022-07-19

**Authors:** Davide Accomasso, Serra Arslancan, Lorenzo Cupellini, Giovanni Granucci, Benedetta Mennucci

**Affiliations:** Dipartimento di Chimica e Chimica Industriale, University of Pisa, via G. Moruzzi 13, 56124 Pisa, Italy

## Abstract

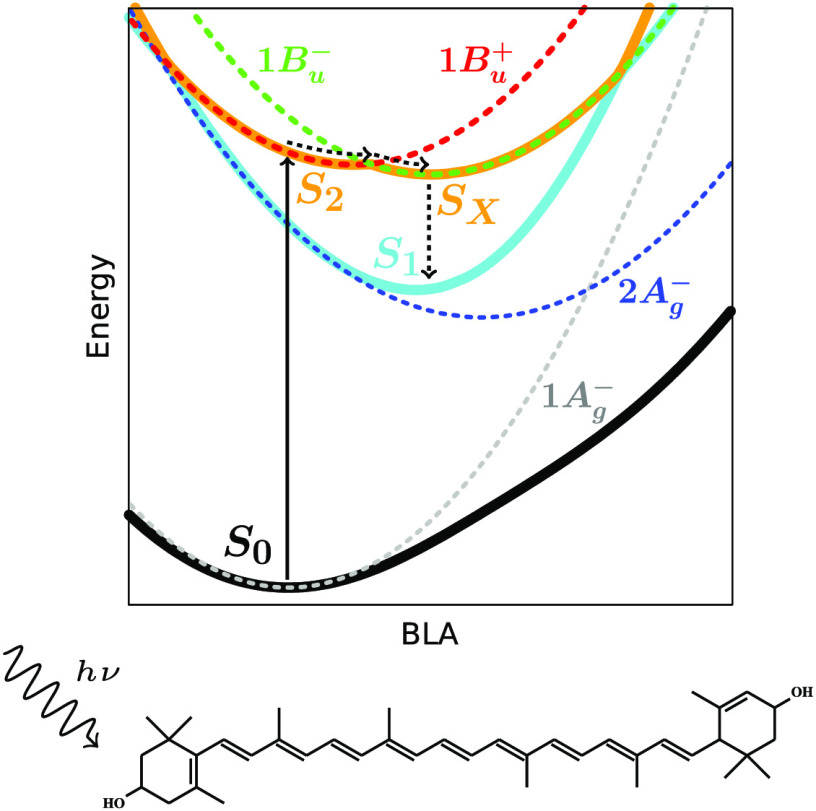

Carotenoids are natural pigments with multiple roles in photosynthesis. They act as accessory pigments by absorbing light where chlorophyll absorption is low, and they quench the excitation energy of neighboring chlorophylls under high-light conditions. The function of carotenoids depends on their polyene-like structure, which controls their excited-state properties. After light absorption to their bright S_2_ state, carotenoids rapidly decay to the optically dark S_1_ state. However, ultrafast spectroscopy experiments have shown the signatures of another dark state, termed S_*X*_. Here we shed light on the ultrafast photophysics of lutein, a xanthophyll carotenoid, by explicitly simulating its nonadiabatic excited-state dynamics in solution. Our simulations confirm the involvement of S_*X*_ in the relaxation toward S_1_ and reveal that it is formed through a change in the nature of the S_2_ state driven by the decrease in the bond length alternation coordinate of the carotenoid conjugated chain.

In photosynthetic organisms, carotenoids act as accessory pigments in light-harvesting (LH) complexes by absorbing in regions of the visible spectrum where the absorption of the main pigments (chlorophylls or bacteriochlorophylls) is not efficient. Moreover, carotenoids play a crucial role in photoprotective processes, and they allow a proper assembly of the LH complexes by stabilizing their structure.^1−3^ The multiple roles that carotenoids play in LH complexes are made possible by their polyene-like structure and the specificity of their electronically excited states.^4−7^ The light absorption of carotenoids involves the second singlet excited state (S_2_) because the transition to the lowest (S_1_) state is forbidden. By considering the approximate *C*_2*h*_ symmetry of the polyene chains, S_2_ has *B*_*u*_^+^ symmetry and is dominated by a single electron promotion from the HOMO (gerade, g) to the LUMO (ungerade, u) orbital, and S_1_ (∼*A*_*g*_^–^) is mainly a HOMO → LUMO double excitation.^4,8^ Upon photoexcitation, the generated S_2_ state decays in a few hundred femtoseconds to S_1_, which then relaxes back to S_0_ on a picosecond time scale.^8^

During the years, this two-state decay model has been enriched with additional (dark) states, which have been proposed to explain spectroscopic observations, and should lie in between the S_2_ and S_1_ states.^9−18^ In particular, a state, commonly indicated as S_*X*_, was initially detected as an intermediate in the S_2_–S_1_ decay of carotenoids in organic solvents, but then its signature was also observed in LH complexes.^16,19^ Its energy position with respect to S_2_ and S_1_ was shown to be dependent on the number (*N*) of conjugated CC double bonds of the carotenoid, and the S_*X*_ features were assigned to an electronic state of *B*_*u*_^–^ symmetry. Further studies have also shown that the activation of S_*X*_ as a decay intermediate is also affected by the environment.^15^

The unique properties of polyenes have always attracted the interest of quantum chemical investigations, and many different levels of theory have been used to clarify the nature of their electronic states and simulate their photophysical properties.^20−35^ On the contrary, much less has been done for what concerns the explicit simulation of the excited-state relaxation following the photoexcitation to the S_2_ state.^36−39^ This lack of dynamic investigations has prevented a clear theoretical confirmation of the involvement of additional dark states in the excited-state decay.

Here, we focus on lutein (*N* = 10), which belongs to the xanthophyll class of carotenoids because it contains one hydroxyl group in each of its terminal rings. Lutein is among the most abundant carotenoids in nature because it is present in the major LH complex (LHCII) of photosystem II in plants.^40^ Lutein is required for proper LHCII folding and organization.^41^ In addition to extending the absorption window of the LHCII complex, lutein also plays a fundamental role in the so-called nonphotochemical quenching (NPQ), the photoprotective strategy used under excess light to dissipate the energy absorbed by chlorophylls into harmless heat.^3,42−45^

To unveil the ultrafast decay pathway of the S_2_ state of lutein and characterize the intermediate S_*X*_ state, we use nonadiabatic excited-state dynamics based on the mixed quantum-classical surface hopping (SH) method.^46^ To the best of our knowledge, this kind of computational investigation for carotenoids is still missing in the literature. In fact, the molecular dimensions of the carotenoids, the number of correlated electrons, and the presence of states with different character make ab initio descriptions extremely costly and unsuitable for nonadiabatic dynamics.

Because the main features of the ultrafast dynamics of lutein have been measured to be similar in protein and in solution,^13,17,19^ here we model a methanol solution. To account for the effects of the solvent molecules, we use a quantum mechanics/molecular mechanics (QM/MM) description (Figure 1). The electronic structure of lutein is described by a semiempirical configuration interaction technique,^47,48^ which allows a balanced description of all excited states on the same grounds. The semiempirical parameters are specifically optimized for the system under study. (See Methods for more details.)

**Figure 1:**
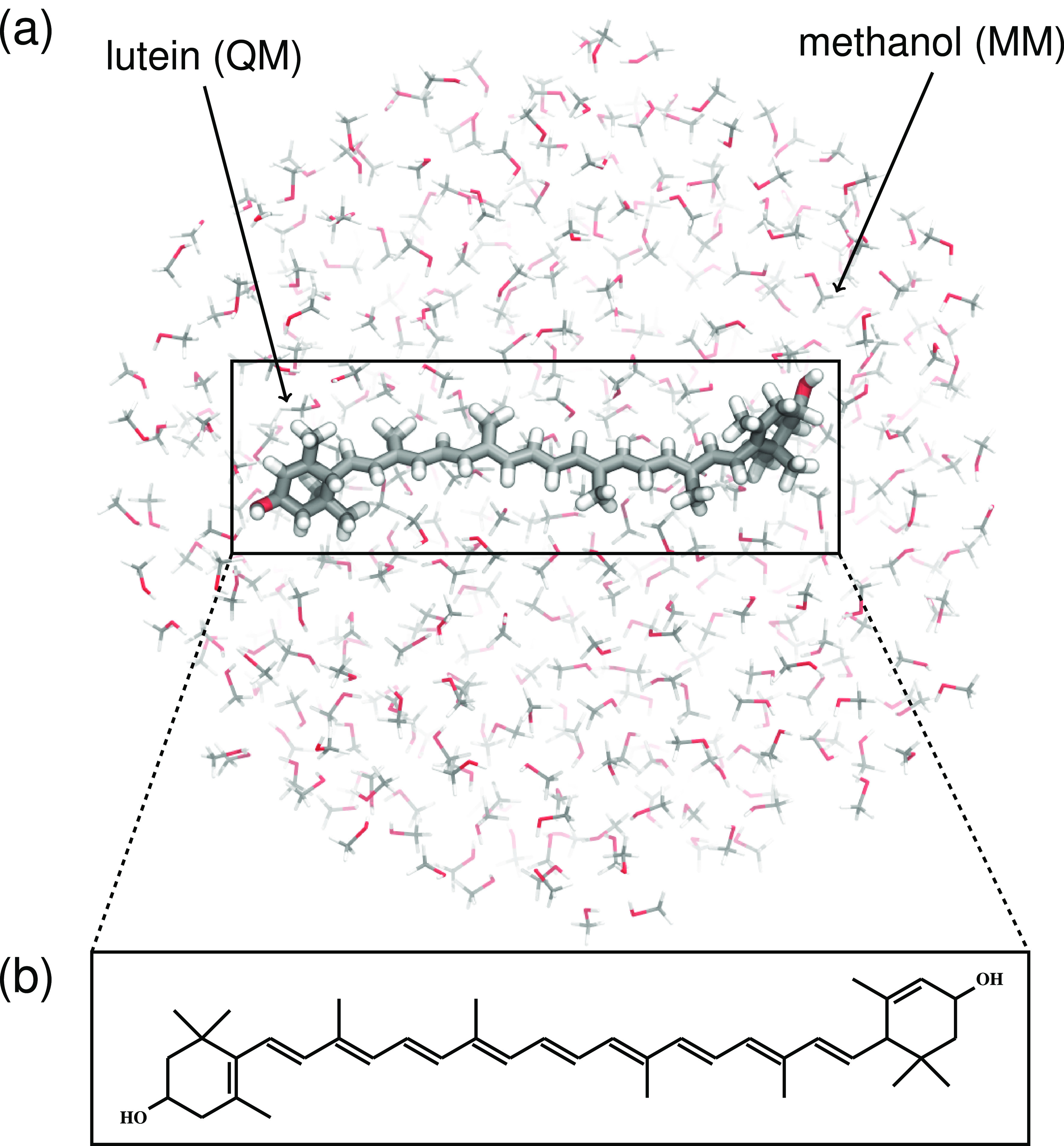
(a) Quantum mechanics/molecular mechanics (QM/MM) system used in the simulations. The QM lutein is shown in a licorice representation, and the MM methanol molecules are represented by thin lines. (b) Molecular structure of lutein.

As a preliminary analysis, we computed the three lowest excited states (S_1_–S_3_) for an isolated lutein in its ground-state (S_0_) minimum geometry and compared it to the analogous but symmetric C_20_H_22_ polyene (Table 1). In both molecules, the lowest excited state presents double excitation character (2*A*_*g*_^–^ for the symmetric system), and the second is mostly a HOMO–LUMO excitation (1*B*_*u*_^+^). Notably, the third excited state (1*B*_*u*_^–^) is only 0.22 eV higher in energy than the second. Although 1*B*_*u*_^–^ was not explicitly considered in the parametrization of our semiempirical method, this result is in agreement with previous DFT/MRCI calculations.^13^ Moreover, *ab initio* DMRG/MRPT2 calculations have suggested that the adiabatic 1*B*_*u*_^–^ energy is lower than the 1*B*_*u*_^+^ energy for carotenoids with eight or more double bonds.^35^ A similar result is suggested by experimental Raman excitation profiles.^49^ Other excited states are significantly higher in energy.

**Table 1:** Main Electronic Configurations in the Ground and the Three Low-Lying Singlet States of Lutein and the All-trans Linear Polyene with 10 Double Bonds (C_20_H_22_) at their S_0_ Minimum Geometry (Point Groups *C*_1_ for Lutein and *C*_2*h*_ for C_20_H_22_), Computed with the R-AM1/FOMO-CASCI(6,6) Method^a^

		weight		energy (eV)		oscilator strength	
state	configuration	lutein	C_20_H_22_	lutein	C_20_H_22_	lutein	C_20_H_22_
S_0_(1*A*_*g*_^–^)	···h, h	0.845	0.857	0.00	0.00		
S_1_(2*A*_*g*_^–^)	h, h → l, l	0.360	0.367	2.19	2.26	0.000	0.000
	h → l + 1	0.171	0.174				
	h – 1 → l	0.161	0.158				
S_2_(1*B*_*u*_^+^)	h → l	0.826	0.826	2.62	2.60	2.865	3.247
S_3_(1*B*_*u*_^–^)	h, h → l, l + 1	0.232	0.234	2.84	2.97	0.002	0.000
	h → l + 2	0.214	0.230				
	h – 1, h → l, l	0.209	0.208				
	h – 2 → l	0.173	0.170				

a The states are labeled according to their (pseudo)symmetry. For each configuration, the corresponding weight in the electronic wavefunction is reported. The complete list of CI coefficients, in the determinant basis, for each state of lutein is provided in Table S10. State energies relative to the ground state and the oscillator strengths are also reported. In the definition of the electronic configurations, we used h to indicate HOMO and l to indicate LUMO.

To confirm the robustness of these findings with respect to geometrical distortions, we sampled the Franck–Condon region of the solvated lutein through QM/MM S_0_ dynamics at room temperature. The vertical excitation energies calculated along the trajectory (Figure S5) confirm a close separation of the S_2_ and S_3_ states, whereas higher excited states remain well separated. While S_2_ is generally the brightest state, S_3_ acquires dipole strength (Figure S7), which suggests a mixing of these two states.

Starting from the S_0_ initial conditions, we propagated 200 SH trajectories by populating the excited states according to their radiative transition probability within an energy interval (see Methods for more details). Initially, S_2_ is the most populated state, but S_3_ is also significantly populated. In Figure 2a, we report the adiabatic state populations as functions of time obtained from the QM/MM surface-hopping simulations.

**Figure 2:**
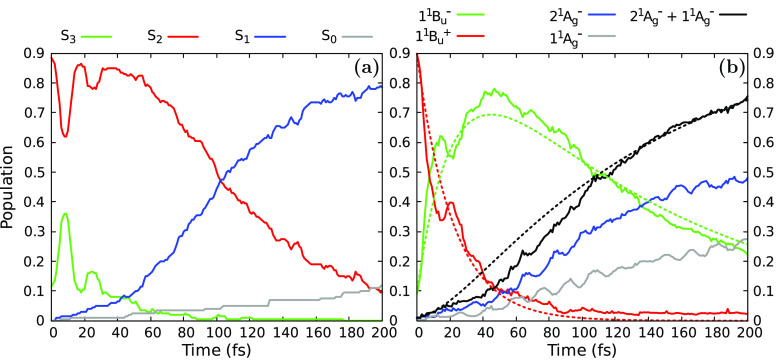
Adiabatic (panel a) and diabatic (panel b) state populations as functions of time obtained from the simulations of excited-state dynamics for lutein in methanol solution. The reported results are obtained by averaging over all trajectories and time intervals of 1 fs. In panel b, the fitting functions for the diabatic populations are also shown (dashed lines, see eqs S7–S9 in Section S3.3). The extracted time constants are τ_2*x*_ = 21.8 fs and τ_*x*1_ = 132.3 fs.

In our simulations, the photogenerated population of S_2_ decays to S_1_ within 200 fs. The main relaxation pathway toward S_1_ involves a direct transition (Table 2). However, the population decay of S_2_ is clearly nonexponential, and strong population oscillations occur between S_2_ and S_3_ within the first 50 fs of the photoexcitation (Figure 2a). In all cases, the S_1_ state is directly populated by transitions from S_2_ (Tables 2 and S7).

**Table 2:** Main Relaxation Pathways in Both the Adiabatic Basis and the Diabatic One Obtained in the Excited-State Simulations of Lutein in Methanol Solution^a^

adiabatic basis	no. traj.	%
S_2_ → S_1_	70	35.0
S_2_ → S_3_ → S_2_ → S_1_	31	15.5
S_2_ → S_3_ → S_2_ → S_3_ → S_2_ → S_1_	14	7.0
S_3_ → S_2_ → S_1_	12	6.0
S_2_ → S_1_ → S_0_	11	5.5

diabatic basis	no. traj.	%
1*B*_*u*_^+^ → 1*B*_*u*_^–^ → 2*A*_*g*_^–^ + 1*A*_*g*_^–^	58	29.0
1*B*_*u*_^+^ → 1*B*_*u*_^–^ → 1*B*_*u*_^+^ → 1*B*_*u*_^–^ → 2*A*_*g*_^–^ + 1*A*_*g*_^–^	27	13.5
1*B*_*u*_^+^ → 1*B*_*u*_^–^ → 1*B*_*u*_^+^ → 1*B*_*u*_^–^ → 1*B*_*u*_^+^ → 1*B*_*u*_^–^ → 2*A*_*g*_^–^ + 1*A*_*g*_^–^	14	7.0

a For the diabatic basis, the pathways towards the 2*A*_*g*_^–^ and 1*A*_*g*_^–^ states are grouped together. For each identified pathway, the total number of surface-hopping trajectories and the corresponding percentages are also reported. Only the pathways with percentages above 5.0% are reported. The complete lists of pathways are reported in Tables S7 and S8.

To better characterize the nature of the electronic states involved in the dynamics and their changes along the relaxation, we introduced a diabatic description. The diabatic states are defined so that they maximally resemble the lowest adiabatic singlet states of lutein (S_0_–S_3_) at the S_0_ minimum geometry and are indicated using their (pseudo)symmetry: 1*A*_*g*_^–^, 2*A*_*g*_^–^, 1*B*_*u*_^+^, and 1*B*_*u*_^–^ (see Table 1 and Methods for more details on the diabatization).

The computed populations of diabatic states along the relaxation are reported in Figure 2b. Diabatic populations reveal a strikingly different picture with respect to adiabatic ones: here, in fact, the initially most populated 1*B*_*u*_^+^ bright state rapidly transfers its population to the 1*B*_*u*_^–^ dark state. The 1*B*_*u*_^–^ state becomes lower in energy and can be identified with S_2_ in most of the trajectories. At variance with the adiabatic populations, analyzing diabatic states allows a clear identification of 1*B*_*u*_^–^ as intermediate in the relaxation. The 1*B*_*u*_^+^ → 1*B*_*u*_^–^ transfer of population is ultrafast and occurs in the first ∼50 fs of our simulations. At longer times, 1*B*_*u*_^–^ starts transferring its population to the lower-lying 2*A*_*g*_^–^ and 1*A*_*g*_^–^ states, which are the most populated states at the end of the simulations (*t* = 200 fs). We see in Table 2 that the main relaxation mechanism is 1*B*_*u*_^+^ → 1*B*_*u*_^–^ → 2*A*_*g*_^–^ + 1*A*_*g*_^–^.

We also identified several minor pathways involving multiple transitions back and forth between 1*B*_*u*_^+^ and 1*B*_*u*_^–^ (Tables 2 and S8). This indicates the oscillation of population between these two states as their crossing seam is traversed multiple times by the nuclear trajectory (see below). The 2*A*_*g*_^–^ + 1*A*_*g*_^–^ states are mainly populated by transitions from 1*B*_*u*_^–^, and the 1*B*_*u*_^+^ → 2*A*_*g*_^–^ + 1*A*_*g*_^–^ direct transfer of population represents a negligible route: the 1*B*_*u*_^+^ → 2*A*_*g*_^–^ transition was observed in only 3 trajectories (out of 200), and no direct transition from 1*B*_*u*_^+^ to 1*A*_*g*_^–^ was identified (Tables S5 and S8).

In Figure 3, we report the energies of the three low-lying diabatic excited states and the bond-length alternation (BLA) coordinate, averaged over all of the SH trajectories, as a function of time (see Figure S9 for the adiabatic energies). Here, the BLA is defined as the average difference between single- and double-bond lengths in the π-conjugated system. Throughout the ultrafast dynamics, the energies of the excited states undergo high-frequency fluctuations, which are generated by oscillations of the BLA. Notably, the 1*B*_*u*_^–^ energy is more sensitive than the 1*B*_*u*_^+^ energy to the BLA change, which causes a swap between the two states at the beginning of the dynamics. These observations suggests that the entire relaxation to 2*A*_*g*_^–^ occurs along the BLA coordinate and is driven by the high-frequency C=C and C–C stretching modes of the polyene chain of lutein. The oscillations show a period of ∼20 fs, which corresponds to ∼1700 cm^–1^, compatible with the C=C frequencies, although slightly overestimated by the semiempirical method. Overall, the BLA coordinate undergoes a significant decrease during the ultrafast dynamics, indicating the shortening of the C–C bonds and the corresponding elongation of the conjugated C=C bonds.

**Figure 3:**
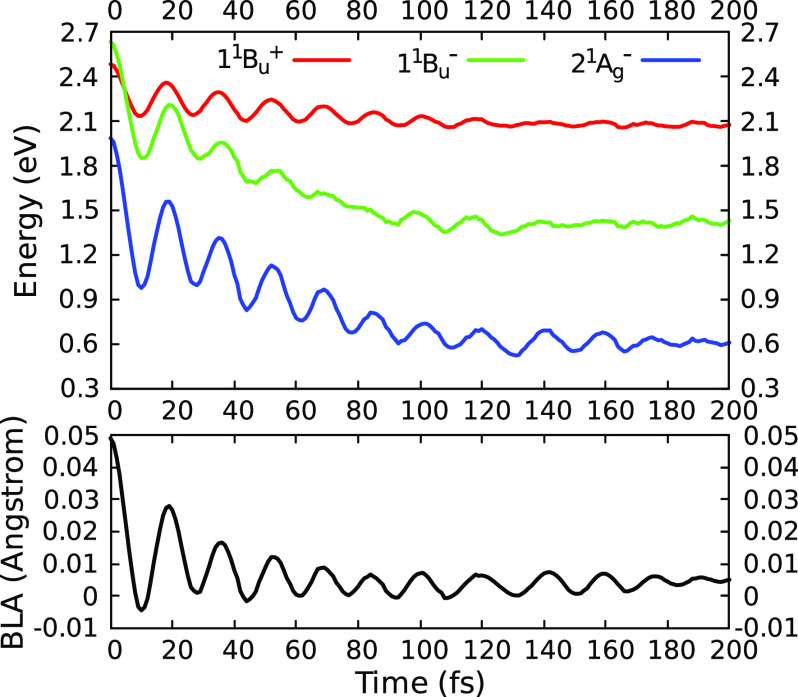
Energies (eV) of the three low-lying diabatic excited states relative to the diabatic ground state (1*A*_*g*_^–^) and bond-length alternation (BLA, Å) as functions of time. The reported results are obtained by averaging over all trajectories and time intervals of 1 fs.

To get a more detailed description of the relaxation pathways, we calculated the average energy gaps, electronic couplings, and values of BLA both at the Franck–Condon point (*t* = 0) and at the transitions between pairs of diabatic states. All of the values are reported in Table S4 of the SI.

Notably, the 1*B*_*u*_^+^ ↔ 1*B*_*u*_^–^ transitions occur at geometries where the energy gap between the two states is nearly zero. This quasi-degeneracy condition is reached through a significant decrease in the BLA, namely, from ∼0.05 Å at the initial geometries to ∼0.025 Å at the transition. Such transition geometries are characterized by large values of the S_2_/S_3_ nonadiabatic coupling (Figure S10). On the other hand, the average electronic couplings between 1*B*_*u*_^+^ and 1*B*_*u*_^–^ at their transition geometries are small and similar to the values at the Franck–Condon point. Contrary to the 1*B*_*u*_^+^ ↔ 1*B*_*u*_^–^ transitions, the jumps from 1*B*_*u*_^–^ to 2*A*_*g*_^–^ occur at geometries where the energy gap between states is quite large, i.e., ∼0.64 eV on average, a value very close to the one calculated at the initial geometries. However, now the transitions occur with a larger electronic coupling: ∼100 meV compared to around 60 meV at the Franck–Condon point. There is also a further decrease in the BLA, which averages to 0.002 Å at the 1*B*_*u*_^–^ → 2*A*_*g*_^–^ transition geometries.

Comparing Figure 2a,b, one can notice that the 2*A*_*g*_^–^ and 1*A*_*g*_^–^ populations differ significantly from the populations of the S_1_ and S_0_ states, respectively, especially toward the end of the simulations. In addition, the two *A*_*g*_^–^ populations seem to follow the same time evolution. This is caused by a strong mixing of the 2*A*_*g*_^–^ and 1*A*_*g*_^–^ states when lutein approaches the S_1_ minimum. There, the S_1_ wave function, while dominated by the 2*A*_*g*_^–^ diabatic state, acquires an important contribution from 1*A*_*g*_^–^. We quantified the electronic coupling between 2*A*_*g*_^–^ and 1*A*_*g*_^–^ at the geometries where S_1_ is the active state of the SH trajectories. The mean absolute value is ∼550 meV, much larger than the ∼80 meV at the Franck–Condon point (Table S6). The two *A*_*g*_^–^ states are also much closer in energy, with an average energy gap of ∼0.5 eV. The strong mixing makes the adiabatic S_0_ and S_1_ PESs much different from their diabatic counterparts. Given this mixing, in the diabatic analysis we have considered the two *A*_*g*_^–^ states together.

We quantified the characteristic times for the excited-state transitions by fitting the diabatic state populations in Figure 2b through a simple kinetic model. We assumed that the state populations obey the kinetic scheme 1*B*_*u*_^+^ → 1*B*_*u*_^–^ → 2*A*_*g*_^–^ + 1*A*_*g*_^–^ with fixed rates. (See Section S3.3 for more details.) The fitting functions (eqs S7–S9) are shown in Figure 2b by dashed lines and well fit the state populations, with the exception of the oscillations at around 20 fs. We obtain the time constants τ_2*x*_ = 21.8 fs for the 1*B*_*u*_^+^ → 1*B*_*u*_^–^ transition and τ_*x*1_ = 132.3 fs for the 1*B*_*u*_^–^ → 2*A*_*g*_^–^ + 1*A*_*g*_^–^ transition. These time constants also agree with the mean transition times reported in Table S4, confirming that the kinetic model well describes the population evolution.

Before summarizing the ultrafast evolution of lutein, we compare the results obtained so far with the excited-state dynamics of gas-phase lutein. (See Section S5 for the computational details.) Figure S12 shows the adiabatic and diabatic state populations as functions of time. The evolution is qualitatively very similar to the solution one shown in Figure 2. However, in the gas phase we observe more pronounced oscillations of the S_2_ and S_3_ populations during the first ∼50 fs and a slower excited-state decay to S_1_. The ultrafast relaxation mechanism identified in methanol is preserved in our gas-phase simulations. Specifically, in the adiabatic picture the S_2_ state exchanges population with S_3_ before decaying to S_1_, and in the diabatic basis, the 1*B*_*u*_^–^ state mediates the ultrafast decay 1*B*_*u*_^+^ → 2*A*_*g*_^–^ + 1*A*_*g*_^–^. The extracted lifetimes for the 1*B*_*u*_^+^ and 1*B*_*u*_^–^ states in vacuum are τ_2*x*_ = 49 fs and τ_*x*1_ = 171 fs, respectively, which are slightly longer than the ones in methanol. The larger internal conversion rate in methanol, compared to the gas-phase simulations, can be mainly attributed to the faster 1*B*_*u*_^+^ → 1*B*_*u*_^–^ transfer of population, which in turn is due to the larger 1*B*_*u*_^+^/1*B*_*u*_^–^ average coupling, both at the starting geometries and at the 1*B*_*u*_^+^ → 1*B*_*u*_^–^ transitions (compare Tables S4 and S13). Because the gas phase and methanol can be taken as two limits of low and high polarity, this result suggests a decrease in ultrafast relaxation times with increasing solvent polarity.

We can now characterize the detailed mechanism for the ultrafast excited-state dynamics of lutein. To this end, we make use of the potential energy curves calculated for the four low-lying states as functions of BLA (Figure 4). These curves have been obtained by fitting the diabatic PESs along a relaxed scan on the S_0_ of lutein (Figure S8).

**Figure 4:**
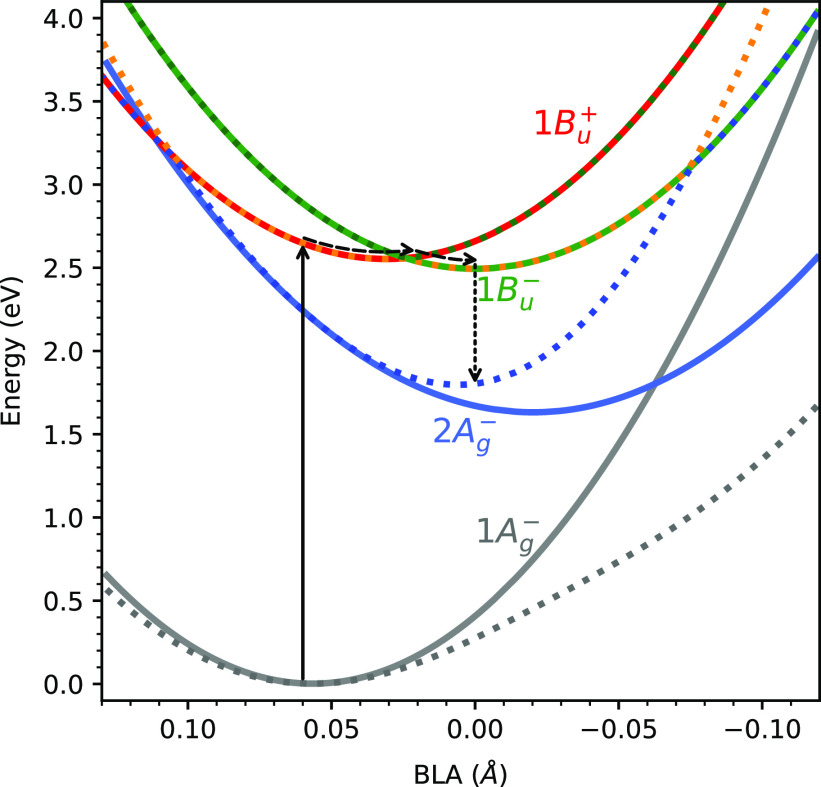
Energies (eV) of the four low-lying diabatic (solid lines) and adiabatic (dashed lines) states as functions of BLA (Å) obtained by fitting the diabatic PESs along a relaxed scan on the S_0_ state of lutein in vacuum (Figure S8). A schematic representation of the main relaxation pathway identified in the QM/MM surface hopping simulations is also shown. Note that the BLA axis is inverted for an easier interpretation of the mechanism.

Upon photoexcitation, the system, initially in the 1*B*_*u*_^+^ bright state, is rapidly driven toward the crossing between the 1*B*_*u*_^+^ and 1*B*_*u*_^–^ PESs and changes its electronic character while remaining in the S_2_ surface. Then, the nuclear wavepacket is divided among these two states, and the electronic population is split between the upper (S_3_) and lower (S_2_) surfaces. Multiple crossings can occur at this point between the two surfaces, causing the electronic populations to oscillate as observed in Figure 2. Finally, once the 1*B*_*u*_^–^ state is populated, the system relaxes to the S_1_/2*A*_*g*_^–^ state.

Comparing the diabatic to the adiabatic PESs (dashed lines in Figure 4) also reveals the large electronic mixing between the two *A*_*g*_^–^ states, as the adiabatic S_0_ and S_1_ PESs strongly deviate from the diabatic counterparts as soon as the BLA decreases. This indicates sizable vibronic mixing along the BLA coordinate, which generates a widely avoided crossing between the two surfaces and alters the curvature of S_0_ and S_1_ PESs. Specifically, the S_0_ curvature is decreased, whereas the S_1_ curvature is increased. This effect causes a downshift in the vibrational frequencies of the C=C stretching mode in the S_0_ state and a frequency upshift in the S_1_ state.^50,51^ We have monitored the frequency upshift by comparing the BLA power spectrum calculated with the S_0_ Born–Oppenheimer dynamics with the one calculated on those SH trajectories that remain in the S_1_ state for at least 175 fs (Figure S11). In the S1 state, the BLA power spectrum features a peak at substantially higher frequencies compared to the S_0_ dynamics.

Our results show that the 1*B*_*u*_^–^ state indeed mediates the S_2_ → S_1_ internal conversion by changing the electronic character of the S_2_ surface. We can then identify 1*B*_*u*_^–^ with the spectroscopically detected S_*X*_ dark state. This assignment also supports the main interpretations of the ultrafast spectroscopy of lutein in solution.^13,17^

In particular, Miki et al.^17^ investigated the vibrational dynamics in the electronic excited states by pump-degenerate four-wave mixing and observed a frequency downshift of the C=C and C–C stretching modes in the first 200 fs after the photoexcitation. This effect was ascribed to the strong diabatic mixing between 1*B*_*u*_^+^ and 1*B*_*u*_^–^ states. The reported values for the decay time of the S_2_ state are between 20 and 50 fs, depending on the solvent, while the extracted lifetime of S_*X*_ is ≲100 fs.^17^ These decay times agree well with our simulations, suggesting that the observed ultrafast decay of lutein is indeed mediated by the 1*B*_*u*_^–^ state. In the combined experimental and theoretical investigation by Ostroumov et al.,^13^ the authors observed large-amplitude oscillations in the transient absorption signals, which were attributed to electronic quantum beats caused by the coherent excitation of the strongly coupled 1*B*_*u*_^+^ and 1*B*_*u*_^–^ states. The damping of the quantum beats was observed with decoherence times well below 100 fs (which can be related to the lifetime of 1*B*_*u*_^+^), and the rise time for the absorption signal of S_1_ (∼2*A*_*g*_^–^) was ∼600 fs.

Our extracted lifetimes for the S_2_ (1*B*_*u*_^+^) and S_*X*_ (1*B*_*u*_^+^) states of lutein, i.e., τ_2*x*_ = 22 fs and τ_*x*1_ = 132 fs, respectively, are also very close to the corresponding experimental times determined for other carotenoids with similar conjugation lengths.^9,10,12,14,15^ In particular, the involvement of an intermediate S_*X*_ state in S_2_ → S_1_ was already observed by Polli et al.^12^ for neurosporene (*N* = 9) and by Maiuri et al.^15^ for spheroidene (*N* = 10). For clarity, all of the aforementioned experimental relaxation times, together with the ones determined in the present work for lutein, are reported in Table S9 of the SI.

Our simulations finally showed that high-frequency BLA modes are fundamental in driving the transition to the 1*B*_*u*_^–^/S_*X*_ state. By analyzing the transient grating signal of β-carotene, Ghosh et al. have suggested that the S_*X*_ state is reached through a twist of one C=C double bond.^52^ We could not observe any excited-state torsion around any double C=C bond that could drive the excited-state relaxation, at least in the S_2_ → S_*X*_ → S_1_ ultrafast transition studied here. Importantly, with our atomistic surface-hopping dynamics we have not selected *a priori* the relevant coordinates, which are instead a result of the simulations.

Our results can finally help clarify the involvement of S_*X*_ in light harvesting and in energy transfer to chlorophylls.^16,19^ In particular, an S_*X*_ feature was observed by two-dimensional electronic spectroscopy in one of the lutein molecules bound to the major LHCII pigment–protein complex.^19^ By analyzing the amplitude oscillations at different points on the two-dimensional map, Son et al. revealed the nonadiabatic formation of a dark state, assigned to S_*X*_ of the lutein bound to site L2.^19^ This state is populated in less than 20 fs from S_2_ of the same lutein and transfers energy to the neighboring chlorophylls. Such a transfer forbids the observation of the S_*X*_ → S_1_ lifetime. However, the time scale of the nonadiabatic transition from S_2_ is again consistent with our results as well as with the measurements in solution. We therefore suggest that the S_*X*_ assignment to 1*B*_*u*_^–^ will also be relevant in light-harvesting complexes. Because the polarity of LHCII is intermediate between the gas phase and highly polar methanol, we expect similar excited-state dynamics to what we observed in our simulations. Steric constraints imposed by the protein may, however, influence the lutein excited states and their dynamics. Efforts are underway in our group to simulate the ultrafast dynamics of lutein in pigment–protein complexes.

In summary, the ultrafast decay pathway from bright state S_2_ to S_1_ has been simulated here for lutein in methanol solution using QM/MM nonadiabatic dynamics. These simulations have allowed the characterization of the nature of the intermediate state S_*X*_, subject of controversial discussions in the literature.^53^ The outcomes of our simulations indicate that the S_*X*_ state is formed through a change in nature of the S_2_ state, which switches from 1*B*_*u*_^+^ to 1*B*_*u*_^–^ character within ∼20 fs after the photoexcitation. Then, from the intermediate state S_*X*_ (1*B*_*u*_^–^) the system decays to S_1_ in about 130 fs. Unlike the S_2_ → S_*X*_ transition, which mainly occurs in the S_2_ potential energy surface, the S_*X*_ → S_1_ decay involves a transition between two different adiabatic PESs, namely, from the relaxed S_2_ state (∼1*B*_*u*_^–^) to S_1_. The whole relaxation pathway S_2_ → S_*X*_ → S_1_ is driven by the decrease in the bond-length alternation coordinate of the carotenoid conjugated chain.

## Methods

The electronic energies and wave functions of lutein were computed in a semiempirical AM1 framework using the floating occupation molecular orbital-configuration interaction (FOMO-CI) method.^47,48,54^ The CI was of the complete active space (CAS) type, with an active space of six electrons in six molecular orbitals (MOs). A Gaussian width for floating occupation of 0.1 hartree was used. The active MOs were of the π type, located on the polyene chain of lutein. The standard AM1 parameters were reoptimized in order to reproduce the best experimental and computational data available on excitation energies, oscillator strengths, and geometrical parameters of lutein. More details about the parametrization can be found in the Supporting Information (Section S1). In the simulations of lutein photodynamics, the interaction with the solvent (methanol) was taken into account using a QM/MM approach with electrostatic embedding. In particular, the lutein molecule was placed in a spherical cluster of 920 methanol molecules, which were all treated at the MM level using the OPLS-AA force field^55^ (Figure 1). The starting conditions for the surface hopping (SH) nonadiabatic simulations were sampled from a ground-state thermal trajectory at 300 K, performed using the Bussi–Parrinello stochastic thermostat.^56,57^ The QM/MM equilibration trajectory of lutein in methanol was run for 100 ps (Figures S3–S7 in Section S3.1). The last 80 ps of the thermalization were used to sample the initial conditions for the SH trajectories, which were selected by considering an excitation energy interval of 2.5 ± 0.1 eV and taking into account the radiative transition probability, according to the procedure outlined in ref (54). The SH calculations were performed using the local diabatization algorithm^48,58^ and with a time step of 0.1 fs, as employed in the integration of both the nuclear degrees of freedom and the electronic ones. Quantum decoherence was approximately taken into account with the energy-based decoherence correction (EDC) algorithm,^59^ setting the constant *C* to 0.1 hartree. A total of 200 SH trajectories (178 starting from S_2_ and 22 from S_3_) were propagated for 200 fs. The six low-lying singlet states were taken into account in the nonadiabatic dynamics. For each simulation time, the population of the *i*th adiabatic state was computed as the fraction of SH trajectories running on the *i*th adiabatic PES. To characterize the physical nature of the electronic states during the SH simulations, we applied a diabatization procedure previously devised in the framework of the FOMO-CI method.^60^ More details about the diabatization can be found in the Supporting Information (Section S2).

## Supporting Information Available

Parameterization of the semiempirical method; details of the diabatization; supplementary results for the QM/MM simulations in solution; reference states and orbitals used for diabatization; and SH simulations in vacuum (PDF)
Transparent Peer Review report available (PDF)
