# Supplementary material for: Ultrafast Excited-State Dynamics of Carotenoids and the Role of the SX State
Source: J Phys Chem Lett. 2022 Jul 19;13(29):6762–9. doi: 10.1021/acs.jpclett.2c01555 (PMC9340805; doi:10.1021/acs.jpclett.2c01555)
Supplement: Supplementary file 1 — jz2c01555_si_001.pdf [file jz2c01555_si_001.pdf]

Supporting Information: The Ultrafast Excited State Dynamics of Carotenoids and the Role of the S_X State

Davide Accomasso,[†,‡] Serra Arslancan,[†,‡] Lorenzo Cupellini,[†] Giovanni Granucci,[*,†] and Benedetta Mennucci[*,†]

†*Dipartimento di Chimica e Chimica Industriale, University of Pisa, via G. Moruzzi 13, 56124, Pisa, Italy*

‡*Contributed equally to this work*

E-mail: giovanni.granucci@unipi.it; benedetta.mennucci@unipi.it

S1 Parameterization of the semiempirical method

S1.1 Optimization of the AM1 semiempirical parameters

In our optimization of semiempirical parameters for lutein, we searched for the global minimum of the following function:

$$f(\mathbf{P}) = \sum_i W_i \left(\frac{X_i(\mathbf{P}) - Y_i}{RD_i} \right)^2 \quad \text{(S1)}$$

where for each i-th property included in the evaluation set, $X_i(\mathbf{P})$ is the semiempirical value computed using parameters $\mathbf{P}$, Y_i is the corresponding target value, W_i is the associated weight, and RD_i is a reference denominator included so as to have a relative error (usually $RD_i = Y_i$). To minimize $f(\mathbf{P})$ we used the simplex method[1], combined with a simulated annealing procedure[2]. In the set of target values $\{Y_i\}$, we included the vertical and adiabatic excitation energies of the S_1 and S_2 states, the S_0-S_1 energy gap at the S_1 minimum, the $S_0 \rightarrow S_1$ and $S_0 \rightarrow S_2$ oscillator strengths at the S_0 minimum, and selected geometrical parameters for the S_0 optimized geometry of lutein.

The target values for the vertical and adiabatic excitation energies of the S_1 and S_2 states were taken from the experimental work by Josue and Frank[3]. In particular, the target vertical and adiabatic excitation energies for S_2 correspond to the 0→1 and 0→0 transition energies reported at 293 K, respectively. The same data for the S_1 state are only reported at 77 K. In order to predict the 0→0 transition energy for S_1 at 293 K, we took the adiabatic ΔE(S_2-S_1) at 77 K as a reference. Hence, we substracted the adiabatic ΔE(S_2-S_1) at 77 K from the 0→0 ΔE(S_2-S_0) at 293 K, which gave us the predicted 0→0 ΔE(S_1-S_0) at 293 K. In the same manner, we took into account 0→0 and vertical ΔE(S_1-S_0) at 77 K from the fluorescence data to predict the vertical ΔE(S_1-S_0) at 293 K. The target values for the bond lengths and dihedral angles were taken from the S_0 minimum geometry of lutein, computed at the B3LYP/6-31G(d) level in vacuum.

In the parametrization, only the AM1 parameters of C atom were optimized, while for H and O atoms we maintained the standard AM1 parameters[4]. In order to deal with SCF convergence problems caused by orbital switching in and out of the active space, in the SCF calculation the

AM1 parameters β_S and β_P for O atoms were set equal to -15.0 eV, while the β_S parameter for H was set to -20.0 eV. The subsequent CI calculation was instead performed using the standard AM1 values of β_S and β_P for O and H (Table S2).

Additionally, to reproduce the DFT energy profile along the C-C dihedral angles of the polyene chain, potential terms were added to the state energies computed at the semiempirical FOMO-CASCI(6,6) level, as described below. The underestimation of the torsional barrier around single bonds appears to be a standard failure of NDDO semiempirical methods, already reported in literature[5].

In Table S1 we report the target data, the corresponding values computed with the FOMO-CASCI(6,6) method using the reoptimized AM1 parameters, and the associated weights employed in the reparameterization. The reoptimized AM1 semiempirical parameters of C atom, together with the standard AM1 parameters for H and O employed in our simulations, are given in Table S2.

Table S1: Results of the optimization of the semiempirical parameters for lutein. The target values, the corresponding semiempirical values, and the associated weights in the target function (Eq. S1) are reported. All energies are in eV. Bond lengths and dihedral angles are given in Å and degrees, respectively. For atom labels, see Figure S1

	Computed	Target	Weight
Excitation energies			
$\Delta E(S_1\text{-}S_0)$ (Adiabatic)	1.58	1.88 [3]	5.00
$\Delta E(S_2\text{-}S_0)$ (Adiabatic)	2.37	2.60 [3]	5.00
$\Delta E(S_1\text{-}S_0)$ at S_0min	2.19	2.00	5.00
$\Delta E(S_2\text{-}S_0)$ at S_0min	2.62	2.79 [3]	5.00
$\Delta E(S_1\text{-}S_0)$ at S_1min	0.98	1.68 [3]	5.00
Oscillator strengths			
$f(S_1\text{-}S_0)$ at S_0min	0.00	0.00	5.00
$f(S_2\text{-}S_0)$ at S_0min	2.87	2.11 [6]	5.00
Geometrical parameters (S_0 min.)			
C_2-C_3	1.42	1.47	1.00
C_3-C_4	1.32	1.36	1.00
C_4-C_5	1.42	1.45	1.00
C_5-C_6	1.36	1.37	1.00
C_6-C_7	1.38	1.43	1.00
C_7-C_8	1.34	1.37	1.00
C_8-C_9	1.41	1.44	1.00
C_9-C_{10}	1.36	1.37	1.00
C_{10}-C_{11}	1.38	1.43	1.00
C_{11}-C_{12}	1.35	1.37	1.00
C_{12}-C_{13}	1.38	1.43	1.00
C_{13}-C_{14}	1.36	1.37	1.00
C_{14}-C_{15}	1.41	1.44	1.00
C_{15}-C_{16}	1.34	1.36	1.00
C_{16}-C_{17}	1.39	1.44	1.00
C_{17}-C_{18}	1.36	1.37	1.00
C_{18}-C_{19}	1.41	1.46	1.00
C_{19}-C_{20}	1.32	1.35	1.00
C_{20}-C_{21}	1.45	1.51	1.00
$\angle C_1$-C_2-C_3-C_4	104.7	172.0	1.00
BLA	0.06	0.08	4.00

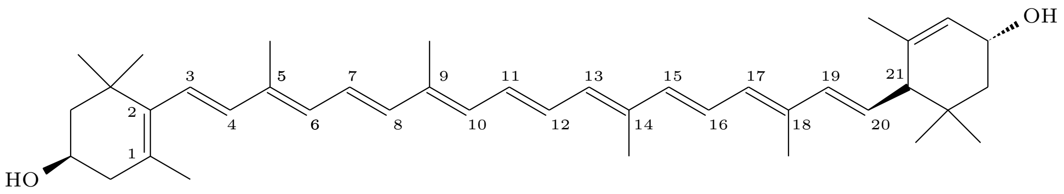

Figure S1: Molecular structure of lutein with labels for selected C atoms.

Table S2: Semiempirical parameters of AM1 Hamiltonian used in the dynamics simulations of lutein. Only the parameters of C atom were reoptimized, while for H and O atoms the standard AM1 parameters, here reported, were used.

Param	Units	C	O	H
U_{SS}	eV	-47.0918040777	-97.8300000	-11.3964270
U_{PP}	eV	-38.5817205839	-78.2623800	-
ζ_S	bohr^{-1}	1.4482025618	3.1080320	1.1880780
ζ_P	bohr^{-1}	1.5916740163	2.5240390	-
α	Å^{-1}	2.6902864404	4.4553710	2.8823240
β_S	eV	-21.0818298418	-29.2727730	-6.1737870
β_P	eV	-6.8630889568	-29.2727730	-
g_{SS}	eV	8.5531813229	15.4200000	12.8480000
g_{SP}	eV	11.6586347090	14.4800000	-
g_{PP}	eV	10.6703194165	14.5200000	-
g_{P2}	eV	9.6681110688	12.9800000	-
h_{SP}	eV	2.3147024774	3.9400000	-
K_1		0.0115858883	0.2809620	0.1227960
L_1	Å^{-1}	5.6943826119	5.0000000	5.0000000
M_1	Å	1.8761564812	0.8479180	1.2000000
K_2		0.0536123531	0.0814300	0.0050900
L_2	Å^{-1}	6.0084603043	7.0000000	5.0000000
M_2	Å	1.9736723662	1.4450710	1.8000000
K_3		-0.0188365285	-	-0.0183360
L_3	Å^{-1}	4.3695719734	-	2.0000000
M_3	Å	2.1613261146	-	2.1000000
K_4		-0.0012628900	-	-
L_4	Å^{-1}	4.9285766405	-	-
M_4	Å	2.7677199581	-	-

S1.2 Additional potentials for C-C dihedrals

To account for the energy barriers along the torsional coordinates around the C-C single bonds of the polyene chain of lutein, potential energy terms were added to the state energies computed at the reparametrized AM1/FOMO-CASCI(6,6) level. For each corrected dihedral angle ϕ, the functional form of the added potential term V is defined as follows:

$$V(\phi,\theta_1,\theta_2) = VT(\phi) * SW(\theta_1) * SW(\theta_2) \tag{S2}$$

$$VT(\phi) = \sum_{k=1}^{6} V_k \left[1 + cos(k\phi - \phi_k^{(0)})\right] \tag{S3}$$

$$SW(\theta_j) = 1 - 3 * X(\theta_j)^2 + 2 * X(\theta_j)^3, \quad j = 1,2 \tag{S4}$$

$$X(\theta_j) = \begin{cases} \frac{\theta_j - \theta_j^{(0)}}{\pi - \theta_j^{(0)}} & \text{if } \theta_j \geq \theta_j^{(0)} \\ 0 & \text{if } \theta_j < \theta_j^{(0)} \end{cases} \tag{S5}$$

In Eq. S2, the $SW(\theta_1)$ and $SW(\theta_2)$ factors allow to switch on and off the potential $VT(\phi)$ depending on the two angles θ_1=A-B-C and θ_2 =B-C-D (where A, B, C and D are the atoms defining the dihedral). In fact, $SW(\theta_j)$ (Eq. S4) is a cubic sigmoid function which goes smoothly from 1 to 0 as the angle θ_j increases from $\theta_j^{(0)}$ to π.

For each corrected dihedral, the parameters V_k, $\phi_k^{(0)}$ ($k = 1, \cdots, 6$) and $\theta_j^{(0)}$ ($j = 1, 2$) were determined so as to reproduce as much as possible the B3LYP/6-31G(d) potential energy curve obtained by performing a relaxed scan along the dihedral. The parameter values defining the additional potential terms employed in our simulations are reported in Table S3. As an example, in Figure S2 we show the potential energy curves along the C_9-C_{10}-C_{11}-C_{12} dihedral of lutein (Figure S1) computed at the B3LYP/6-31G(d) level and using our reparametrized AM1/FOMO-CASCI(6,6) method, with and without the additional potential terms.

Table S3: Parameter values defining the additional potential energy terms (Eq. S2) employed in our simulations of lutein. V_2 and V_3 are reported in eV, while $\phi_2^{(0)}$, $\phi_3^{(0)}$, $\theta_1^{(0)}$ and $\theta_2^{(0)}$ are in degrees. In our simulations, V_1, V_4, V_5 and V_6 (Eq. S3) are set to zero. For atom labels, see Figure S1.

Dihedral	V_2	$\phi_2^{(0)}$	V_3	$\phi_3^{(0)}$	$\theta_1^{(0)}$	$\theta_2^{(0)}$
C_3-C_4-C_5-C_6	0.15	180.0	0.070	0.0	150.0	150.0
C_5-C_6-C_7-C_8	0.22	180.0	0.050	0.0	150.0	150.0
C_7-C_8-C_9-C_{10}	0.20	180.0	0.050	0.0	150.0	150.0
C_9-C_{10}-C_{11}-C_{12}	0.20	180.0	0.060	0.0	150.0	150.0
C_{11}-C_{12}-C_{13}-C_{14}	0.60	180.0	0.060	0.0	150.0	150.0
C_{13}-C_{14}-C_{15}-C_{16}	0.20	180.0	0.055	0.0	150.0	150.0
C_{15}-C_{16}-C_{17}-C_{18}	0.40	180.0	0.055	0.0	150.0	150.0
C_{17}-C_{18}-C_{19}-C_{20}	0.40	180.0	0.050	0.0	150.0	150.0

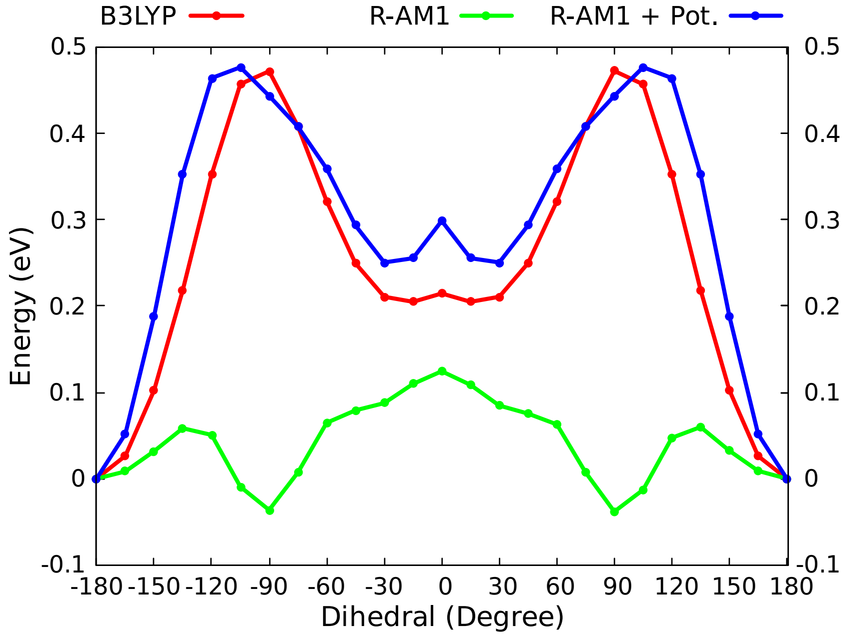

Figure S2: Ground state potential energy curves obtained from the relaxed scan along the C_9-C_{10}-C_{11}-C_{12} dihedral of lutein (Figure S1), computed at the B3LYP/6-31G(d) level and using the reparametrized AM1/FOMO-CASCI(6,6) method, with and without the additional potential terms (indicated as R-AM1 + Pot., and R-AM1, respectively).

S2 Diabatization

To characterize the physical nature of the electronic states during the SH simulations, we applied a diabatization procedure previously devised in the framework of the FOMO-CI method[7].

Along each Surface Hopping trajectory, both the active MOs and the adiabatic CI states were transformed so as they maximize the overlap with previously chosen references. The latter are the active MOs and low-lying adiabatic states of lutein computed at its S_0 minimum geometry (see Tables S10 and S11). In particular, the active MOs were transformed by maximize the overlaps with the reference MOs, and the CI coefficients of the adiabatic states in the basis of the Slater determinants built on the new MOs were computed. Then, a set of quasi-diabatic states (hereafter indicated as diabatic states) is defined by applying a unitary transformation to the adiabatic states. As in the case of the orbital rotation, such transformation is determined by maximizing the overlaps between the diabatic wavefunctions and the reference states. The population of a diabatic state $\Psi_i^{(D)}$ was computed as its square coefficient in the adiabatic state $\Psi_k^{(A)}$ on which the trajectory is running at that time, i.e. $|\langle\Psi_i^{(X)}|\Psi_k^{(A)}\rangle|^2$. The fluxes of population between states were analyzed by considering that a transition from diabatic state i to j has occurred if, along a single trajectory, the diabatic state with the largest weight (i.e. square coefficient) in the current adiabatic wavefunction has changed from i to j. The same assumption was used to compute average transition rates between pairs of diabatic states. In our diabatization analysis, 4 diabatic states were defined by making use of 6 reference active MOs and 4 reference states.

The (pseudo-)symmetry labels $1A_g^-$, $2A_g^-$, $1B_u^+$ and $1B_u^-$ were assigned to the reference states (and to the corresponding diabatic states) by comparison with the adiabatic states of the all-trans linear polyene with 10 double bonds, $C_{20}H_{22}$ (see Table 1 of the main text).

S3 Supplementary results

S3.1 Ground state thermal equilibration

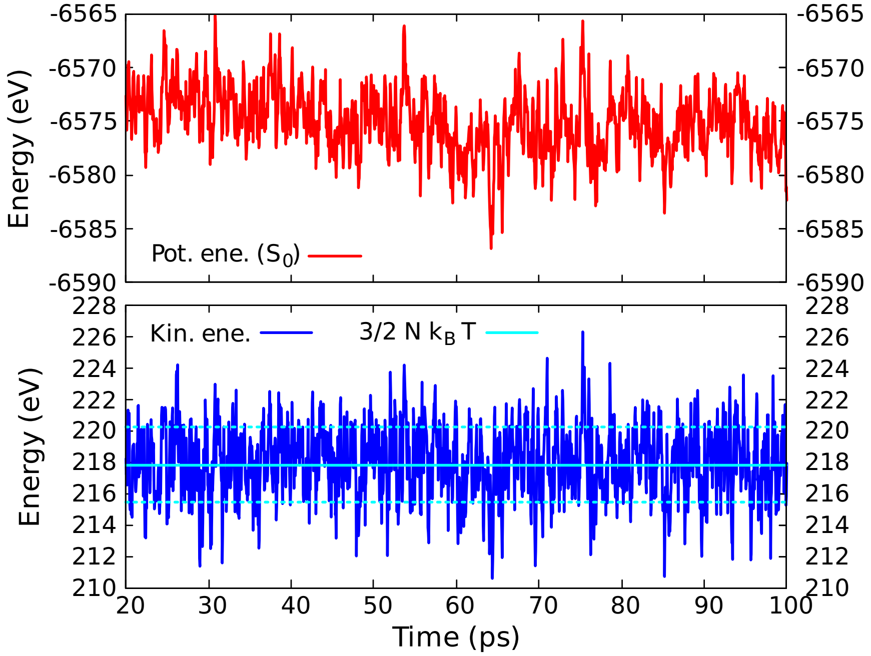

Figure S3: Potential and kinetic energies (in eV) of the whole QM/MM system as functions of time obtained from the ground state thermal equilibration of lutein in methanol solution. Each point in the plot is obtained by averaging over a time interval of 50 fs.

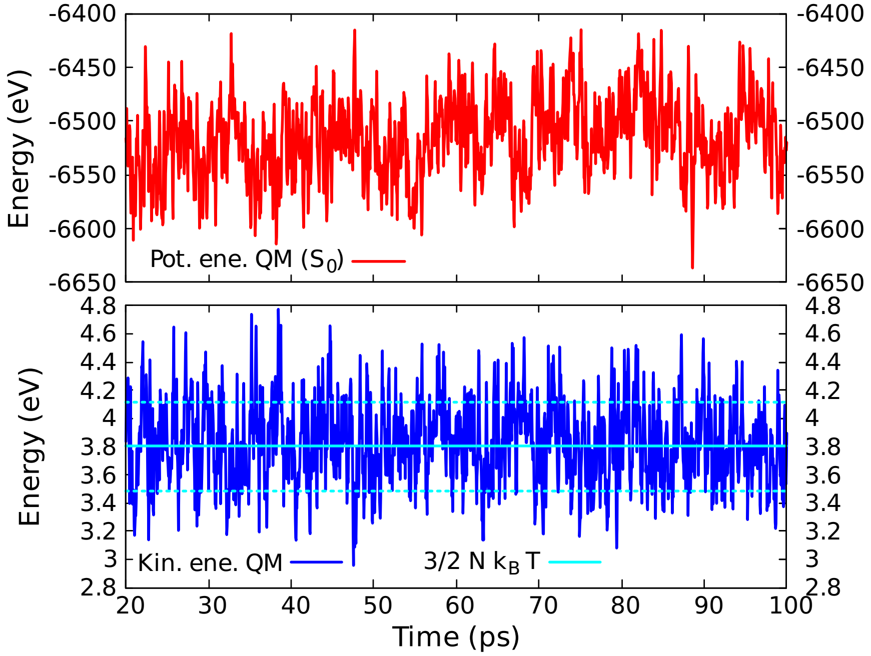

Figure S4: Potential and kinetic energies (in eV) of the QM subsystem as functions of time obtained from the ground state thermal equilibration of lutein in methanol solution. Each point in the plot is obtained by averaging over a time interval of 50 fs.

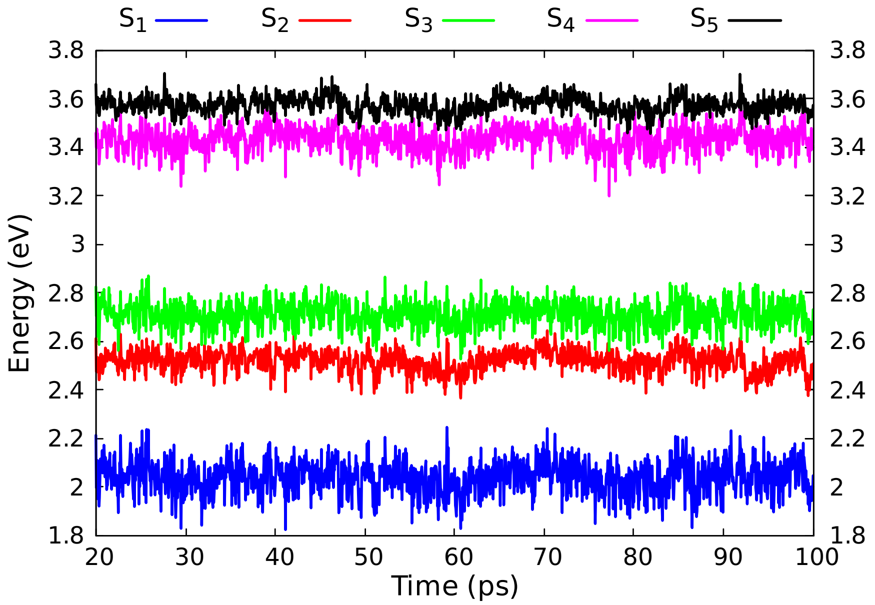

Figure S5: Adiabatic excitation energies (in eV) from the ground state (S_0) to the five low-lying excited states (S_1-S_5) as functions of time obtained from the ground state thermal equilibration of lutein in methanol solution. Each point in the plot is obtained by averaging over a time interval of 50 fs.

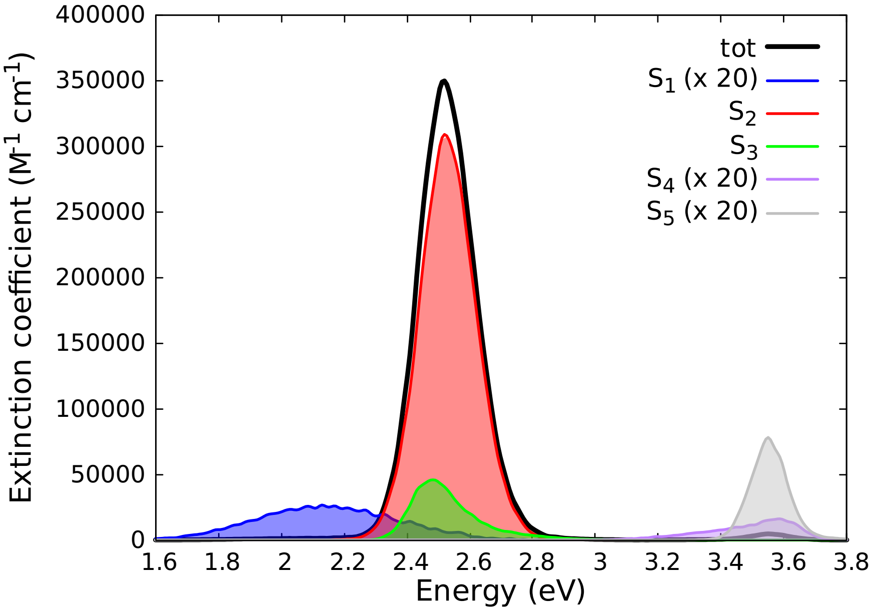

Figure S6: Absorption spectrum obtained from the ground state thermal equilibration of lutein in methanol solution. The spectrum is obtained by averaging over the last 80 ps of the thermal equilibration (time interval: 20-100 ps).

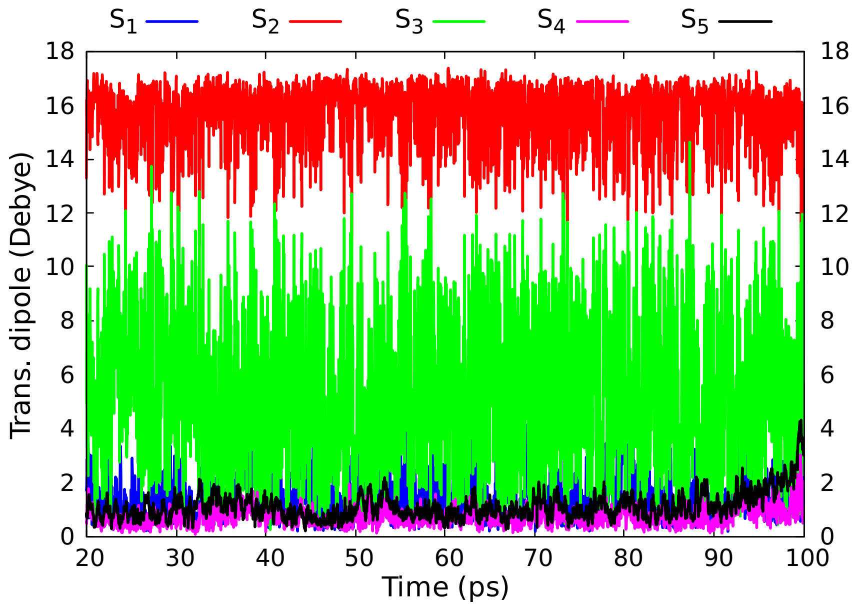

Figure S7: Transition dipoles (modulus in Debye) from the ground state (S_0) to the five low-lying adiabatic excited states (S_1-S_5) as functions of time obtained from the ground state thermal equilibration of lutein in methanol solution. Each point in the plot is obtained by averaging over a time interval of 25 fs.

S3.2 Excited state dynamics

Table S4: Comparison of the energy gaps, electronic couplings and BLA values at $t = 0$ and at the transitions between pairs of states ($i \to j$), averaged over all SH trajectories. The average times (in fs) for the selected geometries (X) are also given. The energy gaps and electronic couplings are reported in units of meV, while the BLA values are in Å. For the electronic couplings, the mean absolute values are reported.

States		Geom.	Energy gap	coupling	BLA	Time
$1B_u^+$	$1B_u^-$	X_0	152.0	27.6	0.0491	0
		$X_{i\to j}$	-15.2	30.8	0.0240	28
$1B_u^-$	$1B_u^+$	X_0	-152.0	27.6	0.0491	0
		$X_{i\to j}$	0.8	23.8	0.0268	34
$1B_u^-$	$2A_g^-$	X_0	-647.9	61.4	0.0491	0
		$X_{i\to j}$	-642.7	98.3	0.0020	112
$1B_u^-$	$1A_g^-$	X_0	-2634.5	83.6	0.0491	0
		$X_{i\to j}$	-730.0	87.6	-0.0078	132

Table S5: Average transition rates between pairs of states in both the adiabatic basis and the diabatic one obtained in the simulations of the excited state dynamics for lutein in methanol solution. Each rate, defined as $\frac{\#\ transitions}{\#\ trajectories\ \cdot\ time\ interval}$, is computed over the whole simulation time interval (0.2 ps) and reported in units of ps^{-1}.

States		Rates (ps^{-1})		
i	j	$i \to j$	$j \to i$	net[a]
Adiabatic basis				
S_1	S_0	0.400	0.000	0.400
S_2	S_0	0.200	0.000	0.200
S_3	S_0	0.000	0.000	0.000
S_2	S_1	5.300	0.950	4.350
S_3	S_1	0.000	0.000	0.000
S_3	S_2	3.375	2.825	0.550
Diabatic basis				
$2^1A_g^-$	$1^1A_g^-$	2.313	2.139	0.174
$1^1B_u^+$	$1^1A_g^-$	0.000	0.000	0.000
$1^1B_u^-$	$1^1A_g^-$	1.791	1.368	0.423
$1^1B_u^+$	$2^1A_g^-$	0.075	0.000	0.075
$1^1B_u^-$	$2^1A_g^-$	5.597	2.761	2.836
$1^1B_u^-$	$1^1B_u^+$	4.826	9.502	-4.676

[a] Difference between the $i \to j$ rate and the $j \to i$ one.

Table S6: Average electronic Hamiltonian matrices in the diabatic basis at the starting geometries ($t = 0$) and at the geometries where S_1 is the active adiabatic state (S_1 geometries), obtained from the simulations of the excited state dynamics for lutein in methanol solution. All matrix elements are reported in units of meV. For the off-diagonal elements, the mean absolute values are reported.

Starting geometries

$\hat{\mathcal{H}}_{el}$	$1^1A_g^-$	$2^1A_g^-$	$1^1B_u^+$	$1^1B_u^-$
$1^1A_g^-$	0.00	85.36	104.07	83.58
$2^1A_g^-$	85.36	1986.59	30.73	61.43
$1^1B_u^+$	104.07	30.73	2482.50	27.64
$1^1B_u^-$	83.58	61.43	27.64	2634.46

S_1 geometries

$\hat{\mathcal{H}}_{el}$	$1^1A_g^-$	$2^1A_g^-$	$1^1B_u^+$	$1^1B_u^-$
$1^1A_g^-$	0.00	551.01	152.77	141.89
$2^1A_g^-$	551.01	521.03	94.73	126.29
$1^1B_u^+$	152.77	94.73	2051.80	56.98
$1^1B_u^-$	141.89	126.29	56.98	1358.44

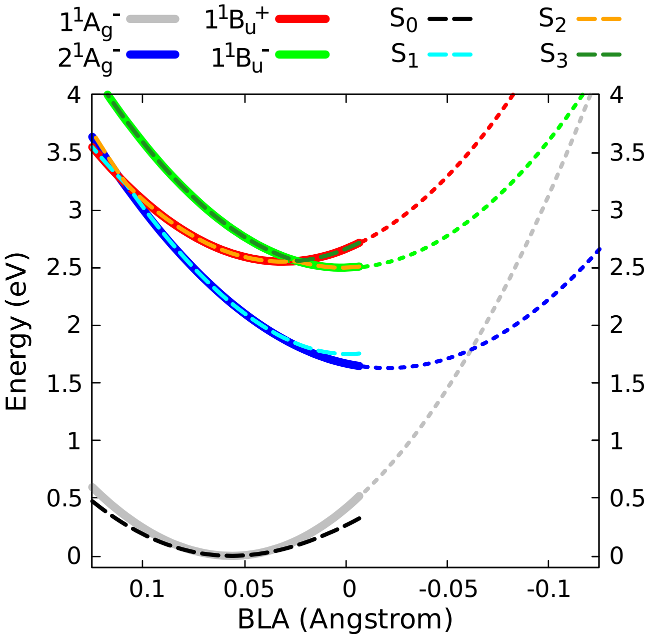

Figure S8: Energies (eV) of the four low-lying adiabatic and diabatic states as functions of BLA (Å) obtained from a relaxed scan on the S_0 state of lutein in vacuum, using our reparametrized AM1/FOMO-CASCI(6,6) method. The fitting functions (parabolas) of the diabatic energies are also shown (dashed thin lines).

Table S7: Complete list of relaxation pathways to the first excited state in the adiabatic basis (i.e. S_1) obtained in the surface hopping simulations of lutein (QM) in methanol solution (MM). For each identified pathway, the total number of surface hopping trajectories and the corresponding percentage are also reported.

Pathway	# Traj.	%
$S_2 \rightarrow S_1$	70	35.0
$S_2 \rightarrow S_3 \rightarrow S_2 \rightarrow S_1$	31	15.5
$S_2 \rightarrow S_3 \rightarrow S_2 \rightarrow S_3 \rightarrow S_2 \rightarrow S_1$	14	7.0
$S_3 \rightarrow S_2 \rightarrow S_1$	12	6.0
$S_2 \rightarrow S_1 \rightarrow S_0$	11	5.5
$S_2 \rightarrow S_1 \rightarrow S_2 \rightarrow S_1$	10	5.0
S_2	7	3.5
$S_2 \rightarrow S_0$	6	3.0
$S_2 \rightarrow S_1 \rightarrow S_2 \rightarrow S_1 \rightarrow S_2 \rightarrow S_1$	4	2.0
$S_2 \rightarrow S_3 \rightarrow S_2$	4	2.0
$S_2 \rightarrow S_3 \rightarrow S_2 \rightarrow S_1 \rightarrow S_0$	3	1.5
$S_2 \rightarrow S_3 \rightarrow S_2 \rightarrow S_3 \rightarrow S_2 \rightarrow S_3 \rightarrow S_2$	2	1.0
$S_2 \rightarrow S_3 \rightarrow S_2 \rightarrow S_3 \rightarrow S_2 \rightarrow S_1 \rightarrow S_2 \rightarrow S_1$	2	1.0
$S_2 \rightarrow S_3 \rightarrow S_2 \rightarrow S_3 \rightarrow S_2 \rightarrow S_3 \rightarrow S_2 \rightarrow S_1$	2	1.0
$S_2 \rightarrow S_3 \rightarrow S_2 \rightarrow S_1 \rightarrow S_2 \rightarrow S_1$	2	1.0
$S_3 \rightarrow S_2 \rightarrow S_1 \rightarrow S_2 \rightarrow S_1$	2	1.0
$S_2 \rightarrow S_3 \rightarrow S_2 \rightarrow S_1 \rightarrow S_2 \rightarrow S_1 \rightarrow S_2 \rightarrow S_1$	2	1.0
$S_3 \rightarrow S_2$	2	1.0
$S_3 \rightarrow S_2 \rightarrow S_0$	2	1.0
$S_2 \rightarrow S_3 \rightarrow S_2 \rightarrow S_3 \rightarrow S_2 \rightarrow S_3 \rightarrow S_2 \rightarrow S_3 \rightarrow S_2$	1	0.5
$S_2 \rightarrow S_3 \rightarrow S_2 \rightarrow S_3 \rightarrow S_2 \rightarrow S_3 \rightarrow S_2 \rightarrow S_3 \rightarrow S_2 \rightarrow S_1$	1	0.5
$S_2 \rightarrow S_3 \rightarrow S_2 \rightarrow S_3 \rightarrow S_2 \rightarrow S_3 \rightarrow S_2 \rightarrow S_1 \rightarrow S_0$	1	0.5
$S_2 \rightarrow S_3 \rightarrow S_2 \rightarrow S_3 \rightarrow S_2$	1	0.5
$S_2 \rightarrow S_3 \rightarrow S_2 \rightarrow S_3 \rightarrow S_2 \rightarrow S_1 \rightarrow S_2 \rightarrow S_1 \rightarrow S_2 \rightarrow S_1$	1	0.5
$S_2 \rightarrow S_3 \rightarrow S_2 \rightarrow S_3 \rightarrow S_2 \rightarrow S_3 \rightarrow S_2 \rightarrow S_1 \rightarrow S_2 \rightarrow S_1$	1	0.5
$S_2 \rightarrow S_3 \rightarrow S_2 \rightarrow S_3 \rightarrow S_2 \rightarrow S_3 \rightarrow S_2 \rightarrow S_3 \rightarrow S_2 \rightarrow S_3 \rightarrow S_2 \rightarrow S_3 \rightarrow S_2 \rightarrow S_3 \rightarrow S_2$	1	0.5
$S_3 \rightarrow S_2 \rightarrow S_3 \rightarrow S_2 \rightarrow S_1 \rightarrow S_2 \rightarrow S_1$	1	0.5
$S_2 \rightarrow S_1 \rightarrow S_2 \rightarrow S_1 \rightarrow S_2 \rightarrow S_1 \rightarrow S_2 \rightarrow S_1 \rightarrow S_2 \rightarrow S_1$	1	0.5
$S_3 \rightarrow S_2 \rightarrow S_1 \rightarrow S_2 \rightarrow S_1 \rightarrow S_2 \rightarrow S_1$	1	0.5
$S_3 \rightarrow S_2 \rightarrow S_3 \rightarrow S_2 \rightarrow S_1$	1	0.5
$S_3 \rightarrow S_2 \rightarrow S_1 \rightarrow S_0$	1	0.5

Table S8: Complete list of relaxation pathways in the diabatic basis obtained in the surface hopping simulations of lutein in methanol solution. The pathways towards the $2^1A_g^-$ and $1^1A_g^-$ states are grouped together. For each identified pathway, the total number of surface hopping trajectories and the corresponding percentage are also reported.

Pathway	# Traj.	%
$1^1B_u^+ \rightarrow 1^1B_u^- \rightarrow 2^1A_g^-{+}1^1A_g^-$	58	29.0
$1^1B_u^+ \rightarrow 1^1B_u^- \rightarrow 1^1B_u^+ \rightarrow 1^1B_u^- \rightarrow 2^1A_g^-{+}1^1A_g^-$	27	13.5
$1^1B_u^+ \rightarrow 1^1B_u^- \rightarrow 1^1B_u^+ \rightarrow 1^1B_u^- \rightarrow 1^1B_u^+ \rightarrow$		
$\rightarrow 1^1B_u^- \rightarrow 2^1A_g^-{+}1^1A_g^-$	14	7.0
$1^1B_u^+ \rightarrow 1^1B_u^-$	6	3.0
$1^1B_u^+ \rightarrow 1^1B_u^- \rightarrow 2^1A_g^-{+}1^1A_g^- \rightarrow 1^1B_u^- \rightarrow 2^1A_g^-{+}1^1A_g^- \rightarrow$		
$\rightarrow 1^1B_u^- \rightarrow 2^1A_g^-{+}1^1A_g^- \rightarrow 1^1B_u^- \rightarrow 2^1A_g^-{+}1^1A_g^-$	6	3.0
$1^1B_u^+ \rightarrow 1^1B_u^- \rightarrow 2^1A_g^-{+}1^1A_g^- \rightarrow 1^1B_u^- \rightarrow 2^1A_g^-{+}1^1A_g^-$	6	3.0
$1^1B_u^+ \rightarrow 1^1B_u^- \rightarrow 1^1B_u^+ \rightarrow 1^1B_u^- \rightarrow 1^1B_u^+ \rightarrow$		
$\rightarrow 1^1B_u^- \rightarrow 1^1B_u^+ \rightarrow 1^1B_u^- \rightarrow 2^1A_g^-{+}1^1A_g^-$	5	2.5
$1^1B_u^+ \rightarrow 1^1B_u^- \rightarrow 2^1A_g^-{+}1^1A_g^- \rightarrow 1^1B_u^- \rightarrow 2^1A_g^-{+}1^1A_g^- \rightarrow$		
$\rightarrow 1^1B_u^- \rightarrow 2^1A_g^-{+}1^1A_g^-$	5	2.5
$1^1B_u^+ \rightarrow 1^1B_u^- \rightarrow 1^1B_u^+ \rightarrow 1^1B_u^- \rightarrow 2^1A_g^-{+}1^1A_g^- \rightarrow$		
$\rightarrow 1^1B_u^- \rightarrow 2^1A_g^-{+}1^1A_g^-$	4	2.0
$1^1B_u^+ \rightarrow 1^1B_u^- \rightarrow 2^1A_g^-{+}1^1A_g^- \rightarrow 1^1B_u^- \rightarrow 2^1A_g^-{+}1^1A_g^- \rightarrow$		
$\rightarrow 1^1B_u^- \rightarrow 2^1A_g^-{+}1^1A_g^- \rightarrow 1^1B_u^- \rightarrow 2^1A_g^-{+}1^1A_g^- \rightarrow$		
$\rightarrow 1^1B_u^- \rightarrow 2^1A_g^-{+}1^1A_g^-$	4	2.0
$1^1B_u^+ \rightarrow 1^1B_u^- \rightarrow 1^1B_u^+ \rightarrow 1^1B_u^- \rightarrow 2^1A_g^-{+}1^1A_g^- \rightarrow$		
$\rightarrow 1^1B_u^- \rightarrow 2^1A_g^-{+}1^1A_g^- \rightarrow 1^1B_u^- \rightarrow 2^1A_g^-{+}1^1A_g^-$	4	2.0
$1^1B_u^+ \rightarrow 1^1B_u^- \rightarrow 1^1B_u^+ \rightarrow 1^1B_u^-$	3	1.5
$1^1B_u^+ \rightarrow 1^1B_u^- \rightarrow 1^1B_u^+ \rightarrow 1^1B_u^- \rightarrow 2^1A_g^-{+}1^1A_g^- \rightarrow$		
$\rightarrow 1^1B_u^- \rightarrow 2^1A_g^-{+}1^1A_g^- \rightarrow 1^1B_u^- \rightarrow 2^1A_g^-{+}1^1A_g^- \rightarrow$		
$\rightarrow 1^1B_u^- \rightarrow 2^1A_g^-{+}1^1A_g^-$	3	1.5

$1^1B_u^+ \rightarrow 1^1B_u^- \rightarrow 1^1B_u^+ \rightarrow 1^1B_u^- \rightarrow 1^1B_u^+ \rightarrow$		
$\rightarrow 1^1B_u^- \rightarrow 1^1B_u^+ \rightarrow 1^1B_u^- \rightarrow 1^1B_u^+ \rightarrow 1^1B_u^-$	3	1.5
$1^1B_u^+ \rightarrow 1^1B_u^- \rightarrow 1^1B_u^+ \rightarrow 1^1B_u^- \rightarrow 1^1B_u^+ \rightarrow$		
$\rightarrow 1^1B_u^- \rightarrow 2^1A_g^-{+}1^1A_g^- \rightarrow 1^1B_u^-$	3	1.5
$1^1B_u^- \rightarrow 2^1A_g^-{+}1^1A_g^-$	3	1.5
$1^1B_u^+ \rightarrow 1^1B_u^- \rightarrow 1^1B_u^+ \rightarrow 1^1B_u^- \rightarrow 1^1B_u^+ \rightarrow$		
$\rightarrow 1^1B_u^- \rightarrow 1^1B_u^+ \rightarrow 1^1B_u^-$	3	1.5
$1^1B_u^+ \rightarrow 1^1B_u^- \rightarrow 2^1A_g^-{+}1^1A_g^- \rightarrow 1^1B_u^- \rightarrow 2^1A_g^-{+}1^1A_g^- \rightarrow$		
$\rightarrow 1^1B_u^- \rightarrow 2^1A_g^-{+}1^1A_g^- \rightarrow 1^1B_u^- \rightarrow 2^1A_g^-{+}1^1A_g^- \rightarrow$		
$\rightarrow 1^1B_u^- \rightarrow 2^1A_g^-{+}1^1A_g^- \rightarrow 1^1B_u^- \rightarrow 2^1A_g^-{+}1^1A_g^- \rightarrow 1^1B_u^- \rightarrow$		
$\rightarrow 2^1A_g^-{+}1^1A_g^- \rightarrow 1^1B_u^- \rightarrow 2^1A_g^-{+}1^1A_g^-$	2	1.0
$1^1B_u^+ \rightarrow 1^1B_u^- \rightarrow 1^1B_u^+ \rightarrow 1^1B_u^- \rightarrow 1^1B_u^+ \rightarrow$		
$\rightarrow 1^1B_u^- \rightarrow 1^1B_u^+ \rightarrow 1^1B_u^- \rightarrow 1^1B_u^+ \rightarrow$		
$\rightarrow 1^1B_u^- \rightarrow 2^1A_g^-{+}1^1A_g^-$	2	1.0

Table S8: (continued from previous page)

Pathway	# Traj.	%
$1^1\mathrm{B}_u^+ \rightarrow 1^1\mathrm{B}_u^- \rightarrow 2^1\mathrm{A}_g^-{+}1^1\mathrm{A}_g^- \rightarrow 1^1\mathrm{B}_u^- \rightarrow 2^1\mathrm{A}_g^-{+}1^1\mathrm{A}_g^- \rightarrow$<br>$\rightarrow 1^1\mathrm{B}_u^- \rightarrow 2^1\mathrm{A}_g^-{+}1^1\mathrm{A}_g^- \rightarrow 1^1\mathrm{B}_u^-$	2	1.0
$1^1\mathrm{B}_u^- \rightarrow 1^1\mathrm{B}_u^+ \rightarrow 1^1\mathrm{B}_u^- \rightarrow 2^1\mathrm{A}_g^-{+}1^1\mathrm{A}_g^-$	2	1.0
$1^1\mathrm{B}_u^+ \rightarrow 1^1\mathrm{B}_u^- \rightarrow 2^1\mathrm{A}_g^-{+}1^1\mathrm{A}_g^- \rightarrow 1^1\mathrm{B}_u^-$	2	1.0
$1^1\mathrm{B}_u^+ \rightarrow 1^1\mathrm{B}_u^- \rightarrow 2^1\mathrm{A}_g^-{+}1^1\mathrm{A}_g^- \rightarrow 1^1\mathrm{B}_u^- \rightarrow 2^1\mathrm{A}_g^-{+}1^1\mathrm{A}_g^- \rightarrow$<br>$\rightarrow 1^1\mathrm{B}_u^-$	2	1.0
$1^1\mathrm{B}_u^- \rightarrow 2^1\mathrm{A}_g^-{+}1^1\mathrm{A}_g^- \rightarrow 1^1\mathrm{B}_u^- \rightarrow 2^1\mathrm{A}_g^-{+}1^1\mathrm{A}_g^-$	2	1.0
$1^1\mathrm{B}_u^+ \rightarrow 1^1\mathrm{B}_u^- \rightarrow 1^1\mathrm{B}_u^+ \rightarrow 1^1\mathrm{B}_u^- \rightarrow 1^1\mathrm{B}_u^+ \rightarrow$<br>$\rightarrow 1^1\mathrm{B}_u^- \rightarrow 2^1\mathrm{A}_g^-{+}1^1\mathrm{A}_g^- \rightarrow 1^1\mathrm{B}_u^- \rightarrow 2^1\mathrm{A}_g^-{+}1^1\mathrm{A}_g^- \rightarrow$<br>$\rightarrow 1^1\mathrm{B}_u^- \rightarrow 2^1\mathrm{A}_g^-{+}1^1\mathrm{A}_g^-$	2	1.0
$1^1\mathrm{B}_u^+ \rightarrow 2^1\mathrm{A}_g^-{+}1^1\mathrm{A}_g^-$	2	1.0
$1^1\mathrm{B}_u^+ \rightarrow 1^1\mathrm{B}_u^- \rightarrow 1^1\mathrm{B}_u^+ \rightarrow 1^1\mathrm{B}_u^- \rightarrow 1^1\mathrm{B}_u^+ \rightarrow$<br>$\rightarrow 1^1\mathrm{B}_u^- \rightarrow 1^1\mathrm{B}_u^+ \rightarrow 1^1\mathrm{B}_u^- \rightarrow 1^1\mathrm{B}_u^+ \rightarrow$<br>$\rightarrow 1^1\mathrm{B}_u^- \rightarrow 1^1\mathrm{B}_u^+ \rightarrow 1^1\mathrm{B}_u^- \rightarrow 2^1\mathrm{A}_g^-{+}1^1\mathrm{A}_g^-$	2	1.0
$1^1\mathrm{B}_u^+ \rightarrow 1^1\mathrm{B}_u^- \rightarrow 1^1\mathrm{B}_u^+ \rightarrow 1^1\mathrm{B}_u^- \rightarrow 2^1\mathrm{A}_g^-{+}1^1\mathrm{A}_g^- \rightarrow$<br>$\rightarrow 1^1\mathrm{B}_u^- \rightarrow 2^1\mathrm{A}_g^-{+}1^1\mathrm{A}_g^- \rightarrow 1^1\mathrm{B}_u^- \rightarrow 2^1\mathrm{A}_g^-{+}1^1\mathrm{A}_g^- \rightarrow 1^1\mathrm{B}_u^-$	2	1.0
$1^1\mathrm{B}_u^+ \rightarrow 1^1\mathrm{B}_u^- \rightarrow 2^1\mathrm{A}_g^-{+}1^1\mathrm{A}_g^- \rightarrow 1^1\mathrm{B}_u^- \rightarrow 2^1\mathrm{A}_g^-{+}1^1\mathrm{A}_g^- \rightarrow$<br>$\rightarrow 1^1\mathrm{B}_u^- \rightarrow 2^1\mathrm{A}_g^-{+}1^1\mathrm{A}_g^- \rightarrow 1^1\mathrm{B}_u^- \rightarrow 2^1\mathrm{A}_g^-{+}1^1\mathrm{A}_g^- \rightarrow$<br>$\rightarrow 1^1\mathrm{B}_u^- \rightarrow 2^1\mathrm{A}_g^-{+}1^1\mathrm{A}_g^- \rightarrow 1^1\mathrm{B}_u^- \rightarrow 2^1\mathrm{A}_g^-{+}1^1\mathrm{A}_g^-$	1	0.5
$1^1\mathrm{B}_u^- \rightarrow 1^1\mathrm{B}_u^+ \rightarrow 1^1\mathrm{B}_u^- \rightarrow 1^1\mathrm{B}_u^+ \rightarrow 1^1\mathrm{B}_u^- \rightarrow$<br>$\rightarrow 1^1\mathrm{B}_u^+ \rightarrow 1^1\mathrm{B}_u^- \rightarrow 1^1\mathrm{B}_u^+ \rightarrow 1^1\mathrm{B}_u^- \rightarrow 2^1\mathrm{A}_g^-{+}1^1\mathrm{A}_g^-$	1	0.5
$1^1\mathrm{B}_u^+ \rightarrow 1^1\mathrm{B}_u^- \rightarrow 1^1\mathrm{B}_u^+ \rightarrow 1^1\mathrm{B}_u^- \rightarrow 1^1\mathrm{B}_u^+ \rightarrow$<br>$\rightarrow 1^1\mathrm{B}_u^-$	1	0.5
$1^1\mathrm{B}_u^+ \rightarrow 1^1\mathrm{B}_u^- \rightarrow 1^1\mathrm{B}_u^+ \rightarrow 1^1\mathrm{B}_u^- \rightarrow 1^1\mathrm{B}_u^+ \rightarrow$		

$\rightarrow 1^1\mathrm{B}_u^- \rightarrow 1^1\mathrm{B}_u^+ \rightarrow 1^1\mathrm{B}_u^- \rightarrow 2^1\mathrm{A}_g^-{+}1^1\mathrm{A}_g^- \rightarrow$		
$\rightarrow 1^1\mathrm{B}_u^- \rightarrow 2^1\mathrm{A}_g^-{+}1^1\mathrm{A}_g^- \rightarrow 1^1\mathrm{B}_u^-$	1	0.5
$1^1\mathrm{B}_u^+ \rightarrow 1^1\mathrm{B}_u^- \rightarrow 1^1\mathrm{B}_u^+ \rightarrow 1^1\mathrm{B}_u^- \rightarrow 1^1\mathrm{B}_u^+ \rightarrow$		
$\rightarrow 1^1\mathrm{B}_u^- \rightarrow 1^1\mathrm{B}_u^+ \rightarrow 1^1\mathrm{B}_u^- \rightarrow 1^1\mathrm{B}_u^+ \rightarrow$		
$\rightarrow 1^1\mathrm{B}_u^- \rightarrow 1^1\mathrm{B}_u^+ \rightarrow 1^1\mathrm{B}_u^- \rightarrow 1^1\mathrm{B}_u^+ \rightarrow 1^1\mathrm{B}_u^- \rightarrow$		
$\rightarrow 1^1\mathrm{B}_u^+ \rightarrow 1^1\mathrm{B}_u^- \rightarrow 1^1\mathrm{B}_u^+ \rightarrow 1^1\mathrm{B}_u^- \rightarrow$		
$\rightarrow 1^1\mathrm{B}_u^+ \rightarrow 1^1\mathrm{B}_u^-$	1	0.5
$1^1\mathrm{B}_u^+ \rightarrow 1^1\mathrm{B}_u^- \rightarrow 2^1\mathrm{A}_g^-{+}1^1\mathrm{A}_g^- \rightarrow 1^1\mathrm{B}_u^- \rightarrow 2^1\mathrm{A}_g^-{+}1^1\mathrm{A}_g^- \rightarrow$		
$\rightarrow 1^1\mathrm{B}_u^- \rightarrow 2^1\mathrm{A}_g^-{+}1^1\mathrm{A}_g^- \rightarrow 1^1\mathrm{B}_u^- \rightarrow 2^1\mathrm{A}_g^-{+}1^1\mathrm{A}_g^- \rightarrow$		
$\rightarrow 1^1\mathrm{B}_u^- \rightarrow 2^1\mathrm{A}_g^-{+}1^1\mathrm{A}_g^- \rightarrow 1^1\mathrm{B}_u^- \rightarrow 2^1\mathrm{A}_g^-{+}1^1\mathrm{A}_g^- \rightarrow 1^1\mathrm{B}_u^- \rightarrow$		
$\rightarrow 2^1\mathrm{A}_g^-{+}1^1\mathrm{A}_g^-$	1	0.5

Table S8: (continued from previous page)

Pathway	# Traj.	%
$1^1\mathrm{B}_u^+ \rightarrow 1^1\mathrm{B}_u^- \rightarrow 1^1\mathrm{B}_u^+ \rightarrow 1^1\mathrm{B}_u^- \rightarrow 1^1\mathrm{B}_u^+ \rightarrow$<br>$\rightarrow 1^1\mathrm{B}_u^- \rightarrow 1^1\mathrm{B}_u^+ \rightarrow 1^1\mathrm{B}_u^- \rightarrow 2^1\mathrm{A}_g^-{+}1^1\mathrm{A}_g^- \rightarrow$<br>$\rightarrow 1^1\mathrm{B}_u^- \rightarrow 2^1\mathrm{A}_g^-{+}1^1\mathrm{A}_g^- \rightarrow 1^1\mathrm{B}_u^- \rightarrow 2^1\mathrm{A}_g^-{+}1^1\mathrm{A}_g^- \rightarrow 1^1\mathrm{B}_u^- \rightarrow$<br>$\rightarrow 2^1\mathrm{A}_g^-{+}1^1\mathrm{A}_g^- \rightarrow 1^1\mathrm{B}_u^- \rightarrow 2^1\mathrm{A}_g^-{+}1^1\mathrm{A}_g^- \rightarrow 1^1\mathrm{B}_u^-$	1	0.5
$1^1\mathrm{B}_u^+ \rightarrow 1^1\mathrm{B}_u^- \rightarrow 1^1\mathrm{B}_u^+ \rightarrow 1^1\mathrm{B}_u^- \rightarrow 1^1\mathrm{B}_u^+ \rightarrow$<br>$\rightarrow 2^1\mathrm{A}_g^-{+}1^1\mathrm{A}_g^-$	1	0.5
$1^1\mathrm{B}_u^+ \rightarrow 1^1\mathrm{B}_u^- \rightarrow 2^1\mathrm{A}_g^-{+}1^1\mathrm{A}_g^- \rightarrow 1^1\mathrm{B}_u^- \rightarrow 2^1\mathrm{A}_g^-{+}1^1\mathrm{A}_g^- \rightarrow$<br>$\rightarrow 1^1\mathrm{B}_u^- \rightarrow 2^1\mathrm{A}_g^-{+}1^1\mathrm{A}_g^- \rightarrow 1^1\mathrm{B}_u^- \rightarrow 2^1\mathrm{A}_g^-{+}1^1\mathrm{A}_g^- \rightarrow$<br>$\rightarrow 1^1\mathrm{B}_u^- \rightarrow 2^1\mathrm{A}_g^-{+}1^1\mathrm{A}_g^- \rightarrow 1^1\mathrm{B}_u^-$	1	0.5
$1^1\mathrm{B}_u^+ \rightarrow 1^1\mathrm{B}_u^- \rightarrow 1^1\mathrm{B}_u^+ \rightarrow 1^1\mathrm{B}_u^- \rightarrow 2^1\mathrm{A}_g^-{+}1^1\mathrm{A}_g^- \rightarrow$<br>$\rightarrow 1^1\mathrm{B}_u^- \rightarrow 2^1\mathrm{A}_g^-{+}1^1\mathrm{A}_g^- \rightarrow 1^1\mathrm{B}_u^- \rightarrow 2^1\mathrm{A}_g^-{+}1^1\mathrm{A}_g^- \rightarrow$<br>$\rightarrow 1^1\mathrm{B}_u^- \rightarrow 2^1\mathrm{A}_g^-{+}1^1\mathrm{A}_g^- \rightarrow 1^1\mathrm{B}_u^- \rightarrow 2^1\mathrm{A}_g^-{+}1^1\mathrm{A}_g^- \rightarrow 1^1\mathrm{B}_u^- \rightarrow$<br>$\rightarrow 2^1\mathrm{A}_g^-{+}1^1\mathrm{A}_g^-$	1	0.5
$1^1\mathrm{B}_u^+ \rightarrow 1^1\mathrm{B}_u^- \rightarrow 1^1\mathrm{B}_u^+ \rightarrow 1^1\mathrm{B}_u^- \rightarrow 1^1\mathrm{B}_u^+ \rightarrow$<br>$\rightarrow 1^1\mathrm{B}_u^- \rightarrow 1^1\mathrm{B}_u^+ \rightarrow 1^1\mathrm{B}_u^- \rightarrow 1^1\mathrm{B}_u^+ \rightarrow$<br>$\rightarrow 1^1\mathrm{B}_u^- \rightarrow 1^1\mathrm{B}_u^+ \rightarrow 1^1\mathrm{B}_u^- \rightarrow 1^1\mathrm{B}_u^+ \rightarrow 1^1\mathrm{B}_u^-$	1	0.5
$1^1\mathrm{B}_u^+ \rightarrow 1^1\mathrm{B}_u^- \rightarrow 1^1\mathrm{B}_u^+ \rightarrow 1^1\mathrm{B}_u^- \rightarrow 2^1\mathrm{A}_g^-{+}1^1\mathrm{A}_g^- \rightarrow$<br>$\rightarrow 1^1\mathrm{B}_u^- \rightarrow 2^1\mathrm{A}_g^-{+}1^1\mathrm{A}_g^- \rightarrow 1^1\mathrm{B}_u^- \rightarrow 2^1\mathrm{A}_g^-{+}1^1\mathrm{A}_g^- \rightarrow$<br>$\rightarrow 1^1\mathrm{B}_u^- \rightarrow 2^1\mathrm{A}_g^-{+}1^1\mathrm{A}_g^- \rightarrow 1^1\mathrm{B}_u^- \rightarrow 2^1\mathrm{A}_g^-{+}1^1\mathrm{A}_g^- \rightarrow 1^1\mathrm{B}_u^- \rightarrow$<br>$\rightarrow 2^1\mathrm{A}_g^-{+}1^1\mathrm{A}_g^- \rightarrow 1^1\mathrm{B}_u^- \rightarrow 2^1\mathrm{A}_g^-{+}1^1\mathrm{A}_g^- \rightarrow 1^1\mathrm{B}_u^- \rightarrow$<br>$\rightarrow 2^1\mathrm{A}_g^-{+}1^1\mathrm{A}_g^- \rightarrow 1^1\mathrm{B}_u^- \rightarrow 2^1\mathrm{A}_g^-{+}1^1\mathrm{A}_g^-$	1	0.5
$1^1\mathrm{B}_u^+ \rightarrow 1^1\mathrm{B}_u^- \rightarrow 1^1\mathrm{B}_u^+ \rightarrow 1^1\mathrm{B}_u^- \rightarrow 1^1\mathrm{B}_u^+ \rightarrow$<br>$\rightarrow 1^1\mathrm{B}_u^- \rightarrow 1^1\mathrm{B}_u^+ \rightarrow 1^1\mathrm{B}_u^- \rightarrow 1^1\mathrm{B}_u^+ \rightarrow$<br>$\rightarrow 1^1\mathrm{B}_u^- \rightarrow 1^1\mathrm{B}_u^+ \rightarrow 1^1\mathrm{B}_u^- \rightarrow 2^1\mathrm{A}_g^-{+}1^1\mathrm{A}_g^- \rightarrow 1^1\mathrm{B}_u^- \rightarrow$		

$\rightarrow 2^1A_g^-+1^1A_g^- \rightarrow 1^1B_u^- \rightarrow 2^1A_g^-+1^1A_g^- \rightarrow 1^1B_u^- \rightarrow$		
$\rightarrow 2^1A_g^-+1^1A_g^- \rightarrow 1^1B_u^-$	1	0.5
$1^1B_u^+ \rightarrow 1^1B_u^- \rightarrow 1^1B_u^+ \rightarrow 1^1B_u^- \rightarrow 1^1B_u^+ \rightarrow$		
$\rightarrow 1^1B_u^- \rightarrow 1^1B_u^+ \rightarrow 1^1B_u^- \rightarrow 1^1B_u^+ \rightarrow$		
$\rightarrow 1^1B_u^- \rightarrow 1^1B_u^+ \rightarrow 1^1B_u^-$	1	0.5
$1^1B_u^+ \rightarrow 1^1B_u^- \rightarrow 1^1B_u^+ \rightarrow 1^1B_u^- \rightarrow 1^1B_u^+ \rightarrow$		
$\rightarrow 1^1B_u^- \rightarrow 2^1A_g^-+1^1A_g^- \rightarrow 1^1B_u^- \rightarrow 2^1A_g^-+1^1A_g^- \rightarrow$		
$\rightarrow 1^1B_u^- \rightarrow 2^1A_g^-+1^1A_g^- \rightarrow 1^1B_u^- \rightarrow 2^1A_g^-+1^1A_g^- \rightarrow 1^1B_u^- \rightarrow$		
$\rightarrow 2^1A_g^-+1^1A_g^- \rightarrow 1^1B_u^- \rightarrow 2^1A_g^-+1^1A_g^- \rightarrow 1^1B_u^- \rightarrow$		
$\rightarrow 2^1A_g^-+1^1A_g^- \rightarrow 1^1B_u^- \rightarrow 2^1A_g^-+1^1A_g^- \rightarrow 1^1B_u^-$	1	0.5
$1^1B_u^+ \rightarrow 1^1B_u^- \rightarrow 2^1A_g^-+1^1A_g^- \rightarrow 1^1B_u^- \rightarrow 2^1A_g^-+1^1A_g^- \rightarrow$		
$\rightarrow 1^1B_u^- \rightarrow 2^1A_g^-+1^1A_g^- \rightarrow 1^1B_u^- \rightarrow 2^1A_g^-+1^1A_g^- \rightarrow$		
$\rightarrow 1^1B_u^- \rightarrow 2^1A_g^-+1^1A_g^- \rightarrow 1^1B_u^- \rightarrow 2^1A_g^-+1^1A_g^- \rightarrow 1^1B_u^- \rightarrow$		
$\rightarrow 2^1A_g^-+1^1A_g^- \rightarrow 1^1B_u^- \rightarrow 2^1A_g^-+1^1A_g^- \rightarrow 1^1B_u^- \rightarrow$		
$\rightarrow 2^1A_g^-+1^1A_g^- \rightarrow 1^1B_u^- \rightarrow 2^1A_g^-+1^1A_g^- \rightarrow 1^1B_u^- \rightarrow 2^1A_g^-+1^1A_g^- \rightarrow$		
$\rightarrow 1^1B_u^- \rightarrow 2^1A_g^-+1^1A_g^- \rightarrow 1^1B_u^-$	1	0.5

Table S8: (continued from previous page)

Pathway	# Traj.	%
$1^1\mathrm{B}_u^+ \rightarrow 1^1\mathrm{B}_u^- \rightarrow 1^1\mathrm{B}_u^+ \rightarrow 1^1\mathrm{B}_u^- \rightarrow 1^1\mathrm{B}_u^+ \rightarrow$		
$\rightarrow 1^1\mathrm{B}_u^- \rightarrow 1^1\mathrm{B}_u^+ \rightarrow 1^1\mathrm{B}_u^- \rightarrow 2^1\mathrm{A}_g^-{+}1^1\mathrm{A}_g^- \rightarrow$		
$\rightarrow 1^1\mathrm{B}_u^- \rightarrow 2^1\mathrm{A}_g^-{+}1^1\mathrm{A}_g^-$	1	0.5
$1^1\mathrm{B}_u^+ \rightarrow 1^1\mathrm{B}_u^- \rightarrow 1^1\mathrm{B}_u^+ \rightarrow 1^1\mathrm{B}_u^- \rightarrow 1^1\mathrm{B}_u^+ \rightarrow$		
$\rightarrow 1^1\mathrm{B}_u^- \rightarrow 1^1\mathrm{B}_u^+ \rightarrow 1^1\mathrm{B}_u^- \rightarrow 2^1\mathrm{A}_g^-{+}1^1\mathrm{A}_g^- \rightarrow$		
$\rightarrow 1^1\mathrm{B}_u^- \rightarrow 2^1\mathrm{A}_g^-{+}1^1\mathrm{A}_g^- \rightarrow 1^1\mathrm{B}_u^- \rightarrow 2^1\mathrm{A}_g^-{+}1^1\mathrm{A}_g^- \rightarrow 1^1\mathrm{B}_u^-$	1	0.5
$1^1\mathrm{B}_u^+ \rightarrow 1^1\mathrm{B}_u^- \rightarrow 1^1\mathrm{B}_u^+ \rightarrow 1^1\mathrm{B}_u^- \rightarrow 1^1\mathrm{B}_u^+ \rightarrow$		
$\rightarrow 1^1\mathrm{B}_u^- \rightarrow 1^1\mathrm{B}_u^+ \rightarrow 1^1\mathrm{B}_u^- \rightarrow 1^1\mathrm{B}_u^+ \rightarrow$		
$\rightarrow 1^1\mathrm{B}_u^- \rightarrow 2^1\mathrm{A}_g^-{+}1^1\mathrm{A}_g^- \rightarrow 1^1\mathrm{B}_u^- \rightarrow 2^1\mathrm{A}_g^-{+}1^1\mathrm{A}_g^- \rightarrow 1^1\mathrm{B}_u^- \rightarrow$		
$\rightarrow 2^1\mathrm{A}_g^-{+}1^1\mathrm{A}_g^- \rightarrow 1^1\mathrm{B}_u^- \rightarrow 2^1\mathrm{A}_g^-{+}1^1\mathrm{A}_g^- \rightarrow 1^1\mathrm{B}_u^-$	1	0.5
$1^1\mathrm{B}_u^+ \rightarrow 1^1\mathrm{B}_u^- \rightarrow 1^1\mathrm{B}_u^+ \rightarrow 1^1\mathrm{B}_u^- \rightarrow 1^1\mathrm{B}_u^+ \rightarrow$		
$\rightarrow 1^1\mathrm{B}_u^- \rightarrow 2^1\mathrm{A}_g^-{+}1^1\mathrm{A}_g^- \rightarrow 1^1\mathrm{B}_u^- \rightarrow 2^1\mathrm{A}_g^-{+}1^1\mathrm{A}_g^- \rightarrow$		
$\rightarrow 1^1\mathrm{B}_u^- \rightarrow 2^1\mathrm{A}_g^-{+}1^1\mathrm{A}_g^- \rightarrow 1^1\mathrm{B}_u^- \rightarrow 2^1\mathrm{A}_g^-{+}1^1\mathrm{A}_g^- \rightarrow 1^1\mathrm{B}_u^- \rightarrow$		
$\rightarrow 2^1\mathrm{A}_g^-{+}1^1\mathrm{A}_g^-$	1	0.5
$1^1\mathrm{B}_u^- \rightarrow 1^1\mathrm{B}_u^+ \rightarrow 1^1\mathrm{B}_u^- \rightarrow 1^1\mathrm{B}_u^+ \rightarrow 1^1\mathrm{B}_u^- \rightarrow$		
$\rightarrow 2^1\mathrm{A}_g^-{+}1^1\mathrm{A}_g^-$	1	0.5

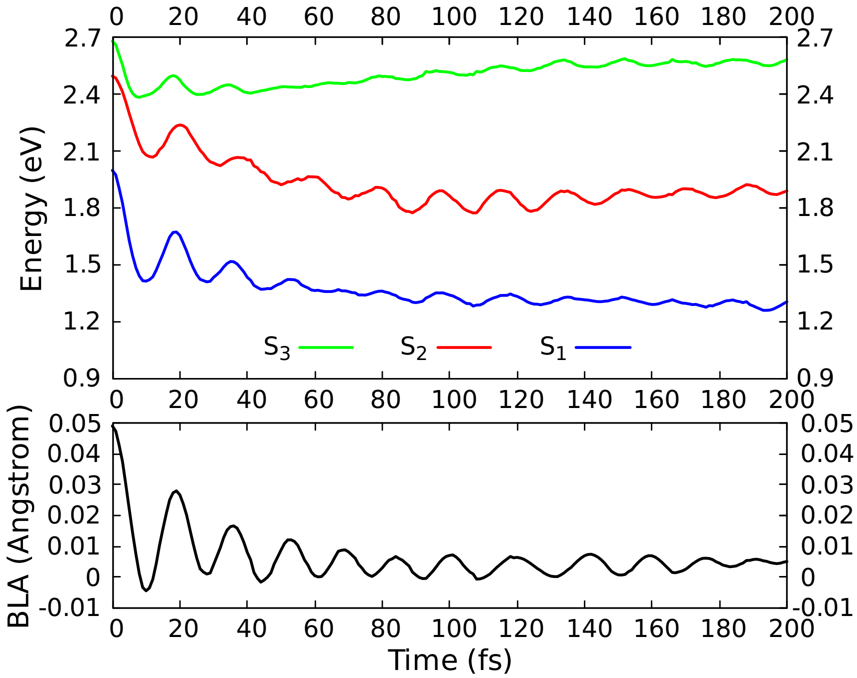

Figure S9: Energies (eV) of the three low-lying singlet adiabatic excited states relative to the ground state (S_0), upper panel, and bond length alternation (BLA, Å), lower panel, as functions of time obtained from the excited state simulations of lutein (QM part) in methanol solution (MM part). The reported results are obtained by averaging over all trajectories and time intervals of 1 fs.

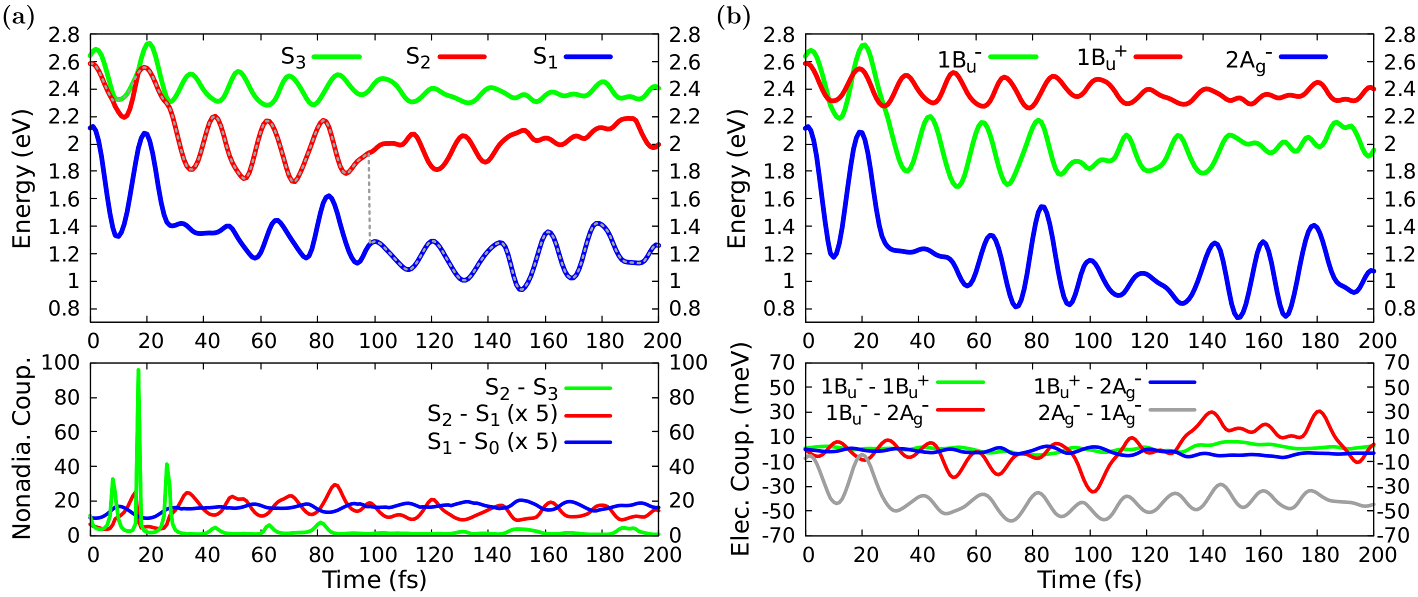

Figure S10: Energies (eV) of the three low-lying excited states in both the adiabatic basis (panel a) and the diabatic one (panel b) relative to the ground state (S_0), and couplings between states computed along a representative SH trajectory for lutein in methanol. For the nonadiabatic couplings (panel a), the vector modulus (in a.u.) is reported.

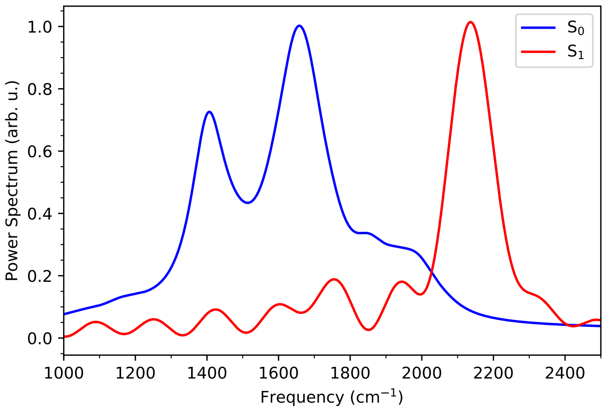

Figure S11: Power spectrum of the BLA coordinate computed over the last 10 ps of the S_0 equilibration dynamics in methanol (blue line) and over the S_1 SH trajectories (red line). Only the SH trajectories that remain in S_1 for at least 175 fs were included in this analysis, to extract the S_1 power spectrum. All power spectra were computed from the autocorrelation function of the BLA, multiplied by a damping factor e^{-t^2/σ^2} with $\sigma = 100$ fs.

S3.3 Fitting of the state populations

In order to extract the state lifetimes, the diabatic populations obtained in the excited state dynamics simulations for lutein were fitted using the following rate model:

$$1^1\mathrm{B}_u^+ \xrightarrow{1/\tau_{2\mathrm{x}}} 1^1\mathrm{B}_u^- \xrightarrow{1/\tau_{\mathrm{x}1}} 2^1\mathrm{A}_g^-, 1^1\mathrm{A}_g^- \tag{S6}$$

where $\tau_{2\mathrm{x}}$ and $\tau_{\mathrm{x}1}$ are time constants. In particular, the decay of the initially populated $1^1\mathrm{B}_u^+$ state was modelled by an exponential function:

$$X(t) = X(0)\, e^{-t/\tau_{2\mathrm{x}}}. \tag{S7}$$

Assuming a monoexponential decay of the intermediate state $1^1\mathrm{B}_u^-$, its population was then fitted using the following function:

$$Y(t) = Y(0)\, e^{-t/\tau_{\mathrm{x}1}} + X(0)\, \frac{\tau_{\mathrm{x}1}}{\tau_{\mathrm{x}1} - \tau_{2\mathrm{x}}} \big(e^{-t/\tau_{\mathrm{x}1}} - e^{-t/\tau_{2\mathrm{x}}}\big). \tag{S8}$$

Since the sum of the populations is equal to 1 at each time, the population rise of final states $2^1\mathrm{A}_g^-$ and $1^1\mathrm{A}_g^-$ is given by:

$$Z(t) = 1 - X(t) - Y(t). \tag{S9}$$

In the fitting procedure, $X(0)$ and $Y(0)$ are set equal to the initial populations of the $1^1\mathrm{B}_u^+$ and $1^1\mathrm{B}_u^-$ states, respectively; then, $\tau_{2\mathrm{x}}$ was determined by fitting $X(t)$, while $\tau_{\mathrm{x}1}$ was extracted by subsequently fitting $Y(t)$.

S3.4 State lifetimes

Table S9: Lifetimes (fs) of the S_2 ($1B_u^+$) and S_x ($1B_u^-$) states for lutein and other carotenoids determined in the present work and in experimental investigations in solution. For each carotenoid, the number of C=C bonds (N) in the conjugated polyene chain is reported in parenthesis.

Carotenoid	State lifetime (fs) S_2 ($1^1B_u^+$)	S_x ($1^1B_u^-$)	Solvent	Technique	Reference
lutein (N∼10)	22	132	methanol	surface hopping	this work
	43 ± 5 - - 35 ± 2	≤ 100	hexane	pump-DFWM	[8]
	38 ± 3 - - 32 ± 3	≤ 100	THF	pump-DFWM	[8]
	35 ± 5 - - 23 ± 5	≤ 100	benzene	pump-DFWM	[8]
	≪ 100	< 600	hexane, diethyl ether, benzonitrile	transient absorption	[9]
neurosporene (N=9)	20 ± 5	400	cyclohexane	pump-probe	[10]
	79	265	hexane	transient abs.	[11]
	55	252	pentadecane	transient abs.	[11]
	40	192	benzene	transient abs.	[11]
	42	192	carbon disulfide	transient abs.	[11]
spheroidene (N=10)	27	275	cyclohexane	pump-probe	[12]
β-carotene (N∼11)	10 ± 2	150	cyclohexane	pump-probe	[13]
	≪ 100	< 600	hexane, diethyl ether, benzonitrile	transient absorption	[9]
lycopene (N=11)	9 ± 2	90	cyclohexane	pump-probe	[13]
	20	110	THF	pump-DFWM	[14]

S4 Reference states and orbitals

Table S10: CI coefficients, in the determinant basis, for the 4 low-lying singlet states of lutein at its S_0 minimum geometry (Table S12), computed with the R-AM1/FOMO-CASCI(6,6) method. These states were used as references in the construction of the diabatic states during the SH simulations. Each determinant is defined using the corresponding list of occupancies for the 6 active orbitals, which are: HOMO-2, HOMO-1, HOMO, LUMO, LUMO+1, LUMO+2. Notation for the orbital occupancy: 2, doubly occupied; +, singly occupied, spin alpha; -, singly occupied, spin beta; 0, empty.

#	Determinant	State			
		$1^1A_g^-$	$2^1A_g^-$	$1^1B_u^+$	$1^1B_u^-$
1	222000	-0.919189	-0.129629	-0.002755	-0.000119
2	22+-00	0.000594	0.003184	-0.642817	-0.011273
3	22+0-0	-0.029741	-0.292003	-0.000150	0.004213
4	22+00-	-0.000141	0.002712	-0.000510	0.327481
5	2+2-00	-0.040757	-0.283589	-0.001539	0.000336
6	2+20-0	-0.000609	-0.002012	0.128191	0.000732
7	2+200-	-0.011222	-0.074107	0.000070	0.001051
8	2++--0	0.048421	-0.146775	-0.000273	0.001177
9	2++-0-	-0.000313	0.001470	0.030623	0.087299
10	2++0--	-0.021654	-0.000159	-0.000138	0.000629
11	+22-00	0.000097	0.001118	-0.013063	0.294108
12	+220-0	0.018619	0.068585	0.000387	-0.000109
13	+2200-	-0.000230	-0.000381	0.045130	0.002206
14	+2+--0	0.000097	0.000034	-0.028556	0.083430
15	+2+-0-	0.036816	-0.058843	-0.000219	-0.000139
16	+2+0--	0.000127	-0.000557	-0.008536	-0.063114
17	++2--0	0.021213	0.003408	0.000021	0.000449
18	++2-0-	0.000087	-0.000005	-0.010637	0.063577

19	++20--	-0.018897	0.018563	0.000159	-0.000312
20	+++---	0.000038	-0.000032	-0.021440	0.000070
21	22-+00	-0.000594	-0.003184	0.642817	0.011273
22	220200	0.206721	-0.599797	-0.002399	0.001844
23	220+-0	0.001546	-0.003288	-0.119699	-0.340255
24	220+0-	0.027426	0.082572	0.000720	0.001041
25	2+-200	-0.000929	0.000122	0.133009	-0.322983
26	2+-+-0	0.058008	-0.004295	0.000524	-0.000144
27	2+-+0-	-0.000908	-0.000559	0.032663	-0.027126
28	2+02-0	0.000004	0.000157	-0.029477	0.002536
29	2+020-	-0.004830	0.055103	0.000447	-0.000689
30	2+0+--	0.000065	0.000273	-0.011045	-0.024106
31	+2-200	0.026281	0.078412	0.000117	-0.001501
32	+2-+-0	0.000305	0.000049	-0.034712	-0.023901
33	+2-+0-	0.044415	-0.021112	-0.000203	0.000573
34	+202-0	0.002411	-0.047758	-0.000438	0.000517
35	+2020-	0.000010	0.000497	-0.002727	-0.000729
36	+20+--	0.000236	-0.014846	-0.000049	0.000162

Table S10: (continued from previous page)

#	Determinant	State			
		$1^1A_g^-$	$2^1A_g^-$	$1^1B_u^+$	$1^1B_u^-$
37	++-2-0	0.000009	0.000507	-0.013050	0.026211
38	++-20-	0.000443	0.014816	0.000091	-0.000299
39	++-+--	-0.000014	0.000075	0.005438	-0.000090
40	++02--	0.006523	-0.026943	-0.000180	0.000239
41	22-0+0	0.029741	0.292003	0.000150	-0.004213
42	220-+0	-0.001546	0.003288	0.119699	0.340255
43	220020	0.060438	0.134190	0.000412	-0.003664
44	2200+-	-0.000370	-0.001214	0.029311	-0.106821
45	2+--+0	-0.106429	0.151070	-0.000251	-0.001033
46	2+-020	0.000771	-0.001164	-0.046271	-0.045979
47	2+-0+-	-0.016016	-0.042580	0.000294	0.000735
48	2+0-20	0.002549	-0.040502	0.000341	0.000630
49	2+0-+-	-0.000067	0.000209	-0.012463	0.007116
50	2+002-	0.002875	-0.017810	-0.000238	-0.000117
51	+2--+0	-0.000402	-0.000083	0.063268	-0.059529
52	+2-020	-0.030620	-0.068641	-0.000225	0.000936
53	+2-0+-	0.000597	0.000394	-0.021361	0.037611
54	+20-20	0.000012	0.000213	-0.011166	-0.025122
55	+20-+-	-0.000416	0.002982	0.000367	0.000240
56	+2002-	-0.000008	-0.000232	-0.004373	0.012754
57	++--20	0.007811	0.016617	0.000104	-0.000215
58	++--+-	-0.000057	-0.000158	0.007394	0.004143
59	++-02-	0.000296	0.001040	-0.000100	-0.000076

60	++0-2-	-0.000071	0.000108	0.003590	-0.008647
61	22-00+	0.000141	-0.002712	0.000510	-0.327481
62	220-0+	-0.027426	-0.082572	-0.000720	-0.001041
63	2200-+	0.000370	0.001214	-0.029311	0.106821
64	220002	0.037379	0.078037	-0.000043	0.001902
65	2+--0+	0.001221	-0.000911	-0.063286	-0.060173
66	2+-0-+	0.037670	0.042739	-0.000156	-0.001363
67	2+-002	0.000343	0.000647	-0.012570	0.074456
68	2+0--+	0.000003	-0.000482	0.023508	0.016990
69	2+0-02	0.002898	0.031726	0.000293	-0.000441
70	2+00-2	0.000094	0.000046	-0.005560	-0.024297
71	+2--0+	-0.081231	0.079954	0.000422	-0.000433
72	+2-0-+	-0.000724	0.000163	0.029897	0.025504
73	+2-002	0.003460	0.002550	0.000246	0.000079
74	+20--+	0.000181	0.011865	-0.000317	-0.000401
75	+20-02	-0.000073	0.000263	0.000590	0.015351
76	+200-2	-0.002579	0.006205	0.000081	0.000211
77	++---+	0.000033	0.000115	0.008607	-0.004123

Table S10: (continued from previous page)

#	Determinant	State			
		$1^1A_g^-$	$2^1A_g^-$	$1^1B_u^+$	$1^1B_u^-$
78	++--02	-0.001446	0.013921	-0.000011	-0.000262
79	++-0-2	0.000001	0.000022	-0.001683	-0.000007
80	++0--2	-0.003734	0.001533	0.000014	-0.000149
81	2-2+00	0.040757	0.283589	0.001539	-0.000336
82	2-+200	0.000929	-0.000122	-0.133009	0.322983
83	2-++-0	-0.106429	0.151070	-0.000251	-0.001033
84	2-++0-	0.001221	-0.000911	-0.063286	-0.060173
85	202200	0.056646	0.119552	0.000432	0.002007
86	202+-0	-0.000559	0.000929	0.047783	-0.038297
87	202+0-	-0.032049	-0.067552	-0.000771	0.000564
88	20+2-0	-0.000141	-0.034974	-0.000139	0.000063
89	20+20-	0.000004	-0.000130	0.012067	-0.027945
90	20++--	0.006903	0.017946	-0.000017	-0.000155
91	+-2200	0.000090	0.000878	0.023491	0.096426
92	+-2+-0	0.014459	0.037458	0.000042	0.000458
93	+-2+0-	0.000239	-0.000343	-0.018250	-0.037873
94	+-+2-0	0.000008	-0.000007	-0.010845	-0.008050
95	+-+20-	0.000788	-0.004674	-0.000080	0.000256
96	+-++--	-0.000018	-0.000057	0.006996	-0.006253
97	+022-0	-0.002974	0.015236	0.000095	-0.000206
98	+0220-	0.000024	-0.000281	-0.003631	-0.012148
99	+02+--	-0.000927	-0.001429	0.000019	-0.000036
100	+0+2--	-0.000018	0.000156	0.003357	0.008973

101	2-20+0	0.000609	0.002012	-0.128191	-0.000732
102	2-+-+0	0.058008	-0.004295	0.000524	-0.000144
103	2-+020	-0.000771	0.001164	0.046271	0.045979
104	2-+0+-	0.037670	0.042739	-0.000156	-0.001363
105	202-+0	0.000559	-0.000929	-0.047783	0.038297
106	202020	0.088711	-0.090864	0.000101	0.001039
107	2020+-	-0.000839	0.000670	0.020806	0.017843
108	20+-20	-0.000066	0.000274	0.059509	0.001731
109	20+-+-	-0.002067	-0.009002	-0.000474	0.000054
110	20+02-	0.000097	0.000146	-0.008541	-0.025646
111	+-2-+0	-0.035672	-0.040866	-0.000063	-0.000907
112	+-2020	-0.000279	0.000235	0.020814	-0.015059
113	+-20+-	-0.035868	0.007098	-0.000185	0.000094
114	+-+-20	-0.001951	-0.008178	-0.000025	0.000210
115	+-+-+-	0.000047	0.000131	-0.017235	0.000919
116	+-+02-	0.001891	0.018510	0.000109	-0.000127
117	+02-20	-0.000053	-0.000274	0.009314	-0.023782
118	+02-+-	-0.002141	-0.017681	-0.000111	0.000282

Table S10: (continued from previous page)

#	Determinant	State			
		$1^1A_g^-$	$2^1A_g^-$	$1^1B_u^+$	$1^1B_u^-$
119	+0202-	0.000020	0.000100	-0.006019	0.000194
120	+0+-2-	-0.008166	0.012931	0.000076	-0.000119
121	2-200+	0.011222	0.074107	-0.000070	-0.001051
122	2-+-0+	-0.000908	-0.000559	0.032663	-0.027126
123	2-+0-+	-0.016016	-0.042580	0.000294	0.000735
124	2-+002	-0.000343	-0.000647	0.012570	-0.074456
125	202-0+	0.032049	0.067552	0.000771	-0.000564
126	2020-+	0.000839	-0.000670	-0.020806	-0.017843
127	202002	0.034997	0.010281	-0.000283	-0.001143
128	20+--+	-0.004836	-0.008944	0.000491	0.000101
129	20+-02	-0.000046	-0.000198	0.029797	-0.010323
130	20+0-2	0.002635	0.022048	-0.000016	-0.000441
131	+-2-0+	-0.000325	0.000348	0.028887	-0.025704
132	+-20-+	0.054766	-0.025661	0.000027	0.000218
133	+-2002	0.000810	-0.000419	-0.016975	-0.031845
134	+-+--+	-0.000067	-0.000043	0.031679	0.005265
135	+-+-02	0.000000	-0.006859	0.000473	0.000313
136	+-+0-2	0.000016	0.000068	0.002167	-0.016434
137	+02--+	0.003067	0.019110	0.000092	-0.000246
138	+02-02	0.000050	0.000103	-0.001441	-0.016067
139	+020-2	0.000572	0.001225	-0.000166	-0.000112
140	+0+--2	-0.000071	0.000076	0.002171	-0.010674
141	2--++0	0.048421	-0.146775	-0.000273	0.001177

142	2-02+0	-0.000004	-0.000157	0.029477	-0.002536
143	2-0+20	-0.002549	0.040502	-0.000341	-0.000630
144	2-0++-	0.000003	-0.000482	0.023508	0.016990
145	20-2+0	0.000141	0.034974	0.000139	-0.000063
146	20-+20	0.000066	-0.000274	-0.059509	-0.001731
147	20-++-	-0.004836	-0.008944	0.000491	0.000101
148	200220	-0.039434	0.086767	0.000409	-0.000679
149	2002+-	0.000370	-0.000609	-0.015610	0.030773
150	200+2-	0.002937	0.007053	0.000157	0.000004
151	+--2+0	-0.000018	-0.000500	0.023895	-0.018161
152	+--+20	-0.005860	-0.008439	-0.000079	0.000004
153	+--++-	0.000004	0.000041	0.018846	0.001212
154	+-0220	0.000144	-0.000482	-0.013638	-0.030370
155	+-02+-	0.013504	-0.018015	-0.000032	0.000196
156	+-0+2-	-0.000098	0.000056	0.007647	0.019167
157	+0-220	0.002423	0.006848	0.000060	0.000031
158	+0-2+-	-0.000102	-0.000081	0.008474	-0.019013
159	+0-+2-	-0.009021	0.004288	0.000063	-0.000067

Table S10: (continued from previous page)

#	Determinant	State			
		$1^1A_g^-$	$2^1A_g^-$	$1^1B_u^+$	$1^1B_u^-$
160	+0022-	0.000002	0.000048	0.001515	-0.000142
161	2--+0+	-0.000313	0.001470	0.030623	0.087299
162	2-020+	0.004830	-0.055103	-0.000447	0.000689
163	2-0+-+	-0.000067	0.000209	-0.012463	0.007116
164	2-0+02	-0.002898	-0.031726	-0.000293	0.000441
165	20-20+	-0.000004	0.000130	-0.012067	0.027945
166	20-+-+	-0.002067	-0.009002	-0.000474	0.000054
167	20-+02	0.000046	0.000198	-0.029797	0.010323
168	2002-+	-0.000370	0.000609	0.015610	-0.030773
169	200202	-0.012234	0.000087	0.000292	-0.000428
170	200+-2	-0.000055	0.000087	0.005799	0.030495
171	+--20+	-0.001231	-0.010143	-0.000011	0.000043
172	+--+-+	0.000028	-0.000059	-0.031281	0.005132
173	+--+02	0.001446	-0.007062	-0.000461	-0.000051
174	+-02-+	-0.020027	0.044958	0.000212	-0.000435
175	+-0202	-0.000301	0.000559	0.006472	0.000884
176	+-0+-2	-0.002928	-0.008573	0.000131	0.000223
177	+0-2-+	0.000120	-0.000075	-0.011831	0.010040
178	+0-202	-0.004980	-0.009657	-0.000143	0.000294
179	+0-+-2	-0.000092	0.000034	0.003205	0.005323
180	+002-2	-0.000018	-0.004767	0.000023	0.000051
181	2--0++	-0.021654	-0.000159	-0.000138	0.000629
182	2-0-++	0.000065	0.000273	-0.011045	-0.024106

183	2-002+	-0.002875	0.017810	0.000238	0.000117
184	2-00+2	-0.000094	-0.000046	0.005560	0.024297
185	20--++	0.006903	0.017946	-0.000017	-0.000155
186	20-02+	-0.000097	-0.000146	0.008541	0.025646
187	20-0+2	-0.002635	-0.022048	0.000016	0.000441
188	200-2+	-0.002937	-0.007053	-0.000157	-0.000004
189	200-+2	0.000055	-0.000087	-0.005799	-0.030495
190	200022	-0.007316	-0.011443	-0.000067	0.000341
191	+---++	0.000006	-0.000014	-0.009005	-0.006274
192	+--02+	-0.002188	-0.019549	-0.000008	0.000203
193	+--0+2	-0.000016	-0.000090	-0.000484	0.016441
194	+-0-2+	0.000170	-0.000163	-0.011237	-0.010520
195	+-0-+2	0.006662	0.007041	-0.000145	-0.000074
196	+-0022	-0.000034	0.000017	-0.000446	0.014900
197	+0--2+	0.017188	-0.017218	-0.000139	0.000186
198	+0--+2	0.000164	-0.000110	-0.005376	0.005350
199	+0-022	0.003074	0.006575	0.000046	-0.000100
200	+00-22	0.000014	0.000001	0.000551	-0.000975

Table S10: (continued from previous page)

#	Determinant	State			
		$1^1A_g^-$	$2^1A_g^-$	$1^1B_u^+$	$1^1B_u^-$
201	-22+00	-0.000097	-0.001118	0.013063	-0.294108
202	-2+200	-0.026281	-0.078412	-0.000117	0.001501
203	-2++-0	-0.000402	-0.000083	0.063268	-0.059529
204	-2++0-	-0.081231	0.079954	0.000422	-0.000433
205	-+2200	-0.000090	-0.000878	-0.023491	-0.096426
206	-+2+-0	-0.035672	-0.040866	-0.000063	-0.000907
207	-+2+0-	-0.000325	0.000348	0.028887	-0.025704
208	-++2-0	-0.000018	-0.000500	0.023895	-0.018161
209	-++20-	-0.001231	-0.010143	-0.000011	0.000043
210	-+++--	-0.000006	0.000014	0.009005	0.006274
211	022200	0.034345	0.072537	0.000428	-0.000434
212	022+-0	-0.000022	0.000454	0.007280	0.067140
213	022+0-	0.003543	0.001767	-0.000024	-0.000605
214	02+2-0	0.002054	0.028756	0.000021	-0.000229
215	02+20-	0.000004	-0.000119	-0.000957	0.010811
216	02++--	-0.000689	0.011979	0.000112	-0.000079
217	0+22-0	0.000038	0.000189	-0.006105	0.018836
218	0+220-	0.002094	-0.006326	-0.000104	0.000135
219	0+2+--	0.000008	0.000032	-0.002030	-0.000007
220	0++2--	0.003509	-0.001232	0.000009	-0.000011
221	-220+0	-0.018619	-0.068585	-0.000387	0.000109
222	-2+-+0	0.000305	0.000049	-0.034712	-0.023901
223	-2+020	0.030620	0.068641	0.000225	-0.000936

224	-2+0+-	-0.000724	0.000163	0.029897	0.025504
225	-+2-+0	0.014459	0.037458	0.000042	0.000458
226	-+2020	0.000279	-0.000235	-0.020814	0.015059
227	-+20+-	0.054766	-0.025661	0.000027	0.000218
228	-++-20	-0.005860	-0.008439	-0.000079	0.000004
229	-++-+-	-0.000028	0.000059	0.031281	-0.005132
230	-++02-	-0.002188	-0.019549	-0.000008	0.000203
231	022-+0	0.000022	-0.000454	-0.007280	-0.067140
232	022020	0.032475	0.007312	-0.000051	0.000734
233	0220+-	0.000181	-0.000033	-0.017337	0.030063
234	02+-20	-0.000043	-0.000254	0.027521	0.008533
235	02+-+-	-0.000246	-0.005597	0.000013	-0.000201
236	02+02-	-0.000029	-0.000038	0.001929	-0.016827
237	0+2-20	0.002771	0.021229	0.000052	-0.000070
238	0+2-+-	-0.000028	0.000010	0.001584	0.015279
239	0+202-	-0.001469	-0.000203	0.000013	-0.000045
240	0++-2-	0.000011	-0.000114	-0.002094	-0.010346
241	-2200+	0.000230	0.000381	-0.045130	-0.002206

Table S10: (continued from previous page)

#	Determinant	State			
		$1^1A_g^-$	$2^1A_g^-$	$1^1B_u^+$	$1^1B_u^-$
242	-2+-0+	0.044415	-0.021112	-0.000203	0.000573
243	-2+0-+	0.000597	0.000394	-0.021361	0.037611
244	-2+002	-0.003460	-0.002550	-0.000246	-0.000079
245	-+2-0+	0.000239	-0.000343	-0.018250	-0.037873
246	-+20-+	-0.035868	0.007098	-0.000185	0.000094
247	-+2002	-0.000810	0.000419	0.016975	0.031845
248	-++--+	-0.000004	-0.000041	-0.018846	-0.001212
249	-++-02	0.001446	-0.007062	-0.000461	-0.000051
250	-++0-2	-0.000016	-0.000090	-0.000484	0.016441
251	022-0+	-0.003543	-0.001767	0.000024	0.000605
252	0220-+	-0.000181	0.000033	0.017337	-0.030063
253	022002	0.049650	-0.028993	-0.000214	0.000035
254	02+--+	0.000935	-0.006382	-0.000125	0.000280
255	02+-02	-0.000072	-0.000061	0.028320	0.000994
256	02+0-2	0.001071	0.010088	0.000055	-0.000131
257	0+2--+	0.000020	-0.000041	0.000446	-0.015272
258	0+2-02	0.001808	0.009030	0.000086	-0.000249
259	0+20-2	0.000013	0.000021	-0.006390	0.000065
260	0++--2	-0.004503	0.012244	0.000100	-0.000160
261	-2-++0	0.000097	0.000034	-0.028556	0.083430
262	-202+0	-0.002411	0.047758	0.000438	-0.000517
263	-20+20	-0.000012	-0.000213	0.011166	0.025122
264	-20++-	0.000181	0.011865	-0.000317	-0.000401

265	-+-2+0	0.000008	-0.000007	-0.010845	-0.008050
266	-+-+20	-0.001951	-0.008178	-0.000025	0.000210
267	-+-++-	0.000067	0.000043	-0.031679	-0.005265
268	-+0220	-0.000144	0.000482	0.013638	0.030370
269	-+02+-	-0.020027	0.044958	0.000212	-0.000435
270	-+0+2-	0.000170	-0.000163	-0.011237	-0.010520
271	02-2+0	-0.002054	-0.028756	-0.000021	0.000229
272	02-+20	0.000043	0.000254	-0.027521	-0.008533
273	02-++-	0.000935	-0.006382	-0.000125	0.000280
274	020220	-0.011683	0.000080	0.000041	0.000305
275	0202+-	-0.000031	0.000329	0.006165	-0.000323
276	020+2-	-0.004827	-0.009646	-0.000035	0.000018
277	0+-220	0.000036	0.000210	-0.006939	0.028322
278	0+-2+-	0.002877	0.007986	-0.000031	-0.000150
279	0+-+2-	0.000071	0.000041	-0.002770	0.005187
280	0+022-	0.000415	0.003975	0.000014	-0.000044
281	-2-+0+	0.036816	-0.058843	-0.000219	-0.000139
282	-2020+	-0.000010	-0.000497	0.002727	0.000729

Table S10: (continued from previous page)

#	Determinant	State			
		$1^1\mathrm{A}_g^-$	$2^1\mathrm{A}_g^-$	$1^1\mathrm{B}_u^+$	$1^1\mathrm{B}_u^-$
283	-20+-+	-0.000416	0.002982	0.000367	0.000240
284	-20+02	0.000073	-0.000263	-0.000590	-0.015351
285	-+-20+	0.000788	-0.004674	-0.000080	0.000256
286	-+-+-+	-0.000047	-0.000131	0.017235	-0.000919
287	-+-+02	0.000000	-0.006859	0.000473	0.000313
288	-+02-+	0.013504	-0.018015	-0.000032	0.000196
289	-+0202	0.000301	-0.000559	-0.006472	-0.000884
290	-+0+-2	0.006662	0.007041	-0.000145	-0.000074
291	02-20+	-0.000004	0.000119	0.000957	-0.010811
292	02-+-+	-0.000246	-0.005597	0.000013	-0.000201
293	02-+02	0.000072	0.000061	-0.028320	-0.000994
294	0202-+	0.000031	-0.000329	-0.006165	0.000323
295	020202	-0.020338	0.037900	0.000175	-0.000281
296	020+-2	-0.000165	0.000243	0.007111	0.012803
297	0+-2-+	-0.006386	-0.006754	0.000022	0.000161
298	0+-202	0.000049	-0.000219	-0.007423	0.011958
299	0+-+-2	-0.005458	0.003490	-0.000006	-0.000022
300	0+02-2	-0.000007	0.000049	0.002662	0.000355
301	-2-0++	0.000127	-0.000557	-0.008536	-0.063114
302	-20-++	0.000236	-0.014846	-0.000049	0.000162
303	-2002+	0.000008	0.000232	0.004373	-0.012754
304	-200+2	0.002579	-0.006205	-0.000081	-0.000211
305	-+--++	0.000018	0.000057	-0.006996	0.006253

306	-+-02+	0.001891	0.018510	0.000109	-0.000127
307	-+-0+2	0.000016	0.000068	0.002167	-0.016434
308	-+0-2+	-0.000098	0.000056	0.007647	0.019167
309	-+0-+2	-0.002928	-0.008573	0.000131	0.000223
310	-+0022	0.000034	-0.000017	0.000446	-0.014900
311	02--++	-0.000689	0.011979	0.000112	-0.000079
312	02-02+	0.000029	0.000038	-0.001929	0.016827
313	02-0+2	-0.001071	-0.010088	-0.000055	0.000131
314	020-2+	0.004827	0.009646	0.000035	-0.000018
315	020-+2	0.000165	-0.000243	-0.007111	-0.012803
316	020022	-0.004406	-0.007394	0.000082	0.000003
317	0+--2+	-0.000082	0.000074	0.004864	0.005158
318	0+--+2	0.009961	-0.015734	-0.000094	0.000182
319	0+-022	-0.000105	0.000101	0.005574	-0.004222
320	0+0-22	-0.000361	0.001353	-0.000046	-0.000006
321	--2++0	0.021213	0.003408	0.000021	0.000449
322	--+2+0	0.000009	0.000507	-0.013050	0.026211
323	--++20	0.007811	0.016617	0.000104	-0.000215

Table S10: (continued from previous page)

#	Determinant	State			
		$1^1A_g^-$	$2^1A_g^-$	$1^1B_u^+$	$1^1B_u^-$
324	--+++-	-0.000033	-0.000115	-0.008607	0.004123
325	-022+0	0.002974	-0.015236	-0.000095	0.000206
326	-02+20	0.000053	0.000274	-0.009314	0.023782
327	-02++-	0.003067	0.019110	0.000092	-0.000246
328	-0+220	-0.002423	-0.006848	-0.000060	-0.000031
329	-0+2+-	0.000120	-0.000075	-0.011831	0.010040
330	-0++2-	0.017188	-0.017218	-0.000139	0.000186
331	0-22+0	-0.000038	-0.000189	0.006105	-0.018836
332	0-2+20	-0.002771	-0.021229	-0.000052	0.000070
333	0-2++-	0.000020	-0.000041	0.000446	-0.015272
334	0-+220	-0.000036	-0.000210	0.006939	-0.028322
335	0-+2+-	-0.006386	-0.006754	0.000022	0.000161
336	0-++2-	-0.000082	0.000074	0.004864	0.005158
337	002220	-0.006463	-0.009947	-0.000040	-0.000319
338	0022+-	-0.000009	-0.000096	0.000115	-0.013858
339	002+2-	0.002822	0.006108	0.000044	0.000025
340	00+22-	0.000002	-0.000018	-0.000446	-0.000182
341	--2+0+	0.000087	-0.000005	-0.010637	0.063577
342	--+20+	0.000443	0.014816	0.000091	-0.000299
343	--++-+	0.000057	0.000158	-0.007394	-0.004143
344	--++02	-0.001446	0.013921	-0.000011	-0.000262
345	-0220+	-0.000024	0.000281	0.003631	0.012148
346	-02+-+	-0.002141	-0.017681	-0.000111	0.000282

347	-02+02	-0.000050	-0.000103	0.001441	0.016067
348	-0+2-+	-0.000102	-0.000081	0.008474	-0.019013
349	-0+202	0.004980	0.009657	0.000143	-0.000294
350	-0++-2	0.000164	-0.000110	-0.005376	0.005350
351	0-220+	-0.002094	0.006326	0.000104	-0.000135
352	0-2+-+	-0.000028	0.000010	0.001584	0.015279
353	0-2+02	-0.001808	-0.009030	-0.000086	0.000249
354	0-+2-+	0.002877	0.007986	-0.000031	-0.000150
355	0-+202	-0.000049	0.000219	0.007423	-0.011958
356	0-++-2	0.009961	-0.015734	-0.000094	0.000182
357	0022-+	0.000009	0.000096	-0.000115	0.013858
358	002202	-0.004254	-0.006674	0.000041	0.000134
359	002+-2	0.000097	-0.000107	-0.005154	-0.004329
360	00+2-2	-0.000061	0.000964	-0.000020	-0.000018
361	--20++	-0.018897	0.018563	0.000159	-0.000312
362	--+-++	0.000014	-0.000075	-0.005438	0.000090
363	--+02+	0.000296	0.001040	-0.000100	-0.000076
364	--+0+2	0.000001	0.000022	-0.001683	-0.000007

Table S10: (continued from previous page)

#	Determinant	State			
		$1^1A_g^-$	$2^1A_g^-$	$1^1B_u^+$	$1^1B_u^-$
365	-02-++	-0.000927	-0.001429	0.000019	-0.000036
366	-0202+	-0.000020	-0.000100	0.006019	-0.000194
367	-020+2	-0.000572	-0.001225	0.000166	0.000112
368	-0+-2+	-0.009021	0.004288	0.000063	-0.000067
369	-0+-+2	-0.000092	0.000034	0.003205	0.005323
370	-0+022	-0.003074	-0.006575	-0.000046	0.000100
371	0-2-++	0.000008	0.000032	-0.002030	-0.000007
372	0-202+	0.001469	0.000203	-0.000013	0.000045
373	0-20+2	-0.000013	-0.000021	0.006390	-0.000065
374	0-+-2+	0.000071	0.000041	-0.002770	0.005187
375	0-+-+2	-0.005458	0.003490	-0.000006	-0.000022
376	0-+022	0.000105	-0.000101	-0.005574	0.004222
377	002-2+	-0.002822	-0.006108	-0.000044	-0.000025
378	002-+2	-0.000097	0.000107	0.005154	0.004329
379	002022	-0.011084	0.010432	0.000099	-0.000110
380	00+-22	0.000022	-0.000024	-0.006633	-0.000020
381	---+++	-0.000038	0.000032	0.021440	-0.000070
382	--02++	0.006523	-0.026943	-0.000180	0.000239
383	--0+2+	-0.000071	0.000108	0.003590	-0.008647
384	--0++2	-0.003734	0.001533	0.000014	-0.000149
385	-0-2++	-0.000018	0.000156	0.003357	0.008973
386	-0-+2+	-0.008166	0.012931	0.000076	-0.000119
387	-0-++2	-0.000071	0.000076	0.002171	-0.010674

388	-0022+	-0.000002	-0.000048	-0.001515	0.000142
389	-002+2	0.000018	0.004767	-0.000023	-0.000051
390	-00+22	-0.000014	-0.000001	-0.000551	0.000975
391	0--2++	0.003509	-0.001232	0.000009	-0.000011
392	0--+2+	0.000011	-0.000114	-0.002094	-0.010346
393	0--++2	-0.004503	0.012244	0.000100	-0.000160
394	0-022+	-0.000415	-0.003975	-0.000014	0.000044
395	0-02+2	0.000007	-0.000049	-0.002662	-0.000355
396	0-0+22	0.000361	-0.001353	0.000046	0.000006
397	00-22+	-0.000002	0.000018	0.000446	0.000182
398	00-2+2	0.000061	-0.000964	0.000020	0.000018
399	00-+22	-0.000022	0.000024	0.006633	0.000020
400	000222	0.006761	-0.007716	-0.000115	0.000086

Table S11: Active molecular orbitals (MOs) coefficients, in the atomic orbital (AO) basis, of lutein at its S_0 minimum geometry (Table S12), computed with the R-AM1/FOMO-CASCI(6,6) method. These MOs were used as references in the construction of the diabatic states during the SH simulations.

#	Atomic orbital	Atom	Molecular orbital					
			HOMO-2	HOMO-1	HOMO	LUMO	LUMO+1	LUMO
1	S	C_1	0.01545	-0.01490	0.01105	-0.01035	-0.01301	0.01306
2	Px	C_1	-0.02412	0.02308	-0.01674	0.01551	0.01982	-0.02043
3	Py	C_1	-0.00620	0.00664	-0.00542	0.00577	0.00785	-0.00847
4	Pz	C_1	-0.06759	0.05623	-0.03791	0.03472	0.04601	-0.04993
5	S	C_2	-0.00133	0.00099	-0.00054	0.00020	0.00001	0.00020
6	Px	C_2	0.00322	-0.00297	0.00166	-0.00044	0.00034	-0.00117
7	Py	C_2	-0.00098	-0.00019	0.00049	-0.00055	-0.00065	0.00060
8	Pz	C_2	0.01533	-0.01017	0.00535	-0.00327	-0.00350	0.00316
9	S	C_3	0.00046	-0.00026	0.00012	-0.00006	-0.00006	0.00006
10	Px	C_3	-0.00100	0.00068	-0.00035	0.00017	0.00009	0.00022
11	Py	C_3	0.00572	-0.00351	0.00175	-0.00105	-0.00117	0.00118
12	Pz	C_3	-0.01390	0.00877	-0.00450	0.00289	0.00331	-0.00321
13	S	C_4	0.00141	-0.00103	0.00051	-0.00010	0.00016	-0.00042
14	Px	C_4	-0.00025	0.00095	-0.00046	-0.00048	-0.00144	0.00243
15	Py	C_4	-0.00578	0.00400	-0.00219	0.00118	0.00105	-0.00089
16	Pz	C_4	0.01188	-0.00743	0.00367	-0.00209	-0.00219	0.00193
17	S	C_5	-0.01693	0.01637	-0.01214	0.01143	0.01448	-0.01463
18	Px	C_5	0.02188	-0.02190	0.01588	-0.01435	-0.01762	0.01643
19	Py	C_5	0.02925	-0.02512	0.01795	-0.01761	-0.02431	0.02860
20	Pz	C_5	-0.03525	0.02874	-0.01884	0.01673	0.02184	-0.02304
21	S	C_6	0.00233	-0.00121	0.00026	0.00057	0.00128	-0.00184
22	Px	C_6	0.00385	-0.00312	0.00161	-0.00087	-0.00108	0.00203

23	Py	C_6	-0.01419	0.01289	-0.00809	0.00580	0.00677	-0.00785
24	Pz	C_6	0.05755	-0.04313	0.02451	-0.01297	-0.00893	0.00097
25	S	C_7	-0.00159	0.00135	-0.00093	0.00088	0.00120	-0.00135
26	Px	C_7	-0.00676	0.00634	-0.00469	0.00471	0.00636	-0.00698
27	Py	C_7	0.08025	-0.08190	0.06381	-0.06441	-0.08392	0.08646
28	Pz	C_7	-0.25778	0.26126	-0.20396	0.20704	0.27048	-0.27894
29	S	C_8	-0.00024	0.00011	-0.00003	-0.00008	-0.00022	0.00038
30	Px	C_8	-0.00309	0.00197	-0.00071	-0.00074	-0.00208	0.00332
31	Py	C_8	0.05842	-0.04056	0.01512	0.01354	0.03828	-0.05788
32	Pz	C_8	-0.20761	0.14490	-0.05487	-0.04549	-0.13134	0.19953
33	S	C_9	0.00026	-0.00025	0.00016	-0.00012	-0.00014	0.00013
34	Px	C_9	0.00328	-0.00513	0.00498	-0.00501	-0.00521	0.00345
35	Py	C_9	-0.04824	0.07904	-0.07856	0.07992	0.08299	-0.05394
36	Pz	C_9	0.16851	-0.27481	0.27268	-0.27720	-0.28784	0.18704
37	S	C_{10}	0.00014	-0.00014	0.00009	-0.00002	0.00001	-0.00002
38	Px	C_{10}	0.00581	-0.00524	0.00232	0.00187	0.00477	-0.00571
39	Py	C_{10}	-0.08709	0.07902	-0.03491	-0.02962	-0.07410	0.08807
40	Pz	C_{10}	0.30409	-0.27633	0.12254	0.10258	0.25778	-0.30674
41	S	C_{11}	-0.00006	0.00003	-0.00001	0.00001	0.00002	-0.00002
42	Px	C_{11}	0.00130	0.00292	-0.00539	0.00551	0.00323	0.00117

Table S11: (continued from previous page)

#	Atomic orbital	Atom	Molecular orbital					
			HOMO-2	HOMO-1	HOMO	LUMO	LUMO+1	LUMO
43	Py	C_{11}	-0.01887	-0.04281	0.07899	-0.08043	-0.04654	-0.01831
44	Pz	C_{11}	0.06605	0.14965	-0.27643	0.28195	0.16358	0.06352
45	S	C_{12}	0.00009	0.00002	-0.00008	0.00002	-0.00001	-0.00001
46	Px	C_{12}	-0.00457	0.00620	-0.00314	-0.00332	-0.00614	0.00430
47	Py	C_{12}	0.06270	-0.08815	0.04553	0.04762	0.08798	-0.06056
48	Pz	C_{12}	-0.22012	0.31034	-0.16087	-0.16705	-0.30952	0.21339
49	S	C_{13}	0.00005	-0.00007	0.00000	0.00002	-0.00003	0.00002
50	Px	C_{13}	-0.00575	0.00022	0.00620	-0.00635	-0.00005	-0.00600
51	Py	C_{13}	0.07938	-0.00194	-0.08533	0.08591	-0.00003	0.08227
52	Pz	C_{13}	-0.28072	0.00732	0.30145	-0.30425	-0.00028	-0.29101
53	S	C_{14}	-0.00007	0.00003	0.00004	-0.00004	-0.00001	0.00001
54	Px	C_{14}	-0.00166	-0.00541	0.00500	0.00449	0.00562	0.00104
55	Py	C_{14}	0.01903	0.07015	-0.06302	-0.05923	-0.07232	-0.01429
56	Pz	C_{14}	-0.06938	-0.24982	0.22598	0.21044	0.25827	0.05038
57	S	C_{15}	0.00007	0.00006	-0.00005	-0.00002	0.00002	0.00000
58	Px	C_{15}	0.00572	-0.00351	-0.00604	0.00624	-0.00332	0.00607
59	Py	C_{15}	-0.06934	0.04231	0.07397	-0.07479	0.04148	-0.07349
60	Pz	C_{15}	0.25000	-0.15332	-0.26615	0.27044	-0.14904	0.26538
61	S	C_{16}	0.00045	-0.00138	0.00138	-0.00164	-0.00239	0.00291
62	Px	C_{16}	-0.01217	0.00728	-0.00239	-0.00219	-0.00609	0.00955
63	Py	C_{16}	-0.00179	0.00298	-0.00281	0.00358	0.00549	-0.00688
64	Pz	C_{16}	0.01044	-0.00558	0.00186	0.00081	0.00278	-0.00475
65	S	C_{17}	0.00511	-0.00590	0.00485	-0.00490	-0.00631	0.00639

66	Px	C_{17}	-0.02461	0.02169	-0.01455	0.01162	0.01346	-0.01256
67	Py	C_{17}	-0.00575	0.00291	-0.00053	-0.00145	-0.00310	0.00425
68	Pz	C_{17}	0.00457	-0.00122	-0.00064	0.00182	0.00275	-0.00302
69	S	C_{18}	-0.00332	0.00445	-0.00386	0.00412	0.00552	-0.00593
70	Px	C_{18}	0.01549	-0.01090	0.00567	-0.00222	-0.00076	-0.00105
71	Py	C_{18}	0.01065	-0.01121	0.00857	-0.00825	-0.01052	0.01054
72	Pz	C_{18}	0.00794	-0.00308	-0.00009	0.00269	0.00521	-0.00722
73	S	C_{19}	0.00006	-0.00010	0.00008	-0.00004	-0.00003	0.00002
74	Px	C_{19}	-0.00016	0.00049	-0.00051	0.00042	0.00035	-0.00013
75	Py	C_{19}	0.00859	-0.01433	0.01289	-0.01072	-0.01005	0.00602
76	Pz	C_{19}	-0.03073	0.05034	-0.04506	0.03756	0.03549	-0.02164
77	S	C_{20}	-0.00001	-0.00011	0.00010	0.00005	0.00008	-0.00002
78	Px	C_{20}	0.00154	-0.00026	-0.00085	0.00074	0.00009	0.00047
79	Py	C_{20}	-0.02151	0.00344	0.01332	-0.01188	-0.00076	-0.00826
80	Pz	C_{20}	0.07599	-0.01288	-0.04649	0.04255	0.00348	0.02913
81	S	O_{21}	-0.00072	0.00037	-0.00017	0.00012	0.00016	-0.00022
82	Px	O_{21}	0.00025	-0.00003	-0.00002	0.00001	-0.00004	0.00022
83	Py	O_{21}	-0.00195	0.00090	-0.00034	0.00015	0.00014	-0.00008
84	Pz	O_{21}	0.00075	-0.00020	-0.00001	0.00009	0.00016	-0.00033
85	S	C_{22}	-0.01789	-0.01628	-0.01171	-0.01091	0.01365	0.01357
86	Px	C_{22}	-0.03614	-0.03208	-0.02236	-0.02051	0.02591	0.02555

Table S11: (continued from previous page)

#	Atomic orbital	Atom	Molecular orbital					
			HOMO-2	HOMO-1	HOMO	LUMO	LUMO+1	LUMO
87	Py	C_{22}	0.01244	0.00812	0.00480	0.00419	-0.00574	-0.00673
88	Pz	C_{22}	-0.07328	-0.05777	-0.03780	-0.03454	0.04555	0.04861
89	S	C_{23}	0.00338	0.00197	0.00101	0.00057	-0.00054	-0.00058
90	Px	C_{23}	-0.00111	0.00239	0.00188	0.00097	-0.00043	0.00034
91	Py	C_{23}	0.00008	-0.00040	-0.00043	-0.00079	0.00147	0.00217
92	Pz	C_{23}	0.02113	0.01214	0.00596	0.00353	-0.00373	-0.00324
93	S	C_{24}	-0.00141	-0.00094	-0.00056	-0.00053	0.00090	0.00170
94	Px	C_{24}	0.00927	0.00385	0.00191	0.00193	-0.00367	-0.00777
95	Py	C_{24}	-0.01029	-0.00355	-0.00121	-0.00010	-0.00095	-0.00395
96	Pz	C_{24}	-0.01898	-0.01103	-0.00536	-0.00331	0.00374	0.00374
97	S	C_{25}	0.00012	0.00008	0.00030	0.00079	-0.00145	-0.00197
98	Px	C_{25}	-0.00958	-0.00476	-0.00211	-0.00059	0.00006	-0.00002
99	Py	C_{25}	0.06499	0.02905	0.01465	0.01244	-0.01865	-0.02736
100	Pz	C_{25}	0.01547	0.00966	0.00485	0.00306	-0.00354	-0.00381
101	S	C_{26}	0.01450	0.01453	0.01086	0.01054	-0.01343	-0.01339
102	Px	C_{26}	0.02994	0.02919	0.02154	0.02147	-0.02875	-0.03152
103	Py	C_{26}	0.05156	0.01751	0.00518	-0.00162	0.00787	0.01926
104	Pz	C_{26}	-0.04506	-0.03473	-0.02206	-0.01942	0.02525	0.02664
105	S	C_{27}	-0.00188	-0.00089	-0.00019	0.00037	-0.00082	-0.00107
106	Px	C_{27}	0.00617	0.00375	0.00171	0.00032	0.00060	0.00176
107	Py	C_{27}	-0.02878	-0.01602	-0.00837	-0.00463	0.00359	0.00086
108	Pz	C_{27}	0.06607	0.04816	0.02666	0.01408	-0.00983	-0.00205
109	S	C_{28}	-0.00221	-0.00066	-0.00022	-0.00001	-0.00018	-0.00054

110	Px	C_{28}	-0.00322	-0.00610	-0.00533	-0.00608	0.00852	0.00963
111	Py	C_{28}	0.06938	0.06658	0.05124	0.05258	-0.06834	-0.06916
112	Pz	C_{28}	-0.26007	-0.26419	-0.20540	-0.21189	0.27651	0.28187
113	S	C_{29}	-0.00059	-0.00020	-0.00009	-0.00003	-0.00003	-0.00011
114	Px	C_{29}	-0.00560	-0.00418	-0.00171	0.00100	-0.00318	-0.00472
115	Py	C_{29}	0.05341	0.03672	0.01367	-0.01188	0.03393	0.05089
116	Pz	C_{29}	-0.21066	-0.14304	-0.05180	0.05006	-0.13863	-0.20604
117	S	C_{30}	-0.00010	0.00004	0.00002	0.00002	-0.00009	-0.00019
118	Px	C_{30}	0.00526	0.00772	0.00747	0.00751	-0.00754	-0.00440
119	Py	C_{30}	-0.04268	-0.07102	-0.06999	-0.07179	0.07392	0.04667
120	Pz	C_{30}	0.16926	0.27884	0.27429	0.28062	-0.28825	-0.18086
121	S	C_{31}	-0.00001	0.00008	0.00011	0.00011	-0.00014	-0.00016
122	Px	C_{31}	0.00811	0.00733	0.00311	-0.00311	0.00743	0.00861
123	Py	C_{31}	-0.07899	-0.07136	-0.03105	0.02765	-0.06813	-0.07932
124	Pz	C_{31}	0.30582	0.27555	0.11901	-0.10836	0.26486	0.30743
125	S	C_{32}	-0.00021	-0.00008	0.00004	0.00007	-0.00002	0.00004
126	Px	C_{32}	0.00180	-0.00407	-0.00731	-0.00745	0.00422	-0.00182
127	Py	C_{32}	-0.01759	0.04006	0.07268	0.07428	-0.04194	0.01839
128	Pz	C_{32}	0.06706	-0.15395	-0.27836	-0.28317	0.15885	-0.07141
129	S	C_{33}	0.00020	0.00001	-0.00011	-0.00005	0.00002	0.00006
130	Px	C_{33}	-0.00566	-0.00828	-0.00430	0.00462	-0.00833	-0.00539

Table S11: (continued from previous page)

#	Atomic orbital	Atom	Molecular orbital					
			HOMO-2	HOMO-1	HOMO	LUMO	LUMO+1	LUMO
131	Py	C_{33}	0.05785	0.08224	0.04209	-0.04522	0.08257	0.05497
132	Pz	C_{33}	-0.22053	-0.31087	-0.15752	0.17243	-0.31260	-0.20727
133	S	C_{34}	0.00004	0.00009	0.00002	-0.00002	-0.00003	-0.00004
134	Px	C_{34}	-0.00753	-0.00004	0.00806	0.00795	0.00027	0.00781
135	Py	C_{34}	0.07608	0.00111	-0.08166	-0.08194	-0.00184	-0.07926
136	Pz	C_{34}	-0.28178	-0.00256	0.30394	0.30345	0.00763	0.29437
137	S	C_{35}	-0.00016	-0.00011	0.00003	0.00009	-0.00009	-0.00004
138	Px	C_{35}	-0.00132	0.00634	0.00533	-0.00547	0.00635	-0.00146
139	Py	C_{35}	0.01960	-0.06882	-0.06134	0.05821	-0.06990	0.01576
140	Pz	C_{35}	-0.07007	0.25227	0.22309	-0.21432	0.25568	-0.05862
141	S	C_{36}	0.00012	-0.00008	-0.00006	0.00001	0.00004	-0.00002
142	Px	C_{36}	0.00563	0.00337	-0.00613	-0.00601	-0.00365	-0.00594
143	Py	C_{36}	-0.06892	-0.04145	0.07401	0.07414	0.04278	0.07220
144	Pz	C_{36}	0.24976	0.14954	-0.26879	-0.26798	-0.15565	-0.26129
145	S	C_{37}	-0.00283	-0.00401	-0.00364	-0.00427	0.00590	0.00640
146	Px	C_{37}	-0.00946	-0.01112	-0.00887	-0.00891	0.01143	0.01159
147	Py	C_{37}	-0.00838	-0.00338	-0.00045	0.00205	-0.00437	-0.00630
148	Pz	C_{37}	0.00646	0.00449	0.00227	0.00130	-0.00133	-0.00108
149	S	C_{38}	-0.00246	-0.00334	-0.00278	-0.00280	0.00365	0.00386
150	Px	C_{38}	-0.02123	-0.01475	-0.00721	-0.00177	-0.00132	-0.00467
151	Py	C_{38}	-0.01138	-0.01000	-0.00656	-0.00531	0.00636	0.00629
152	Pz	C_{38}	0.00894	0.00311	-0.00027	-0.00293	0.00545	0.00726
153	S	C_{39}	0.00199	0.00372	0.00352	0.00399	-0.00534	-0.00558

154	Px	C_{39}	0.01895	0.01474	0.00872	0.00545	-0.00496	-0.00315
155	Py	C_{39}	-0.00399	0.00375	0.00423	0.00490	-0.00677	-0.00788
156	Pz	C_{39}	0.00998	0.00333	-0.00037	-0.00304	0.00541	0.00683
157	S	C_{40}	0.00007	0.00008	0.00002	-0.00007	0.00013	0.00014
158	Px	C_{40}	-0.00060	-0.00133	-0.00127	-0.00115	0.00108	0.00059
159	Py	C_{40}	0.00749	0.01285	0.01149	0.00980	-0.00933	-0.00568
160	Pz	C_{40}	-0.03072	-0.05120	-0.04540	-0.03804	0.03554	0.02095
161	S	C_{41}	0.00008	0.00024	0.00013	-0.00014	0.00017	0.00004
162	Px	C_{41}	0.00201	0.00034	-0.00136	-0.00129	0.00002	-0.00097
163	Py	C_{41}	-0.02064	-0.00368	0.01232	0.01170	-0.00113	0.00779
164	Pz	C_{41}	0.07621	0.01176	-0.04713	-0.04242	0.00244	-0.02955
165	S	O_{42}	0.00166	0.00095	0.00054	0.00057	-0.00108	-0.00225
166	Px	O_{42}	0.00554	0.00244	0.00125	0.00123	-0.00230	-0.00471
167	Py	O_{42}	0.01419	0.00515	0.00219	0.00174	-0.00300	-0.00584
168	Pz	O_{42}	-0.00461	-0.00202	-0.00117	-0.00134	0.00264	0.00569
169	S	H_{43}	-0.00381	0.00270	-0.00157	0.00114	0.00132	-0.00124
170	S	H_{44}	0.01292	-0.00915	0.00517	-0.00367	-0.00439	0.00443
171	S	H_{45}	-0.00108	0.00084	-0.00050	0.00039	0.00047	-0.00043
172	S	H_{46}	-0.00276	0.00219	-0.00122	0.00082	0.00090	-0.00067
173	S	H_{47}	-0.00770	0.00517	-0.00293	0.00224	0.00289	-0.00332

Table S11: (continued from previous page)

#	Atomic orbital	Atom	Molecular orbital					
			HOMO-2	HOMO-1	HOMO	LUMO	LUMO+1	LUMO
174	S	H_{48}	-0.00137	0.00139	-0.00078	0.00039	0.00041	-0.00059
175	S	H_{49}	-0.00124	0.00116	-0.00077	0.00063	0.00075	-0.00072
176	S	H_{50}	0.00007	-0.00015	0.00017	-0.00015	-0.00014	0.00007
177	S	H_{51}	-0.00009	0.00011	-0.00005	-0.00008	-0.00014	0.00011
178	S	H_{52}	0.00014	0.00005	-0.00018	0.00013	0.00001	0.00009
179	S	H_{53}	-0.00037	0.00012	0.00029	-0.00027	0.00006	-0.00022
180	S	H_{54}	0.00005	0.00020	-0.00018	-0.00017	-0.00016	-0.00008
181	S	H_{55}	0.00108	0.00025	-0.00091	0.00125	0.00135	-0.00080
182	S	H_{56}	-0.00350	0.00206	-0.00088	0.00031	0.00021	-0.00004
183	S	H_{57}	0.00781	-0.00582	0.00363	-0.00308	-0.00397	0.00421
184	S	H_{58}	0.00157	-0.00031	-0.00011	-0.00007	-0.00046	0.00091
185	S	H_{59}	-0.00316	0.00116	0.00015	-0.00110	-0.00178	0.00200
186	S	H_{60}	0.01139	-0.00931	0.00576	-0.00406	-0.00448	0.00406
187	S	H_{61}	-0.00563	0.00421	-0.00271	0.00248	0.00349	-0.00423
188	S	H_{62}	-0.00746	0.00657	-0.00416	0.00277	0.00269	-0.00188
189	S	H_{63}	0.00159	-0.00160	0.00100	-0.00037	0.00003	-0.00050
190	S	H_{64}	-0.00023	0.00033	-0.00029	0.00026	0.00027	-0.00021
191	S	H_{65}	-0.02825	0.04300	-0.03837	0.03330	0.03247	-0.02037
192	S	H_{66}	0.02872	-0.04357	0.03881	-0.03366	-0.03284	0.02065
193	S	H_{67}	0.00017	-0.00002	-0.00015	0.00015	0.00001	0.00012
194	S	H_{68}	0.05392	-0.00356	-0.04207	0.03665	0.00040	0.03074
195	S	H_{69}	-0.05413	0.00357	0.04226	-0.03682	-0.00039	-0.03088
196	S	H_{70}	0.00156	-0.00089	0.00045	-0.00033	-0.00044	0.00055

197	S	H_{71}	0.00283	0.00251	0.00156	0.00124	-0.00156	-0.00185
198	S	H_{72}	-0.01370	-0.00947	-0.00522	-0.00366	0.00426	0.00401
199	S	H_{73}	-0.01035	-0.00355	-0.00142	-0.00094	0.00141	0.00232
200	S	H_{74}	0.01262	0.00825	0.00456	0.00345	-0.00436	-0.00484
201	S	H_{75}	0.01943	0.01117	0.00638	0.00480	-0.00537	-0.00424
202	S	H_{76}	0.00201	0.00052	0.00021	0.00018	-0.00026	-0.00032
203	S	H_{77}	-0.00014	-0.00052	-0.00043	-0.00037	0.00040	0.00032
204	S	H_{78}	0.00019	0.00034	0.00036	0.00030	-0.00020	-0.00000
205	S	H_{79}	-0.00025	-0.00035	-0.00020	0.00011	-0.00027	-0.00025
206	S	H_{80}	0.00039	-0.00011	-0.00045	-0.00031	-0.00001	-0.00025
207	S	H_{81}	-0.00049	-0.00010	0.00040	0.00034	0.00006	0.00026
208	S	H_{82}	0.00006	-0.00020	-0.00018	0.00017	-0.00016	0.00007
209	S	H_{83}	-0.00624	-0.00373	-0.00193	-0.00161	0.00228	0.00264
210	S	H_{84}	-0.00511	-0.00449	-0.00292	-0.00215	0.00238	0.00222
211	S	H_{85}	0.00460	0.00284	0.00116	0.00012	0.00031	0.00065
212	S	H_{86}	-0.00571	-0.00338	-0.00125	-0.00011	-0.00004	0.00016
213	S	H_{87}	-0.00666	-0.00470	-0.00290	-0.00268	0.00362	0.00396
214	S	H_{88}	0.00392	0.00235	0.00100	0.00034	-0.00022	-0.00012
215	S	H_{89}	0.00143	-0.00116	-0.00132	-0.00113	0.00116	0.00099
216	S	H_{90}	-0.00071	0.00504	0.00458	0.00460	-0.00609	-0.00706

Table S11: (continued from previous page)

#	Atomic orbital	Atom	Molecular orbital					
			HOMO-2	HOMO-1	HOMO	LUMO	LUMO+1	LUMO
217	S	H_{91}	0.01145	0.00612	0.00328	0.00224	-0.00232	-0.00144
218	S	H_{92}	-0.02836	-0.04348	-0.03840	-0.03353	0.03238	0.01968
219	S	H_{93}	0.02828	0.04364	0.03858	0.03368	-0.03248	-0.01968
220	S	H_{94}	-0.00009	-0.00015	-0.00015	-0.00013	0.00010	0.00001
221	S	H_{95}	-0.00016	-0.00005	0.00004	0.00006	-0.00002	0.00003
222	S	H_{96}	0.05414	0.00284	-0.04239	-0.03657	-0.00044	-0.03107
223	S	H_{97}	-0.05396	-0.00278	0.04237	0.03650	0.00051	0.03108
224	S	H_{98}	-0.00306	-0.00168	-0.00090	-0.00088	0.00158	0.00315

Table S12: Cartesian coordinates (Å) for the S_0 minimum geometry of lutein, computed with the R-AM1/FOMO-CASCI(6,6) method.

Atom	Coordinate		
	X	Y	Z
C_1	12.147412	-0.081937	1.138385
C_2	13.593306	0.323717	1.139579
C_3	13.873314	1.521556	0.331927
C_4	13.393161	1.406240	-1.060732
C_5	12.163146	0.657812	-1.231539
C_6	11.600441	-0.037040	-0.233428
C_7	10.426492	-0.807104	-0.450037
C_8	9.165296	-0.436204	-0.320012
C_9	7.985058	-1.187987	-0.518167
C_{10}	6.763119	-0.629252	-0.335473

C_{11}	5.493345	-1.163496	-0.464329
C_{12}	4.320490	-0.549236	-0.266255
C_{13}	3.011026	-1.061472	-0.384724
C_{14}	1.932665	-0.277496	-0.141365
C_{15}	0.582981	-0.556963	-0.189369
C_{16}	11.355136	0.852458	1.995835
C_{17}	12.035193	-1.466035	1.695297
C_{18}	11.567265	0.689131	-2.550209
C_{19}	8.159042	-2.567646	-0.919765
C_{20}	2.886755	-2.450431	-0.775062
O_{21}	15.355051	1.769943	0.231318
C_{22}	-12.162351	-0.047578	-1.117819
C_{23}	-13.466905	-0.790176	-1.168089
C_{24}	-14.359893	-0.457705	-0.042696
C_{25}	-13.678273	-0.412773	1.231260
C_{26}	-12.365365	-0.313620	1.371979
C_{27}	-11.476004	-0.344172	0.201588
C_{28}	-10.291183	0.474715	0.378099
C_{29}	-9.027821	0.111972	0.253293

Table S12: (continued from previous page)

Atom	Coordinate		
	X	Y	Z
C_{30}	-7.852210	0.880429	0.415831
C_{31}	-6.627220	0.325409	0.239968
C_{32}	-5.360196	0.870335	0.347541
C_{33}	-4.185153	0.258161	0.155271
C_{34}	-2.877239	0.777758	0.259572
C_{35}	-1.797351	-0.004661	0.019802
C_{36}	-0.447829	0.277328	0.064959
C_{37}	-12.416651	1.416687	-1.245263
C_{38}	-11.298474	-0.492206	-2.250274
C_{39}	-11.710808	-0.212122	2.656372
C_{40}	-8.031820	2.270847	0.774888
C_{41}	-2.756967	2.171910	0.632288
O_{42}	-15.478506	-1.460270	0.083462
H_{43}	14.220667	-0.488510	0.746931
H_{44}	13.958423	0.473147	2.162621
H_{45}	13.492938	2.418937	0.812902
H_{46}	14.183494	0.920528	-1.655022
H_{47}	13.304915	2.408758	-1.500194
H_{48}	10.666671	-1.800574	-0.767272
H_{49}	9.010469	0.583578	-0.024920
H_{50}	6.758126	0.403243	-0.039310
H_{51}	5.453910	-2.190779	-0.757093
H_{52}	4.378671	0.480616	0.025345

H_{53}	2.142354	0.738432	0.138867
H_{54}	0.312697	-1.555917	-0.460230
H_{55}	10.299186	0.576592	2.050235
H_{56}	11.379747	1.881910	1.634190
H_{57}	11.714134	0.877840	3.026784
H_{58}	12.512383	-1.555276	2.674651
H_{59}	12.508180	-2.213295	1.055109
H_{60}	11.002619	-1.791838	1.838118
H_{61}	10.662003	1.303314	-2.552448
H_{62}	11.274439	-0.304184	-2.904665
H_{63}	12.214903	1.099661	-3.330694
H_{64}	7.241865	-3.141204	-1.064398
H_{65}	8.737090	-3.130049	-0.179362
H_{66}	8.698671	-2.642000	-1.869451
H_{67}	1.867583	-2.832015	-0.859241
H_{68}	3.383534	-3.113564	-0.059699
H_{69}	3.343709	-2.633174	-1.752870
H_{70}	15.761107	1.791855	1.106910
H_{71}	-13.291440	-1.874046	-1.147471
H_{72}	-13.982608	-0.618938	-2.119825

Table S12: (continued from previous page)

Atom	Coordinate		
	X	Y	Z
H_{73}	-14.883310	0.484839	-0.206412
H_{74}	-14.334505	-0.472138	2.069256
H_{75}	-11.178211	-1.410006	0.172105
H_{76}	-10.517171	1.486058	0.636853
H_{77}	-8.860302	-0.915098	-0.007476
H_{78}	-6.616849	-0.714690	-0.028250
H_{79}	-5.324297	1.903907	0.617758
H_{80}	-4.239781	-0.777290	-0.116645
H_{81}	-2.004900	-1.023092	-0.253019
H_{82}	-0.178359	1.276764	0.334684
H_{83}	-13.072173	1.655937	-2.086865
H_{84}	-11.506972	1.998062	-1.411819
H_{85}	-12.891402	1.839615	-0.357707
H_{86}	-10.312977	-0.021171	-2.236158
H_{87}	-11.735409	-0.258990	-3.223940
H_{88}	-11.119352	-1.569512	-2.247214
H_{89}	-12.350788	-0.473512	3.504797
H_{90}	-11.366562	0.809556	2.842353
H_{91}	-10.834958	-0.862264	2.736872
H_{92}	-8.571664	2.373437	1.721858
H_{93}	-8.611929	2.807444	0.017082
H_{94}	-7.117064	2.852577	0.901553
H_{95}	-1.738829	2.558262	0.707209

H_{96}	-3.210667	2.365471	1.609533
H_{97}	-3.259142	2.824038	-0.089454
H_{98}	-15.897508	-1.608951	-0.774215

S5 Dynamics simulations in vacuum

To assess the effect of the environment in the QM/MM simulations in methanol solution, we performed simulations of the same kind for the isolated lutein. The initial conditions for the surface hopping (SH) simulations were sampled from a classical thermal equilibration in the ground state (S_0), propagated for 10 ps using the Bussi-Parrinello thermostat[15,16]. The selection of initial conditions for the SH dynamics was performed using an excitation energy interval of 2.6 $\pm$ 0.1 eV, and taking into account the radiative transition probability from S_0[17]. A total of 201 SH trajectories (187 starting from S_2 and 14 from S_3) were propagated for 200 fs, using the same propagation algorithm and decoherence correction employed for the simulations in methanol (see Methods in the main text).

To analyse the nature of the electronic states during the nonadiabatic dynamics, we define diabatic state populations in the same way as we did for the simulations in methanol (see Section S2). The obtained adiabatic and diabatic populations, as functions of time, are reported in Figure S12. As for the simulations in methanol, the lifetimes of the $1B_u^+$ and $1B_u^-$ states were determined by fitting the diabatic populations using the rate model described in Section S3.3.

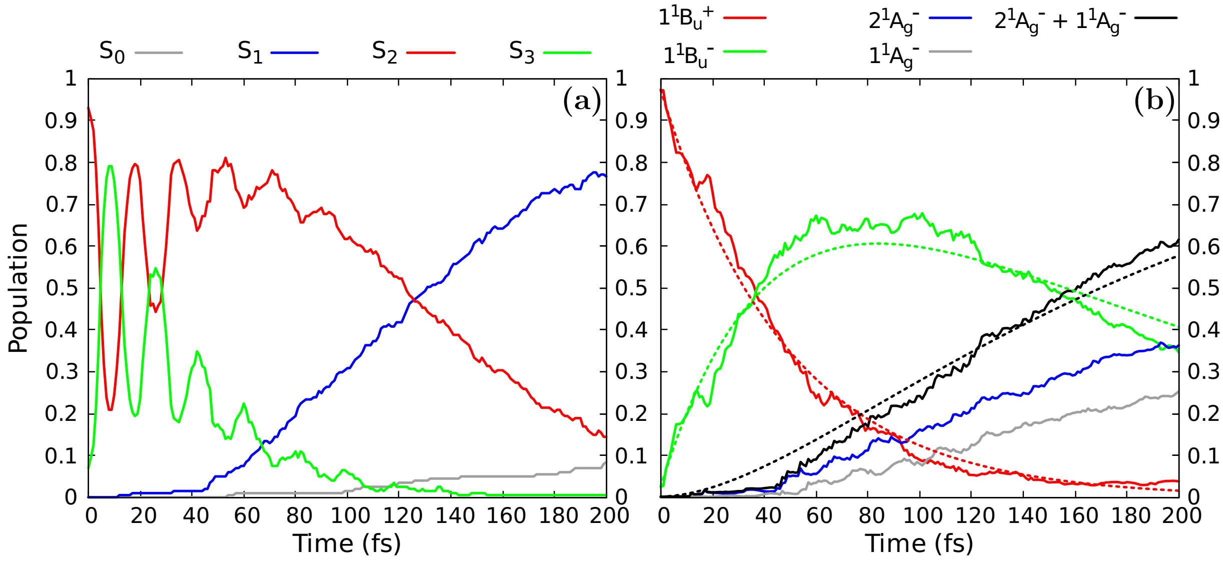

Figure S12: Adiabatic (panel a) and diabatic (panel b) state populations as functions of time obtained from the simulations of excited state dynamics for lutein in vacuum. The reported results are obtained by averaging over all trajectories and time intervals of 1 fs. In panel b, the fitting functions for the diabatic populations are also shown (dashed lines, see Eq. S7-S9 in Section S3.3). The extracted time constants are τ_{2x} = 48.5 fs and τ_{x1} = 170.6 fs.

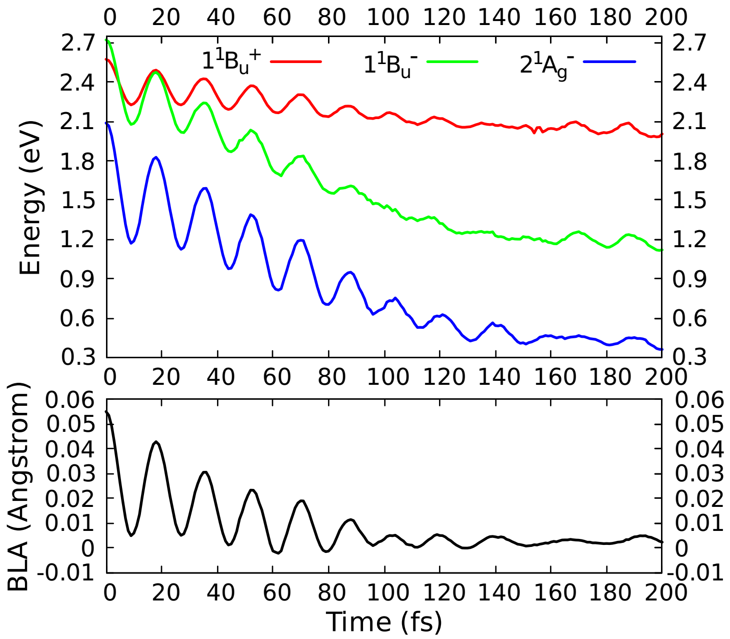

Figure S13: Energies (eV) of the three low-lying diabatic excited states relative to the diabatic ground state ($1A_g^-$) and bond length alternation (BLA, Å) as functions of time. The reported results are obtained by averaging over all trajectories and time intervals of 1 fs.

Table S13: Comparison of the energy gaps, electronic couplings and BLA values at $t = 0$ and at the transitions between pairs of states ($i \to j$), averaged over all SH trajectories. The average times (in fs) for the selected geometries (X) are also given. The energy gaps and electronic couplings are reported in units of meV, while the BLA values are in Å. For the electronic couplings, the mean absolute values are reported.

States		Geom.	Energy gap	coupling	BLA	Time
$1B_u^+$	$1B_u^-$	X_0	153.3	7.9	0.0553	0
		$X_{i\to j}$	-19.8	5.8	0.0291	70
$1B_u^-$	$1B_u^+$	X_0	-153.3	7.9	0.0553	0
		$X_{i\to j}$	20.1	5.5	0.0309	63
$1B_u^-$	$2A_g^-$	X_0	-635.1	65.3	0.0553	0
		$X_{i\to j}$	-547.1	204.5	0.0085	160
$1B_u^-$	$1A_g^-$	X_0	-635.1	65.3	0.0553	0
		$X_{i\to j}$	-343.8	48.9	-0.0062	189

References

(1) Nelder, J. A.; Mead, R. A Simplex Method for Function Minimization. *Comput. J.* **1965**, *7*, 308–313.

(2) Press, W. H.; Teukolsky, S. A.; Vetterling, W. T.; Flannery, B. P. *Numerical Recipes in Fortran 77; Cambridge U. P.* **2001**,

(3) Josue, J. S.; Frank, H. A. Direct Determination of the S1 Excited-State Energies of Xanthophylls by Low-Temperature Fluorescence Spectroscopy. *J. Phys. Chem. A* **2002**, *106*, 4815–4824.

(4) Dewar, M. J. S.; Zoebisch, E. G.; Healy, E. F.; Stewart, J. J. P. AM1: A New General Purpose Quantum Mechanical Molecular Model. *J. Am. Chem. Soc.* **1985**, *107*, 3902–3909.

(5) Winget, P.; Selçuki, C.; Horn, A. H. C.; Martin, B.; Clark, T. Towards a "Next Generation" Neglect of Diatomic Differential Overlap Based Semiempirical Molecular Orbital Technique. *Theor. Chem. Acc.* **2003**, *110*, 254–266.

(6) Zsila, F.; Bikádi, Z.; Keresztes, Z.; Deli, J.; Simonyi, M. Investigation of the Self-Organization of Lutein and Lutein Diacetate by Electronic Absorption, Circular Dichroism Spectroscopy, and Atomic Force Microscopy. *J. Phys. Chem. B* **2001**, *105*, 9413–9421.

(7) Accomasso, D.; Persico, M.; Granucci, G. Diabatization by Localization in the Framework of Configuration Interaction Based on Floating Occupation Molecular Orbitals (FOMO-CI). *ChemPhotoChem.* **2019**, *3*, 933–944.

(8) Miki, T.; Buckup, T.; Krause, M. S.; Southall, J.; Cogdell, R. J.; Motzkus, M. Vibronic Coupling in the Excited-States of Carotenoids. *Phys. Chem. Chem. Phys.* **2016**, *18*, 11443–11453.

(9) Ostroumov, E.; Müller, M. G.; Marian, C. M.; Kleinschmidt, M.; Holzwarth, A. R. Electronic Coherence Provides a Direct Proof for Energy-Level Crossing in Photoexcited Lutein and β-Carotene. *Phys. Rev. Lett.* **2009**, *103*, 108302.

(10) Polli, D.; Cerullo, G.; Lanzani, G.; De Silvestri, S.; Yanagi, K.; Hashimoto, H.; Cogdell, R. J. Conjugation Length Dependence of Internal Conversion in Carotenoids: Role of the Intermediate State. *Phys. Rev. Lett.* **2004**, *93*, 163002.

(11) Zhang, J.-P.; Inaba, T.; Watanabe, Y.; Koyama, Y. Excited-State Dynamics Among the $1B_u^+$, $1B_u^-$ and $2A_g^-$ States of All-Trans-Neurosporene as Revealed by Near-Infrared Time-Resolved Absorption Spectroscopy. *Chem. Phys. Lett.* **2000**, *332*, 351–358.

(12) Maiuri, M.; Polli, D.; Brida, D.; Lüer, L.; LaFountain, A. M.; Fuciman, M.; Cogdell, R. J.; Frank, H. A.; Cerullo, G. Solvent-Dependent Activation of Intermediate Excited States in the Energy Relaxation Pathways of Spheroidene. *Phys. Chem. Chem. Phys.* **2012**, *14*, 6312–6319.

(13) Cerullo, G.; Polli, D.; Lanzani, G.; De Silvestri, S.; Hashimoto, H.; Cogdell, R. J. Photosynthetic Light Harvesting by Carotenoids: Detection of an Intermediate Excited State. *Science* **2002**, *298*, 2395–2398.

(14) Marek, M. S.; Buckup, T.; Motzkus, M. Direct Observation of a Dark State in Lycopene Using Pump-DFWM. *J. Phys. Chem. B* **2011**, *115*, 8328–8337.

(15) Bussi, G.; Donadio, D.; Parrinello, M. Canonical Sampling through Velocity Rescaling. *J. Chem. Phys.* **2007**, *126*, 014101.

(16) Bussi, G.; M., P. Stochastic Thermostats: Comparison of Local and Global Schemes. *Comp. Phys. Commun.* **2008**, *179*, 26–29.

(17) Persico, M.; Granucci, G. An Overview of Nonadiabatic Dynamics Simulations Methods, with Focus on the Direct Approach Versus the Fitting of Potential Energy Surfaces. *Theor. Chem. Acc.* **2014**, *133*, 1526.
