# Supplementary material for: Ultrafast Excited-State Dynamics of Carotenoids and the Role of the SX State
Source: J Phys Chem Lett. 2022 Jul 19;13(29):6762–9. doi: 10.1021/acs.jpclett.2c01555 (PMC9340805; doi:10.1021/acs.jpclett.2c01555)
Supplement: Supplementary file 2 — jz2c01555_si_002.pdf [file jz2c01555_si_002.pdf]

jz-2022-015559.R1

Name: Peer Review Information for "The Ultrafast Excited State Dynamics of Carotenoids and the Role of the Sx State"

First Round of Reviewer Comments

Reviewer: 1

Comments to the Author

In this work the authors performed nonadiabatic excited-state dynamics calculations on lutein, a xanthophyll carotenoid found in the LHCII complex of green plants. Understanding the excited state dynamics of carotenoids is hindered by the presence of a debated number of optically dark states. Using mixed quantum-classical surface hopping calculations, Mennucci and coworkers identified the nature of one of the dark states, SX, as formed through a change in S2 character on the ~20 fs timescale, and furthermore identified the role of the SX state in mediating S2-S1 conversion on the sub-ps timescale. The work presented here will be of interest those interested in natural light-harvesting and exciton dynamics, and thus should be published in The Journal of Physical Chemistry Letters after the following comments are addressed.

Major Comments:

(1) The authors model lutein dynamics in the case of methanol and in the gas phase, however they also point to implications for light-harvesting. The authors should discuss how lutein dynamics would vary in this case according to (1) polarity conditions of the membrane protein environment (such as LHCII), and (2) variation in lutein structure within the protein pocket (thus giving rise to lutein 1 and lutein 2 structures).

(2) On page 11, the authors note that a variation in potential energy surface curvature results in an increase in the C=C stretching mode of the S1 state. Is this trend reflective of the BLA frequency after the S1 state is formed (beyond the 200 fs timescale)?

(3) On page 13 the authors note, “Our simulations finally showed that high-frequency BLA modes are fundamental in driving the transition to the 1Bu−/SX state” However the BLA modes are on the order of 1700 cm-1, not the experimentally observed 1100-1500cm-1 (Llansola-Portoles et al. J. R. Soc. Interface, 2017, 14). The authors should address this discrepancy, and comment on how dynamics might change under the influence of lower frequency BLA oscillations.

(4) According to Table 1, the 1Bu- state appears to have a non-zero oscillator strength in lutein, in contrast to the C20 polyene. The authors should comment on the origin of this deviation from total dark state character.

(5) As discussed in reference 51, The Sx state is assigned through a torsional motion to the S2 state. While this bond torsion is not observed, a partial intramolecular charge-transfer state is also implicated

in reference 51- do this authors observe significant redistribution of electron density to support the potential impact of an intramolecular charge-transfer state?

Reviewer: 2

Comments to the Author

Accomasso et al. present on the fly mixed quantum-classical dynamics simulation of the carotenoid lutein in methanol. The electronic structure of the solute is computed at the semi-empirical level, reparamterized to reproduce a number of parameters (transition energies, TDMs, geometircal parameters) whereas the interaction with the solvent is treated within a QMMM framework. The semi-emprical calculations allow to run a comparably big number 200 trajectories and collect reliable statistics.

The main finding of this work which represents one of the first dynamical studies on carotenes, is the involvement of a higher lying spectroscopically dark state (1Bu-) besides the usual suspects 2Ag- (the lowest lying dark hh->l,l state) and 1Bu+ (the lowest bright state with h->l character). It is found that this state exhibits a more pronounced stabilization along the BLA coordinate wrt the 1Bu+ state which leads to swapping of the state order. Thus, the authors attribute the experimentally observed involvement of a so called phantom state Sx to the change of character of the S2 PES from 1Bu+ to 1Bu-.

This is a well-executed work and a well-written paper. The arguments are compelling. I do not have any particular concerns and would recommend publishing addressing following comments:

1. The authors support their results on the relative postion of the 1Bu- state only with a theoretical work by Grimme (run with another type of semi-emprical method). Are there any experimental evidence (e.g. from two-photon absorption expeirments) or recent calculations? I recall having seen at least one recent DMRG/NEVPT2 study on carotenoides. It is highly recommended to extend the benchmarking against available data in the literature, if available, considering the important role of the 1Bu- state.

2. Could the authors address the dynamics of the beta-ionones? In my experience the two rings are considerably twiested at the GS equilibrium but tend to planarize towards the A2g- minimum as he rings conjugate stronger to the central chain. Do the authors observe such a dynamics?

3. By comparing dynamics in gas-phase and methanol the authors observe that polar solvents tend to speed up the internal conversion. Has this trend been observed experimentally? To what effect shoud one attribute this speed up in polar solvent? Is it electrostatics or rather sterics?

Reviewer: 3

Comments to the Author

The manuscript by Accomasso et al. reports a theoretical study of the excited states dynamics in the lutein carotenoid, unveiling the nature of the intermediate state Sx which mediates the internal conversion process. This state has been observed by many ultrafast experiments since quite long time,

however a detailed modelling and explanation on the mechanisms was still missing, thus such a contribution is very welcome in the community.

The data are well presented, and the general interpretation and conclusion are very clear convincing, however I have few comments/ suggestions that should be addressed before considering publication in this journal, here listed.

As general comment, to meet the criteria for publication, the urgency of the results presented should be highlighted, for instance why is it important to understand now the role of Sx state? How can this support future experimental studies? Also, I would encourage the authors to strengthen the significance and novelty of their work. At page 3 they claim that “nonadiabatic excited-state dynamics based on the mixed quantum-classical surface hopping (SH) method (…) for carotenoids is still missing in the literature”. “Why is it so and how they managed to overcome such limitations?

More specific points:

1. Based on their results reported in figure 4, can the authors propose conical intersections both at S2/Sx and at S1/S0?

2. In Figure 3, it seems clear that there are two close frequencies in the dynamics at Sx and S2 determining a beating oscillation, while this seems not to be the case for the dynamics at S1. Why is this so?

3. At pag 7 they say “Notably, the $1B^-_u$ energy is less sensitive than the $1B^+_u$ one to the BLA change,” but I do not understand why. Could they clarify this point?

4. Why in the diabatic basis 1Ag- and 2Ag- can be treated together? This is not clear to me.

5. The authors report the electronic coupling among the different states, but it could be possible to estimate the vibronic coupling as well?

6. In carotenoids, it has been also hypothesized the existence of a second intermediate state, S*, most likely interpreted as a vibrationally non-equilibrated ground state S0, or as a distinct electronic state in the energy vicinity of $S_1(1^1A_g^-)$, or as the S1 ($1^1A_g^-$) itself but with a twisted configuration. Did the authors also explore the possibility to also include this state in their analysis?

Author's Response to Peer Review Comments:

Reviewer 1

In this work the authors performed nonadiabatic excited-state dynamics calculations on lutein, a xanthophyll carotenoid found in the LHCII complex of green plants. Understanding the excited state dynamics of carotenoids is hindered by the presence of a debated number of optically dark states. Using mixed quantum-classical surface hopping calculations, Mennucci and coworkers identified the nature of one of the dark states, SX, as formed through a change in S2 character on the ~20 fs timescale, and furthermore identified the role of the SX state in mediating S2-S1 conversion on the sub-ps timescale. The work presented here will be of interest to those interested in natural light-harvesting and exciton dynamics, and thus should be published in The Journal of Physical Chemistry Letters after the following comments are addressed.

Authors' Reply: We thank the Reviewer for the positive comments.

Major Comments:

(1) The authors model lutein dynamics in the case of methanol and in the gas phase, however they also point to implications for light-harvesting. The authors should discuss how lutein dynamics would vary in this case according to (1) polarity conditions of the membrane protein environment (such as LHCII), and (2) variation in lutein structure within the protein pocket (thus giving rise to lutein 1 and lutein 2 structures).

Authors' Reply: We thank the reviewer for this suggestion. Indeed the protein environment can be considered as nonpolar or mildly polar, depending on the exact composition of the pocket. We can therefore assume an environment polarity in between gas-phase and methanol. As the results we obtained are similar in the two cases, we expect an analogous dynamics in protein. The only substantial difference, as the Reviewer suggests, is the steric constraints of the protein pocket, which give rise to different lutein structures (e.g. Lut1 and Lut2 in LHCII).
We added the following sentence to the second-to-last paragraph, where we discuss Lut in LHCII:
"As the polarity of LHCII is intermediate between gas-phase and highly polar methanol, we expect a similar excited-state dynamics to what we observed in our simulations. Steric constraints imposed by the protein may however influence the lutein excited

states and their dynamics. Efforts are underway in our group to simulate the ultrafast dynamics of lutein in pigment-protein complexes."

(2) On page 11, the authors note that a variation in potential energy surface curvature results in an increase in the C=C stretching mode of the S1 state. Is this trend reflective of the BLA frequency after the S1 state is formed (beyond the 200 fs timescale)?

Authors' Reply: We thank the Reviewer for raising this interesting point. We compared the frequencies between the ground-state dynamics (last 10 ps) and the SH simulations, by computing the power spectrum of the BLA (new figure S11 in the supporting information). In order to isolate the S1 state, we selected only those trajectories that stay in S1 for at least 175 fs. Although our simulations do not describe a thermal equilibrium, this analysis was sufficient to see the difference between S0 and S1 frequencies: the S0 BLA power spectrum peaks at around 1400 and 1650 cm^{-1}, whereas the S1 BLA power spectrum features a clear peak at 2100 cm^{-1}. This corresponds to a 27% increase in the C=C frequency.
We modified the main text accordingly:
"This effect causes a downshift in the vibrational frequencies of the C=C stretching mode in the S0 state, and a frequency upshift in the S1 state 50,51. We have monitored the frequency upshift by comparing the BLA power spectrum calculated on the S0 Born-Oppenheimer dynamics with the one calculated on those SH trajectories that remain at least 175 fs in the S1 state (Figure S11). In the S1 state, the BLA power spectrum features a peak at substantially higher frequencies compared to the S0 dynamics. "

(3) On page 13 the authors note, "Our simulations finally showed that high-frequency BLA modes are fundamental in driving the transition to the 1Bu−/SX state" However the BLA modes are on the order of 1700 cm-1, not the experimentally observed 1100-1500cm-1 (Llansola-Portoles et al. J. R. Soc. Interface, 2017, 14). The authors should address this discrepancy, and comment on how dynamics might change under the influence of lower frequency BLA oscillations.

Authors' Reply: We expect that the calculated frequencies are higher than the experiments because of the semiempirical approximation. In fact, we have parameterized our semiempirical method without explicitly targeting vibrational

properties, therefore this discrepancy is expected. From the computed ground-state BLA power spectrum (see response #2), which features peaks at around 1400 and 1650 cm^{-1}, we see that a scaling factor of around 0.85 and 0.90 should be applied to match the experimental frequencies of C-C and C=C modes.
However, the dynamics is not extremely sensitive to the exact value of the vibrational frequency, as the ultrafast internal conversion depends essentially on the presence of a crossing between surfaces. Therefore we do not expect a substantial change of dynamics in case the BLA frequencies were rescaled.

(4) According to Table 1, the 1Bu- state appears to have a non-zero oscillator strength in lutein, in contrast to the C20 polyene. The authors should comment on the origin of this deviation from total dark state character.

Authors' Reply: The breaking of the C2h symmetry in going from C20 polyene to lutein leads to a partial mixing of the Bu+ and Bu- electronic configurations. As a result, while the bright H -> L configuration does not contribute to the S3 state of C20 polyene, in lutein such configuration provides a non-negligible contribution to S3 (i.e. 0.03% of the S3 wavefunction).

(5) As discussed in reference 51, The Sx state is assigned through a torsional motion to the S2 state. While this bond torsion is not observed, a partial intramolecular charge-transfer state is also implicated in reference 51- do this authors observe significant redistribution of electron density to support the potential impact of an intramolecular charge-transfer state?

Authors' Reply: Following the Reviewer's suggestion, we have computed the ICT character as the charge of one of the two halves of the molecule, compared to the ground state. This quantity was computed along the active state of each SH trajectory. We have not seen a significant difference compared to the ground state, which points against the involvement of a ICT state within the first 200 fs.

Reviewer 2

Accomasso et al. present on the fly mixed quantum-classical dynamics simulation of the carotenoid lutein in methanol. The electronic structure of the solute is computed at the semi-empirical level, reparamterized to reproduce a number of parameters (transition energies, TDMs, geometircal parameters) whereas the interaction with the solvent is treated within a QMMM framework. The semi-emprical calculations allow to run a comparably big number 200 trajectories and collect reliable statistics. The main finding of this work which represents one of the first dynamical studies on carotenes, is the involvement of a higher lying spectroscopically dark state (1Bu-) besides the usual suspects 2Ag- (the lowest lying dark hh->l,l state) and 1Bu+ (the lowest bright state with h->l character). It is found that this state exhibits a more pronounced stabilization along the BLA coordinate wrt the 1Bu+ state which leads to swapping of the state order. Thus, the authors attribute the experimentally observed involvement of a so called phantom state Sx to the change of character of the S2 PES from 1Bu+ to 1Bu-. This is a well-executed work and a well-written paper. The arguments are compelling. I do not have any particular concerns and would recommend publishing addressing following comments:

Authors' Reply: We thank the reviewer for the positive comments

1. The authors support their results on the relative position of the 1Bu- state only with a theoretical work by Grimme (run with another type of semi-emprical method). Are there any experimental evidence (e.g. from two-photon absorption experiments) or recent calculations? I recall having seen at least one recent DMRG/NEVPT2 study on carotenoides. It is highly recommended to extend the benchmarking against available data in the literature, if available, considering the important role of the 1Bu- state.

Authors' Reply: The Reviewer is right in that other calculations have been performed including the $1B_u^-$ state for model carotenoids, including refs. 32, 34 and 35 in the original manuscript. In particular, Khohklov et al. (JCTC 2021) have employed a mixed *ab initio* strategy which confirms that the minimum of the $1B_u^-$ PES is below the $1B_u^+$ minimum for polyenes with 8 or more double bonds.
Experimentally, Raman excitation profiles measured for carotenoids of different conjugation lengths have suggested that the $1B_u^-$ adiabatic energy is lower than $1B_u^+$

for carotenoids with β-Ionone rings and at least 8 double bonds (Sashima et al., *J. Phys. Chem. B* (2000), 104, 5011–5019.).
We included references to these results in our discussion:
"Moreover, *ab initio* DMRG/MRPT2 calculations have suggested that the adiabatic $1B_u^-$ energy is lower than the $1B_u^+$ one for carotenoids with 8 or more double bonds. A similar result is suggested by experimental Raman excitation profiles."

2. Could the authors address the dynamics of the beta-ionones? In my experience the two rings are considerably twisted at the GS equilibrium but tend to planarize towards the A2g- minimum as the rings conjugate stronger to the central chain. Do the authors observe such a dynamics?

Authors' Reply: We have not observed a significant change in the beta-ionone ring orientation with respect to the backbone chain within the SH simulations employed here. One reason could be a limitation of our semiempirical method. However, we might not see a rotation of the rings because we have simulated only the ultrafast part of the dynamics, and this motion could occur at a later time.

3. By comparing dynamics in gas-phase and methanol the authors observe that polar solvents tend to speed up the internal conversion. Has this trend been observed experimentally? To what effect should one attribute this speed up in polar solvent? Is it electrostatics or rather sterics?

Authors' Reply: Most experiments on lutein have been conducted in low polar solvents, so a quantitative evaluation of the role of polarity is not possible. From our calculations, the faster 1Bu+ -> 1Bu- transfer of population in methanol, compared to the gas-phase simulations, seems to be due to the larger 1Bu+/1Bu- average coupling, both at the starting geometries and at the 1Bu+ -> 1Bu- transitions (compare Tables S4 and S13). On the other hand, the average energy gap between 1Bu+ and 1Bu- does not change significantly in going from methanol to gas phase.
We added the following sentence to the main text:
"The larger internal conversion rate in methanol, compared to the gas-phase simulations, can be mainly attributed to the faster 1Bu+ -> 1Bu- transfer of population, which in turn is due to the larger 1Bu+/1Bu- average coupling, both at the starting geometries and at the 1Bu+ -> 1Bu- transitions (compare Tables S4 and S13)."

Reviewer 3

The manuscript by Accomasso et al. reports a theoretical study of the excited states dynamics in the lutein carotenoid, unveiling the nature of the intermediate state Sx which mediates the internal conversion process. This state has been observed by many ultrafast experiments since quite long time, however a detailed modelling and explanation on the mechanisms was still missing, thus such a contribution is very welcome in the community.
The data are well presented, and the general interpretation and conclusion are very clear convincing, however I have few comments/ suggestions that should be addressed before considering publication in this journal, here listed.

Authors' Reply: We thank the reviewer for the positive comments

As general comment, to meet the criteria for publication, the urgency of the results presented should be highlighted, for instance why is it important to understand now the role of Sx state? How can this support future experimental studies? Also, I would encourage the authors to strengthen the significance and novelty of their work. At page 3 they claim that "nonadiabatic excited-state dynamics based on the mixed quantum-classical surface hopping (SH) method (...) for carotenoids is still missing in the literature". "Why is it so and how they managed to overcome such limitations?

Authors' Reply: We thank the Reviewer for this suggestion. Indeed, the electronic structure of carotenoids is quite complicated, and *ab initio* multireference methods are costly for very large molecules (lutein has 20 pi electrons). The need to describe states with very different character (ionic and covalent) in a balanced way further complicates the search for a good *ab initio* method. Here, we could overcome these limitations by employing a specifically parametrized semiempirical method. In particular, the FOMO-CI method well describes the multireference character of the states, and the parameter tuning ensures a balanced description of all excited states.
We added the following sentences to the paragraph mentioned by the Reviewer:
"In fact, the molecular dimensions of the carotenoids, the number of correlated electrons, and the presence of states with different character make ab initio descriptions extremely costly and unsuitable for nonadiabatic dynamics."

More specific points:

1. Based on their results reported in figure 4, can the authors propose conical intersections both at S_2/S_x and at S_1/S_0?

Authors' Reply: We thank the Reviewer for this suggestion. Indeed, the back-and-forth population transfer between S2 and S3 states (in the adiabatic picture) quite clearly occurs through a conical intersection, around BLA = 0.02 Å. In the diabatic picture, this corresponds to the system remaining in the same diabatic state (1Bu+ or 1Bu-) after the crossing. This is consistent with the small energy gaps encountered at the transitions between these states (Table S4).

On the other hand, the S1/S0 surfaces remain far from each other due to the large diabatic coupling among the two Ag- states. The conical intersection geometry is thus likely to be very distorted, and not easily reached within the time frame of our simulations.

We have computed nonadiabatic couplings for a representative SH trajectory (see response #5). We see that only the S2/S3 nonadiabatic coupling increases substantially at the beginning of the trajectory (i.e. when approaching the 1Bu+/1Bu- crossing), whereas the S1/S0 coupling remains small, indicating that the system is far from a conical intersection.

2. In Figure 3, it seems clear that there are two close frequencies in the dynamics at S_x and S_2 determining a beating oscillation, while this seems not to be the case for the dynamics at S_1. Why is this so?

Authors' Reply: We thank the Reviewer for raising this point. The beatings between two different frequencies are indeed present also in the BLA, as can be seen around 100 fs in Figure 3 (bottom). These beatings are present also in S1/2Ag-, although they are not so evident, while they are much more noticeable for 1Bu+ and 1Bu-.

The beatings arise from several reasons. Firstly, both C-C and C=C modes are activated by the initial excitation, and, as they feature two quite close frequencies, they generate beatings. Secondly, the transfer of population to the lower excited states within the dynamics also changes the "average" frequency of the modes. We recall in fact that the data in Figure 3 are obtained as an average over all SH trajectories, regardless of the electronic state. Finally, as the system leaves the Franck-Condon region, normal modes can mix and transfer energy, and due to anharmonicity the diabatic excitation energies may become more (or less) sensitive to the normal modes.

The reason why the beatings are more visible in the 1Bu+ and 1Bu- energies may lie in the fact that the energies of these diabatic states are more sensitive to some modes whereas the 1Ag- energy is sensitive to other modes.

3. At pag 7 they say “Notably, the $1B^{-}_{u}$ energy is less sensitive than the $1B^{+}_{u}$ one to the BLA change,” but I do not understand why. Could they clarify this point?

Authors’ Reply: We apologize for the confusion, and we thank the Reviewer for noticing this inconsistency. We meant to state the opposite: the $1B^{-}_{u}$ energy is more sensitive than the $1B^{+}_{u}$ energy upon a change of the BLA.
We have corrected the sentence in the revised manuscript.

4. Why in the diabatic basis $1Ag^{-}$ and $2Ag^{-}$ can be treated together? This is not clear to me.

Authors’ Reply: We thank the Reviewer for raising this point. Indeed, the reason for treating these two states together is given in a later paragraph. In fact, the two diabatic states are significantly coupled, especially when approaching the S1 minimum. The diabatic coupling (~0.5 eV) is comparable to the diabatic energy gap, which means that the S1 state is a mix of 1Ag- and 2Ag-.
The diabatic picture is extremely useful to figure out what happens in the ultrafast excited-state dynamics between $1B^{-}_{u}$ and $1B^{+}_{u}$, especially because these states are weakly coupled: as far as we are not in the crossing seam, the diabatic and adiabatic surfaces are indistinguishable (See Figure 4). On the other hand, because of the large couplings between 1Ag- and 2Ag-, these states are strongly mixed for most of the excited-state dynamics, and it would not be correct to treat them separately. In fact, in Figure 2b the population of these two diabatic states follow the same time evolution, which is essentially the evolution of the adiabatic S1 population.
We added a sentence to the paragraph that comments the diabatic mixing. Now the paragraph reads:
“[...] The strong mixing makes the adiabatic S0 and S1 PESs much different from their diabatic counterparts. Given this mixing, in the diabatic analysis we have considered the two Ag- states together.”

5. The authors report the electronic coupling among the different states, but it could be possible to estimate the vibronic coupling as well?

Authors' Reply: We have estimated vibronic coupling as the nonadiabatic coupling between adiabatic states, which we computed along a representative SH trajectory (Figure S10). We see that the nonadiabatic coupling is highly sensitive to the energy gap between states. In our simulations, only the S2/S3 nonadiabatic coupling becomes large, in the first 30-40 fs, while the S2/S1 and S1/S0 couplings remain small.
We added the following sentence to the main text:
"Such transition geometries are characterized by large values of the S2/S3 nonadiabatic coupling (Figure S10)."

6. In carotenoids, it has been also hypothesized the existence of a second intermediate state, S*, most likely interpreted as a vibrationally non-equilibrated ground state S0, or as a distinct electronic state in the energy vicinity of S_1 ($1^1A_g^-$), or as the S_1 ($1^1A_g^-$) itself but with a twisted configuration. Did the authors also explore the possibility to also include this state in their analysis?

Authors' Reply: We agree with the Reviewer that the S* state is important in the long(er) time dynamics of carotenoids. Indeed, the nature of this state is still debated. Within the timescales explored by our simulations, we have not detected the presence of a distinct electronic state (i.e. in addition to 1Ag-/2Ag-/1Bu+/1Bu-). On the other hand, if the S* spectral features arise from S1 in a different geometry, a longer (ps-scale) dynamics would be needed to explore the configurations along the S1 potential energy surface, especially because the S* features seem to be longer lived than the S1 ones.
